# High-pressure processing-induced transcriptome response during recovery of *Listeria monocytogenes*

**DOI:** 10.1186/s12864-021-07407-6

**Published:** 2021-02-12

**Authors:** Ilhan Cem Duru, Florentina Ionela Bucur, Margarita Andreevskaya, Bahareh Nikparvar, Anne Ylinen, Leontina Grigore-Gurgu, Tone Mari Rode, Peter Crauwels, Pia Laine, Lars Paulin, Trond Løvdal, Christian U. Riedel, Nadav Bar, Daniela Borda, Anca Ioana Nicolau, Petri Auvinen

**Affiliations:** 1 Institute of Biotechnology, University of Helsinki, Helsinki, Finland; 2 Faculty of Food Science and Engineering, Dunarea de Jos University of Galati, Galati, Romania; 3 Department of Chemical Engineering, Norwegian University of Science and Technology (NTNU), Trondheim, Norway; 4 Department of Process Technology, Nofima – Norwegian Institute of Food, Fisheries and Aquaculture Research, N-4068 Stavanger, Norway; 5 Institute of Microbiology and Biotechnology, Ulm, University, Albert-Einstein-Allee 11, D-89081 Ulm, Germany

**Keywords:** Time-series RNA-seq, Stress recovery, Rli47, Food pathogen, Sigma factor B

## Abstract

**Background:**

High-pressure processing (HPP) is a commonly used technique in the food industry to inactivate pathogens, including *L. monocytogenes*. It has been shown that *L. monocytogenes* is able to recover from HPP injuries and can start to grow again during long-term cold storage. To date, the gene expression profiling of *L. monocytogenes* during HPP damage recovery at cooling temperature has not been studied. In order identify key genes that play a role in recovery of the damage caused by HPP treatment, we performed RNA-sequencing (RNA-seq) for two *L. monocytogenes* strains (barotolerant RO15 and barosensitive ScottA) at nine selected time points (up to 48 h) after treatment with two pressure levels (200 and 400 MPa).

**Results:**

The results showed that a general stress response was activated by SigB after HPP treatment. In addition, the phosphotransferase system (PTS; mostly fructose-, mannose-, galactitol-, cellobiose-, and ascorbate-specific PTS systems), protein folding, and cobalamin biosynthesis were the most upregulated genes during HPP damage recovery. We observed that cell-division-related genes (*divIC*, *dicIVA*, *ftsE*, and *ftsX*) were downregulated. By contrast, peptidoglycan-synthesis genes (*murG*, *murC*, and *pbp2A*) were upregulated. This indicates that cell-wall repair occurs as a part of HPP damage recovery. We also observed that prophage genes, including anti-CRISPR genes, were induced by HPP. Interestingly, a large amount of RNA-seq data (up to 85%) was mapped to Rli47, which is a non-coding RNA that is upregulated after HPP. Thus, we predicted that Rli47 plays a role in HPP damage recovery in *L. monocytogenes*. Moreover, gene-deletion experiments showed that amongst peptidoglycan biosynthesis genes, *pbp2A* mutants are more sensitive to HPP.

**Conclusions:**

We identified several genes and mechanisms that may play a role in recovery from HPP damage of *L. monocytogenes*. Our study contributes to new information on pathogen inactivation by HPP.

**Supplementary Information:**

The online version contains supplementary material available at 10.1186/s12864-021-07407-6.

## Background

*Listeria monocytogenes* is a foodborne bacterial pathogen that poses a particular challenge to the food industry due to its ubiquitous nature and capability of adapting to various inhospitable environmental conditions related to food matrices and food processing environments [1–3]. Transmission of this bacterium to humans generally occurs via consumption of contaminated food, especially ready-to-eat (RTE) foods that do not undergo thermal treatment during the manufacturing process, such as sliced and packed meat products, RTE salads, dairy products from raw milk, vegetables, and fruits. *L. monocytogenes* can cause listeriosis, a disease associated with a high number of hospitalization cases and mortality rates of 20–30% among people with weakened immune systems [4]. *L. monocytogenes* can resist a wide range of environmental conditions [5] and its ability to grow at refrigeration temperatures increases the risk of listeriosis [6].

The increasing demand of consumers for minimally processed foods, with fresh-like sensorial and nutritional properties, requires the implementation of alternative food processing techniques such as high-pressure processing (HPP). HPP is a relatively new, non-thermal processing technique that shows remarkable results with respect to pathogen inactivation and minimum impact on food quality [7, 8].

It has been reported that HPP causes morphological, structural, physiological, and genetic changes or damages to *L. monocytogenes* cells [9]. However, the susceptibility of *L. monocytogenes* to HPP varies between growth phase [10], strength of the cellular envelope [11], genomic features [12], and individual strains [13]. In addition, food matrix type, temperature, water activity, compression and decompression rates, applied pressure and holding time, and other extrinsic factors have an impact on inactivation of *L. monocytogenes* cells by HPP [9]. Several studies reported the potential of sublethally injured *L. monocytogenes* cells to recover after HPP and grow within the storage period even under refrigeration conditions [14–18]. Bozoglu et al. [19] showed that sublethally injured bacteria could not be detected immediately after HPP treatment of up to 550 MPa. However, injured but viable cells may be present in the pressurised samples as the authors detected growth after 6 days at 4 °C and already after 1 day at 22 °C and 30 °C.

Therefore, for an efficient decontamination process, additional hurdles to increase efficiency of HPP and/or to prevent outgrowth of sublethally injured bacteria should be considered. In this context, it may be of interest to treat *L. monocytogenes* cells with antimicrobial agents that compromise cell wall and/or membrane and thereby render bacteria more sensitive to HPP and inhibit recovery. Such antimicrobial agents may include bacteriocins [20], essential oils [21–23], plant extracts [24], bacteriophages [25, 26], lysozyme [27, 28], lactoferrin [29], and lactoperoxidase [30].

Gene expression profiling of the response of *L. monocytogenes* to HPP has previously been studied by RNA-seq [12], microarray [31, 32], and qPCR [33]. For example, it has been shown that a mutation in *ctsR* causes barotolerance and a *ctsR* deletion mutant of *L. monocytogenes* shows increased expression of Clp protease and PTS system genes after HPP [31]. Similarly, we previously reported that heat-shock and Clp protease family genes were upregulated after HPP [12]. In contrast, Bowman et al. [32] reported downregulation of heat-shock and PTS system genes after HPP. The previous studies used relatively higher temperatures for storage after HPP (⩾ 15 °C) compared to common cold-storage applications in the food industry. We recently showed genetic differences between barotolerant and barosensitive *L. monocytogenes* strains, which may explain their different HPP sensitivity [12]. Hence, in the present study, we investigate the transcriptional response to HPP and the differences in gene expression profiles between barotolerant and barosensitive *L. monocytogenes* strains during recovery. We selected *L. monocytogenes* strains RO15 (barotolerant) and ScottA (barosensitive) that were already analysed previously [12]. This is the first study to perform time-series RNA-seq using both barosensitive and barotolerant strains monitoring gene expression profiles during recovery of an HPP insult at 8 °C. We aimed to identify candidate genes that would be involved in the recovery of *L. monocytogenes* after HPP treatment.

## Results

### Log reduction testing of the strain RO15 and ScottA

**Fig. 1:**
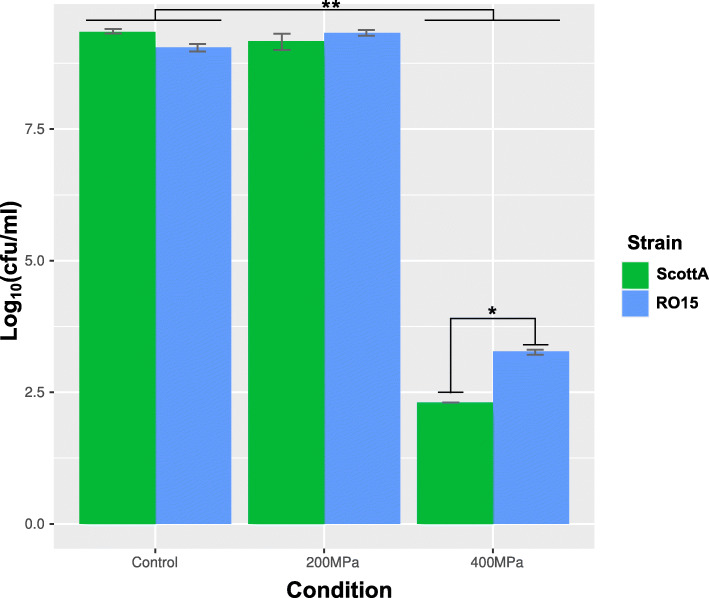
Viable cell count in log_10_(cfu/ml) of strain ScottA (green bars) and RO15 (blue bars) after 200 and 400 MPa for 8 min at 8 °C. Samples are triplicate (*n* = 3). *: Student’s t-test *p*-value < 0.05 between ScottA and RO15 log reduction. **: Student’s t-test p-value < 0.05 between control and 400 MPa log (cfu/ml) in both strains

We previously reported that strain RO15 is more resistant to treatment of 400 MPa for 1 min compared to several other *L. monocytogenes* strains including strain ScottA [12]. Here, we sought to test the susceptibility of both strains to pressure treatment at 200 and 400 MPa for 8 min at 8 °C. While a treatment with 200 MPa was ineffective for inactivation of both strains, 400 MPa significantly reduced log_10_(cfu/ml) for both RO15 (5.78 log_10_ reduction) and ScottA (7.04 log_10_ reduction) compared to untreated samples (*p* < 0.05). The log reduction difference between the two strains was also statistically significant (*p* < 0.05; Fig. 1).

### Differential expression analysis

After the HPP treatments, samples were taken at nine time points (0, 5, 10, 30, 45, 60 min and 6, 24, 48 h) and RNA-seq performed. Principal component analysis (PCA) of per gene read count data showed that there was a clear separation between HPP-treated samples and control samples for both 200 MPa and 400 MPa (Figure S1). In addition, we also saw clustering between early time points (0, 5, and 10 min), middle time points (30, 45, and 60 min), and late time points (6, 24, and 48 h) for 200 MPa treatment on a PCA plot (Figure S1).

Pairwise differential expression analysis between the treated samples and corresponding controls at all time points showed that a large number of genes were significantly differentially expressed (padj-value ≤0.05, |log2 fold change| > 0.6) after HPP (Figure S2, Table S1, S2, S3, S4). Depending on the time point and pressure applied, between 104 and 420 genes were downregulated and between 152 to 45 genes were upregulated in RO15 with a log_2_ fold change range of − 6.93 to 7.07. For ScottA, between 233 and 404 genes were upregulated and between 188 and 352 genes were downregulated with a log_2_ fold change range of − 6.37 to 8.25 (Figure S2, Table S1, S2, S3, S4).

### Differentially expressed genes and GO enrichment analysis

**Fig. 2:**
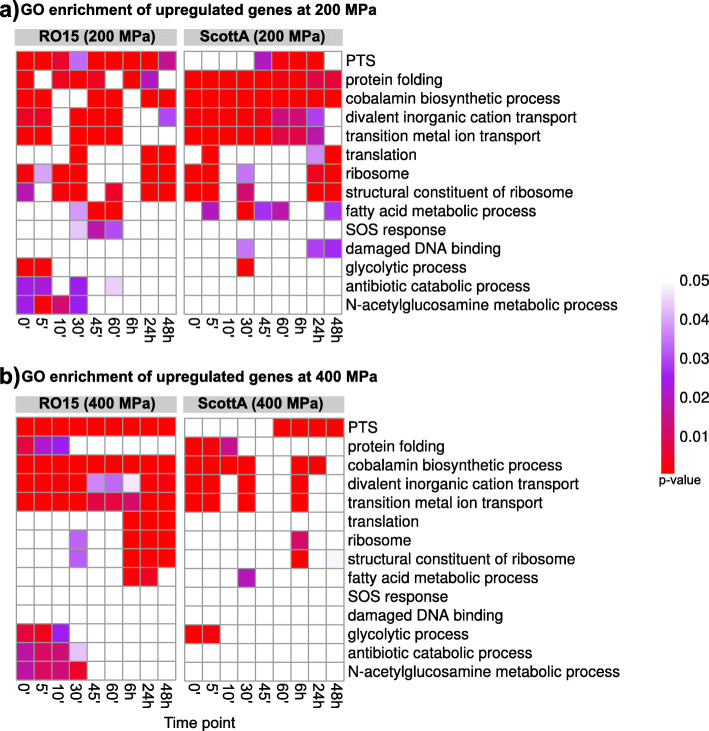
Heat maps of gene ontology (GO) terms enriched in upregulated genes at different time points after HPP treatment at a) 200 MPa and b) 400 MPa of *L. monocytogenes* ScottA or RO15. Statistical significance of the GO enrichment (*p*-values ≤0.05) are indicated by a colour gradient (increasing red colour intensity) indicated at the right side of the heat maps). White colour indicates that the GO term was not significantly enriched (*p*-values > 0.05). For the sake of simplicity, the figure does not include all enriched GO terms, all enriched terms are provided in Table S5

To gain a general perception of the functional groups of the differentially expressed genes, GO enrichment was performed (Fig. 2, Table S5). The most significantly enriched GO terms for upregulated genes were cobalamin biosynthetic process, divalent inorganic cation transport, and transition metal ion transport for both strains (Fig. 2). These GO terms were enriched at several time points in both strains after 200 and 400 MPa treatment. The main upregulated genes responsible for these GO terms enrichment were found in a large operon (OCPFDLNE_01234 - OCPFDLNE_01251 in the RO15 strain, LMOSA_20560 - LMOSA_20730 in the ScottA strain), including cobalamin biosynthesis genes, Cobalt ABC transporter, and Cobalt transport-related genes (Figure S3).

In *L. monocytogenes* RO15 HPP-upregulated genes were enriched at most time points in GO terms “phosphoenolpyruvate-dependent sugar phosphotransferase system (PTS system)” and “carbohydrate transmembrane transport” both after the 200 and 400 MPa treatment (Fig. 2, Table S5). In detail, upregulation was observed for the genes for fructose-, mannose-, galactitol-, cellobiose-, and ascorbate-specific PTS systems. Enrichment of these GO terms was also seen in the HPP-treated samples of *L. monocytogenes* ScottA strain taken, however, at later time points after HPP (both 200 and 400 MPa) (Fig. 2).

**Fig. 3:**
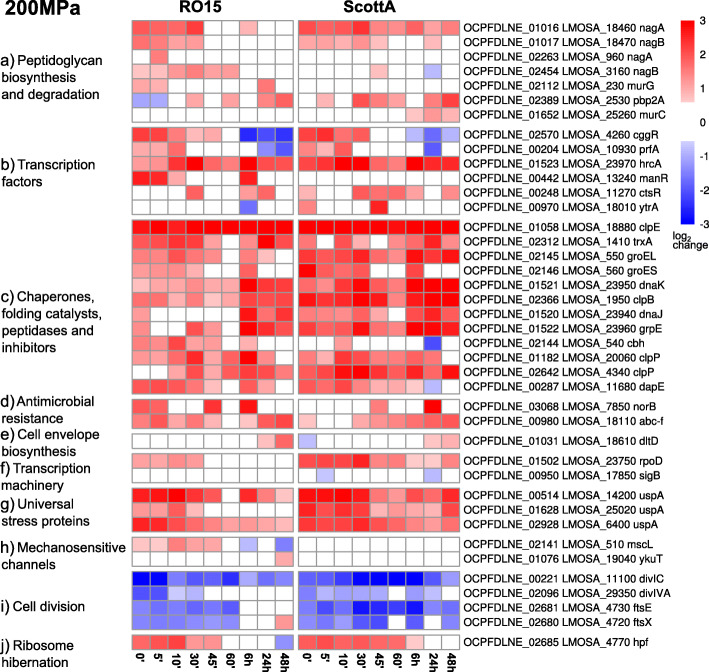
Heat maps with log_2_ fold-change of selected genes in selected gene families in *L. monocytogenes* RO15 or ScottA after 200 MPa pressure treatment. Gene names and locus tags for RO15 and ScottA are indicated at the end of each row. Log_2_ fold-change scale is shown in the right corner

**Fig. 4:**
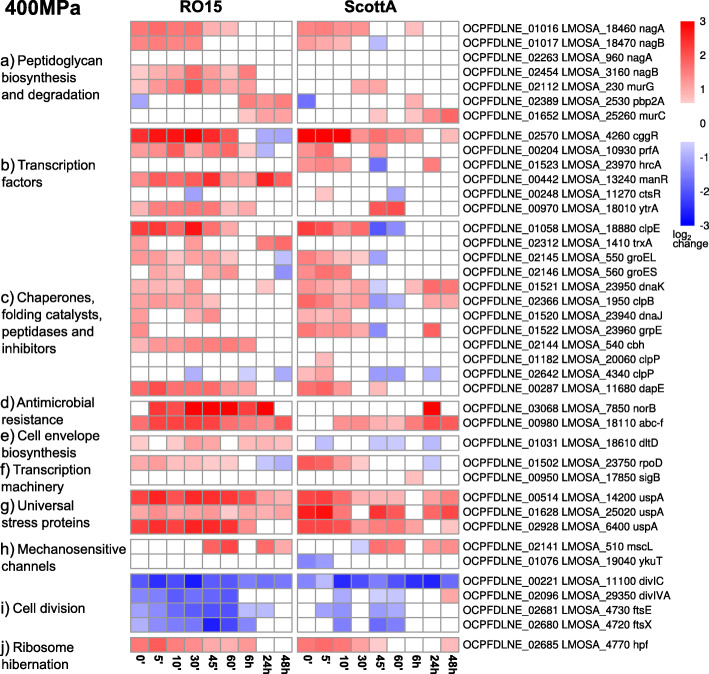
Heat maps with log_2_ fold changes of selected genes of selected gene families in *L. monocytogenes* RO15 or ScottA after 400 MPa pressure treatment. The gene name and locus tag of genes for RO15 and ScottA are given at the end of the row. The log_2_ fold change scale is shown in the right corner

HPP caused upregulation of protein folding, chaperone, and peptidases genes, such as *clpE*, *clpP*, *groEL*, *groES*, *hrcA*, *dnaK*, and *dnaJ*, at 200 MPa at almost all time points and at 400 MPa at the early time points as reflected by an enrichment in the GO term “protein folding” (Fig. 2, 3c, 4c).

Significant downregulation was observed for cell division genes *divIC*, *dicIVA*, *ftsE*, and *ftsX* (Fig. 3i, 4i). In addition, in downregulated genes we observed a significant (*p* < 0.05) enrichment of the GO term “ATPase activity” (Table S5) at almost all time points for both pressure levels.

In addition to enriched GO terms, RNA-seq data was also analysed for specific gene families such as transcription factors (Fig. 3b and 4b, Figure S4) and transcription machinery genes (Fig. 3f and 4f, Figure S5). The gene *hrcA* (encoding the heat-inducible transcription repressor HrcA) was upregulated at all time points for both RO15 and ScottA strains after 200 MPa, while after 400 MPa treatment upregulation was seen only in ScottA for the early time points (Fig. 3b, 4b). We also observed that gene *prfA* encoding the master regulator of virulence genes in *L. monocytogenes* was upregulated in both strains after both pressure treatments (Fig. 3b, 4b). Interestingly, only one of the *manR* genes encoding the transcriptional regulator ManR, which is found in two different copies in the genomes of ScottA and RO15, was upregulated in RO15 strain after HPP treatment but not in ScottA (Fig. 3b and 4b). With respect to the transcription machinery genes, *rpoD* encoding the RNA polymerase sigma factor RpoD was upregulated in both strains after HPP (Fig. 3f and 4f).

Upregulation of *nagA* (OCPFDLNE_01016) and two separate *nagB* genes (OCPFDLNE_01017 and OCPFDLNE_02454) was observed in RO15 at all time points. Similarly, ScottA showed upregulation of the nagA (LMOSA_18460) and *nagB* (LMOSA_18470) homologues at early time points. However, the second *nagB* homologue of ScottA (LMOSA_3160) was not upregulated (Fig. 3a, 4a).

We also focused on mechanosensitive channel genes. The gene encoding a putative mechanosensitive channel gene of large conductance (*mscL*) was upregulated at early time points after 200 MPa treatment in RO15, while this upregulation was not seen in ScottA (Fig. 3h). After 400 MPa, both strains had similar *mscL* expression patterns with upregulation at late time points (Fig. 4h). The homologue for a mechanosensitive channel of small conductance (*ykuT*) was only upregulated in RO15 at 48 h after 200 MPa treatment (Fig. 3h).

To see the difference between the responses to the different pressure levels, we identified genes that were upregulated after 200 MPa treatment but not after 400 MPa and vice versa for each time point. In both strains, the genes that were upregulated after 200 MPa but not after 400 MPa at early time points were mainly related to translation (Table S6, Table S7). Interestingly, translation-related genes were upregulated after 400 MPa but not after 200 MPa in the RO15 on late time points (Table S6, Table S7). We observed upregulation of *hpf* gene (encoding ribosome hibernation promoting factor) in ScottA even at the time point 48 h (Fig. 4j). In addition, we also observed several cobalamin biosynthesis and PTS-related genes were upregulated at early time points after 400 MPa but not after 200 MPa in both strains (Table S6, Table S7).

Genes without orthologs within both strains were mainly phage genes and hypothetical genes. In both strains, phage genes were mostly upregulated after HPP (Figure S6, Figure S7). We previously reported that barotolerant strains harbour both CRISPR-Cas systems and anti-CRISPR genes [12]. However, upregulation of Cas genes was observed in neither of the two strains whereas anti-CRISPR genes (*acrIIA1* and *acrIIA2*) were significantly upregulated after HPP in RO15 (Figure S6).

### Non-coding RNA (ncRNA)

RNA-seq read coverage plots showed that a very large amount of RNA-seq reads were mapped to non-coding regions, especially for RO15. Further examination showed that, on average, ~ 53% (ranging from 21 to 86%) of all RNA-seq reads for RO15 samples were mapped to the small non-coding RNA (ncRNA) Rli47. Similarly, ~ 28% (ranging from 6 to 72%) of the RNA-seq reads in samples of ScottA mapped to Rli47 (Table S8, Table S9). Thus, we additionally performed expression analysis of ncRNAs. We observed that *Rli47* transcript levels were upregulated in response to pressure treatment in both strains (Figure S8). Similarly, levels of *LhrA* ncRNA were upregulated in both strains at the early time points. Interestingly, expression of *Rli53* was upregulated in RO15 after the pressure treatment, while no upregulation was seen in ScottA.

### Gene regulatory networks based on RNA-seq data

One of our goals was to understand the regulatory networks involved in the response to HPP treatment in *L. monocytogenes* strains, RO15 and ScottA. Consensus gene network was created using the time-series expression data for all differentially expressed genes in both strains. This resulted in a total of 3661 gene network links (1506 genes and 3661 edges) for strain RO15 and 3427 gene network links (1389 genes and 3427 edges) for strain ScottA (Table S10). Interactive visualizations can be seen on https://icemduru.github.io/RO15_gene_network and https://icemduru.github.io/ScottA_gene_network. Moreover, we clustered the genes based on the network data (Table S10) using network clustering algorithm Map equation [34]. For RO15, 151 clusters were predicted in the gene network (Table S11), while for ScottA, 128 clusters were predicted (Table S12).

**Fig. 5:**
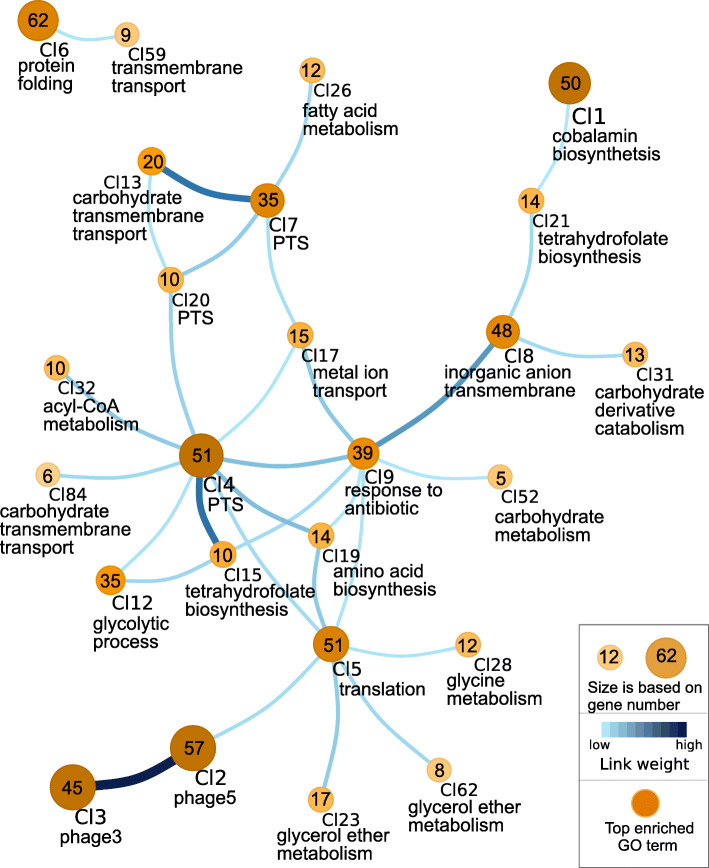
Visualization of clustered genes based on gene network inference in *L. monocytogenes* RO15. Each node represents a cluster and edges represent predicted links between clusters. Number of genes within the clusters is shown in the center of the node. Below the cluster, the top-scored enriched GO term is given. The used scales are described in the box. Size and colour are based on gene number. For simplification, the figure shows only the top 30 links with the highest weight, and their connected clusters. Genes within the clusters, and all links between clusters, are listed in Table S11

**Fig. 6:**
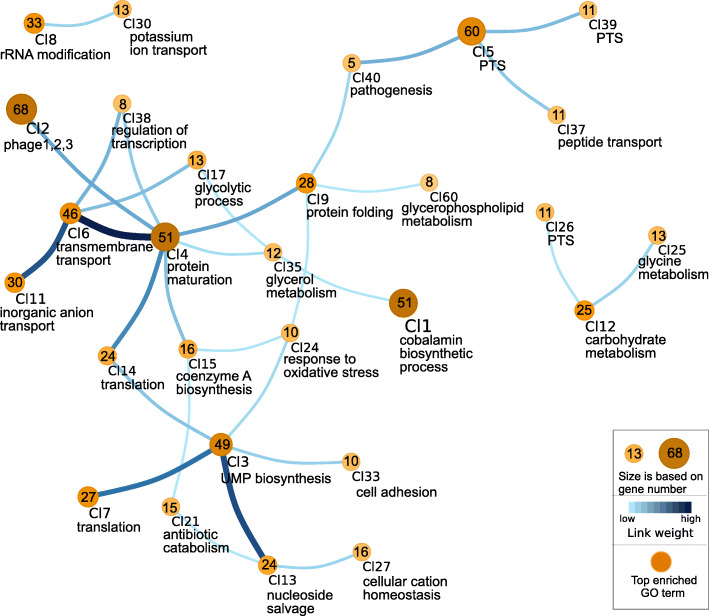
Visualization of clustered genes based on gene network inference in *L. monocytogenes* ScottA. Each node represents a cluster and edges represent predicted links between clusters. Number of genes within the clusters is shown in the center of the node. Below the cluster, top-scored enriched GO term is given. The used scales are described in the box. For simplification, the figure shows only the top 30 links with the highest weight, and their connected clusters. Genes within the clusters, and all links between clusters are listed in the Table S12

For both strains, heat shock and chaperone-related genes were clustered together (Cl6 in RO15 and Cl9 in ScottA, Figs. 5, 6). De novo motif discovery analysis resulted in a number of significant motifs (E-value < 0.05) for the upstream regions of heat shock clusters (Cl6 in RO15 and Cl9 in ScottA), and one of the motif was significantly (E-value < 0.05) similar to the CtsR motif (Table S13, Table S14) from the PRODORIC database [35] in both strains. This indicates CtsR is a regulator for protein-folding genes in these strains. We also observed that *ctsR* was linked to heat-shock genes based on gene network inference (Table S11, Table S12). Notably, *nagA* and *nagB* are placed in the heat shock cluster (Cl9) in ScottA providing a hint that co-expression of protein folding genes and peptidoglycan biosynthesis genes after the pressure treatment was required together for recovery in ScottA. In addition, we observed that the heat-shock cluster (Cl9) in ScottA was linked to Cl4 (Fig. 6), which includes stress-related genes.

The prophage genes were highly interconnected in both strains; almost all genes of the three phages found in the same cluster in ScottA (Cl2, Fig. 6) indicate all three prophages show similar gene expression reactions in ScottA. Similarly, prophage 3 and prophage 5 genes were highly linked in RO15 (Cl2 and Cl3, Fig. 5). The prophage cluster (Cl2) was also linked to Cl4 (Fig. 6) in ScottA, which contains universal stress protein UspA genes (*uspA*), indicating that phage induction was connected to stress response in ScottA. The prophage cluster (Cl3) was linked to Cl5 in RO15 (Fig. 5), which includes *mscL*, i.e. the gene for a mechanosensitive channel gene of large conductance, and *cspA* encoding a cold-shock protein.

The genes of Cl9 in RO15 were enriched for The GO term “response to antibiotic”. This cluster also included genes *uspA* (for universal stress protein A), virulence factor *prfA* (the master regulator of virulence in *L. monocytogenes*), and *hpf* (ribosome hibernation promoting factor), which all were significantly upregulated in both strains after HPP treatment. A similar cluster containing *prfA and hpf* was seen in ScottA (Cl6). This implies that stress response, virulence and ribosome hibernation are linked to each other and co-occurred with HPP treatment in both strains. For ScottA, de novo motif discovery for the upstream region of Cl6 genes resulted in two significant motifs (E-value < 0.05; Table S14), one of them being significantly (E-value < 0.05) similar to SigB motif from PRODORIC database [35].

### ddPCR validation of RNA-seq results

ddPCR was performed for the same ScottA samples that were used for RNA-seq to validate the differential expression results obtained using RNA-seq data. Nine genes were selected (Table S15) and three different time points were tested; 200 MPa time point 24 h (Figure S9), 400 MPa time point 10 min (Figure S10) and 24 h (Figure S11). ddPCR results for 8 selected genes showed that the Pearson correlation was very strong (> 0.97) between ddPCR log_2_ fold change and RNA-seq log_2_ fold change results for all time points.

### The influence of HPP on mutant strains` viability

**Fig. 7:**
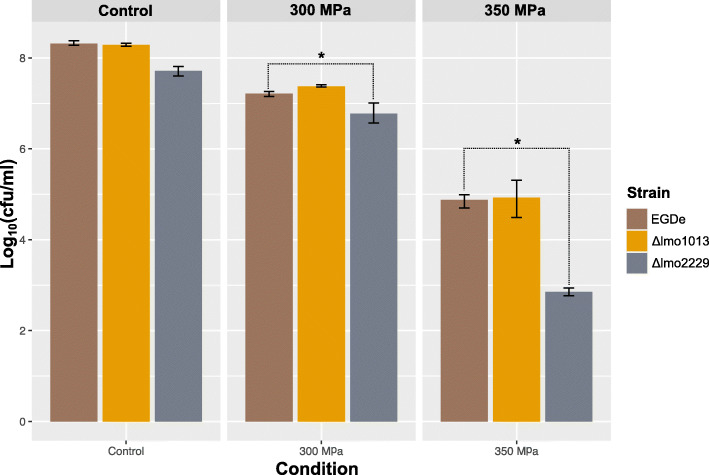
Effect of HPP treatments on the viability of *L. monocytogenes* EGDe, *lmo1013* and *lmo*2229 deletion mutants. The cells were grown at 37 °C, in TSB with 0.6% YE and subjected to HPP at 300 and 350 MPa for 5 min. The results are shown for the wild-type EGDe (brown bar), Δ*lmo1013* (orange bar), and Δ*lmo2229* (gray bar). *: Student’s t-test p-value < 0.05 against wild-type EGDe

Based on transcriptional analysis, we decided to perform additional experiments on HPP resistance with the mutants carrying deletions in selected candidate genes. These candidate genes were selected based on the gene-expression data and a potential role in barotolerance and recovery from HPP damage. The selected candidate genes were: *ykuT,* encoding a putative mechanosensitive ion channel of small conductance (OCPFDLNE_01076 and LMOSA_19040 in RO15 and ScottA); and *pbp2A,* encoding for the penicillin-binding protein A2 (OCPFDLNE_02389 and LMOSA_2530 in RO15 and ScottA). For both genes, deletion mutants were generated in the widely used *L. monocytogenes* model strain EGDe (*lmo1013* and *lmo2229* in EGDe, respectively). Successful deletion was validated by genome sequencing (Figure S12). In order to test the resistance of *Listeria monocytogenes* EGDe mutants to HPP, they were grown to stationary growth phase and subjected to HPP at 300 and 350 MPa with a holding time of 5 min. Susceptibility of the mutants to these treatments was assessed by detecting colony forming units. This revealed a reduction of ~ 5 log CFU/mL for Δ*lmo2229* cells and ~ 3 log CFU/mL for both Δ*lmo1013* and wild-type EGDe by the 350 MPa treatment and reduction of ~ 1 log CFU/mL for all three by 300 MPa treatment (Fig. 7). For both 300 MPa and 350 MPa treatments, we observed a significant (*p* < 0.05) difference in colony forming units between wild-type and Δ*lmo2229.* No significant difference was observed between wild-type and Δ*lmo1013.*

## Discussion

High pressure processing (HPP) is commonly used in the food industry to inactivate foodborne pathogens and spoilage microorganisms. However, it has been reported that *L. monocytogenes* is able to recover after HPP treatment during long-term storage [18, 19, 36]. To study gene expression response during early recovery of *L. monocytogenes* and identify genes that are important for recovery, we performed RNA-seq of samples taken at different time points after HPP at two different pressure levels (200 MPa and 400 MPa). To account for strain-dependent variation in the HPP response, experiments were performed with two strains: RO15, a strain that was shown to be more resistant to HPP than others; and ScottA, a strain that is more sensitive to HPP [12]. Both strains are originally isolated from food products [12]. Strain RO15 (clonal complex (CC) 155) belongs to lineage II and serotype 1/2a, while strain ScottA (CC2) belongs to lineage I and serotype 4b [12]. Regarding virulence, strain RO15 shows no intracellular growth in Caco2 cells, but a rather high proliferation rate within macrophages [37]. Compared to Scott A, RO15 has lower invasion efficiency and the intracellular growth coefficient mostly negative, indicating that it is not able to proliferate or survive inside Caco2 cells. Furthermore, RO15 is unable to invade into HEPG2 cells [38].

*L. monocytogenes* can recover within 1 day after injury caused by HPP for 10 min at 450 MPa and 45 °C [19]. Recovery of *L. monocytogenes* was also observed after 6 days of storage at 4 °C for injured bacteria following treatment with HPP at 550 MPa at 45 °C for 10 min [19]. Our results show that viability was unaffected in the two strains after 200 MPa HPP treatment at 8 °C for 8 min (Fig. 1). However, a significant reduction in viable counts was observed for both strains treated at 400 MPa. The reduction in viable counts was significantly higher in ScottA compared to RO15, which supports our previous observation that RO15 is more pressure tolerant [12].

The time-resolved RNA-seq data allowed us to perform gene-network analysis. To summarize the gene networks, we clustered genes assuming that genes within a cluster, and in linked clusters, are functionally related or interact during recovery from HPP [39–41]. HPP mainly affects expression of protein folding genes, PTS system genes, prophage genes, and cobalamin biosynthesis genes. In addition, we observed several hypothetical genes differentially expressed, which can be related to barotolerance in *L. monocytogenes* (Figure S13). We saw that in both strains, stress response genes, virulence genes, and ribosome hibernation promoting factor *hpf* gene were strongly linked to each other, indicating that during recovery from HPP, co-expression of these three factors was needed. It has also been reported that the general stress sigma factor B (σ^B^) regulates *hpf*, *prfA* (encoding listeriolysin regulatory protein), and UspA1,2 (encoding universal stress proteins A1 and A2) [42]. Environmental stress activates σ^B^, which regulates more than 200 genes [42, 43]. In line with this, based on de novo motif discovery analysis, SigB transcription factor binding site-like motif was found in the upstream regions of the gene cluster (Cl6; Fig. 6, Table S14), which includes *prfA* and *hpf* in ScottA. This indicates that a general stress response was activated in *L. monocytogenes* by σ^B^ after HPP.

We have reported that a large portion (up to ~ 85%) of RNA-sequencing reads were mapped to Rli47 ncRNA, which was upregulated in both strains after pressure treatment. Similarly, previous studies have also reported that up to ~ 90% of all RNA-seq reads map to Rli47 ncRNA in *L. monocytogenes* [44, 45]. It has been shown that Rli47 plays a role in the response to acid stress [45] and oxidative stress [46]. In line with these observations, our data suggests that Rli47 is also involved in HPP recovery based on high expression level after HPP treatment. It has also been shown that Rli47 is regulated by σ^B^ [47]. This supports our observation of general stress response activation by σ^B^ after HPP. In addition, Rli53 expression was upregulated in RO15 but not in ScottA. Rli53 has been associated with antibiotic resistance [48]. Our results indicate that Rli53 may also play a role in pressure resistance in RO15.

Cobalamin biosynthesis was the most significantly enriched GO term for upregulated genes in both strains. It has been shown that cobalamin plays a protective role against oxidative stress in bacteria [49]. Cobalamin was also shown to be an essential cofactor for propanediol and ethanolamine utilization [50]. Several studies also observed that cobalamin biosynthesis genes and gene clusters for cobalamin-dependent proteins involved in propanediol and ethanolamine utilization were upregulated under stress conditions in *L. monocytogenes* [44, 51–53], and this was discussed as a strategy to acquire alternative substrates. Interestingly, we observed upregulation of propanediol utilization genes but ethanolamine utilization genes were downregulated after HPP (Figure S3). We speculated that cobalamin biosynthesis and propanediol utilization genes may provide survival advantages to *L. monocytogenes* under HPP stress. In addition, significant downregulation of cobalamin biosynthesis genes in *L. monocytogenes* has been reported in response to Rli47 deletion [47]. Hence, upregulated cobalamin synthesis genes after HPP in this study can be related to increased levels of Rli47, which is regulated by σ^B^. We therefore predicted that cobalamin biosynthesis genes were upregulated as part of the general stress response of HPP.

Stress conditions have been shown to induce prophages in *L. monocytogenes* [54, 55]. Upregulation of prophage genes in both strains after HPP indicates that pressure stress also induces prophages. In addition, co-regulation of different prophages within the same host has also been shown in *L. monocytogenes* [55]. Similarly, our gene-network inference suggests that prophages were linked to each other within strains, coexpression of prophages genes were observed. Based on pan-genome analysis, we previously proposed that prophages and anti-CRISPR genes may play a role in pressure resistance in *L. monocytogenes* [12]. In this study we observed that both anti-CRISPR genes (*acrIIA1* (OCPFDLNE_02770, OCPFDLNE_02583) and *acrIIA2* (OCPFDLNE_02582)) in RO15 were upregulated after HPP. Therefore, it is tempting to speculate that anti-CRISPR genes might provide survival advantages to *L. monocytogenes* by a so far unknown mechanism. RO15 harbours self-targeting spacers in its CRISPR-Cas system. It has been reported that partially matching spacers could have immunopathological effects [56]. One could speculate that the upregulation of the anti-CRISPR genes under stress conditions could be linked to the suppression of the autoimmune activity by the CRISPR-Cas system through self-targeting spacers [57].

GO enrichment analysis of upregulated genes indicated that PTS systems were activated in both strains for most of the time points during the recovery phase. Upregulation of PTS genes has also been reported for other stress conditions in *L. monocytogenes* based on transcriptome analysis [51, 52]. Upregulated PTS systems were mostly galactitol-, fructose-, and mannose-specific PTS systems. These carbon sources play a role in cell-wall biosynthesis [58, 59]. Thus, upregulation of these sugar transporters may be an indication of increased uptake of these sugars for cell-wall biosynthesis and as a carbon source to allow recovery from injury caused by HPP.

CtsR is a negative regulator of heat-shock genes, mainly of the *clp* family of genes, and has been shown to be directly involved in barotolerance of *L. monocytogenes* [60–64]. Deletion of *ctsR* led to an increase in barotolerance of ScottA by 5 orders of magnitude [63]. In addition, upregulation of PTS, heat-shock, and *clp* family genes has been reported for a ScottA *ctsR* mutant [31]. Furthermore, *ctsR* is reportedly regulated by σ^B^ in *Bacillus subtilis* [43]. Our results show upregulation of *ctsR* expression in both strains at some time points after treatment with 200 MPa but not with 400 MPa. Moreover, upregulation of genes was observed for heat-shock proteins of the *clp* family and chaperones in samples treated at both 200 MPa and 400 MPa in both strains (Fig. 3c, 4c). Especially *clpE* was one of the most significantly upregulated genes at several time points. It has been shown that heat-shock proteins are needed to deal with misfolded proteins, prevent cellular damage, and help cell recovery during pressure treatment [65]. Our observation that genes for heat-shock proteins are upregulated indicates a similar role in protection and recovery of *L. monocytogenes* to/after HPP treatment*.*

It has been shown that antibiotic resistant *L. monocytogenes* strains are more resistant to 400 MPa pressure treatment compared to antibiotic-susceptible strains [13]. Our previous pan-genome study [12] also showed that barotolerant strains have slightly different amino acid sequences for *norB* encoding a protein involved in resistance against quinolones. Interestingly, different strains showed variations in their expression of *norB.* Significant upregulation of *norB* was observed in barotolerant RO15 at several time points after 400 MPa treatment, including early time points. However, *norB* was only upregulated at the 24 h time point after 400 MPa treatment in barosensitive ScottA. This supports the observation that differences in antibiotic resistance genes might provide a different barotolerance level within *L. monocytogenes* strains.

Ribosome damage can lead to cell death after HPP [66]. Ribosome hibernation, that is dimerisation of 70S ribosomes leading to translationally inactive 100S particles, has been reported to occur as *L. monocytogenes* adapts to different stress conditions [67]. Ribosome hibernation involves the gene product of *hpf* (ribosome hibernation promoting factor) and upregulation of *hpf* was seen in both strains. Downregulation of *hpf* was observed at the 48 h time point after 200 MPa treatment only in RO15. In addition, in RO15, the GO term “translation” was also mainly enriched for upregulated genes at the late time points in RO15 at both 200 and 400 MPa (Fig. 2). However, no enrichment of GO term “translation” was observed for upregulated genes in ScottA at 400 MPa (Fig. 2). Similarly, several ribosomal and tRNA related genes were upregulated in RO15 but not in ScottA at 400 MPa late time points (Figure S14). Collectively, this indicates that *L. monocytogenes* keeps the translation inactive by inducing Hpf-mediated ribosome hibernation for a certain time after HPP. Moreover, there are differences between the strains in how long this hibernation lasts. The barotolerant strain RO15 seems to reactivate translation faster than the sensitive strain ScottA.

Based on morphological and physiological characterization, the cellular wall or membrane are targets to improve efficacy of HPP to inactivate *L. monocytogenes* [11]. We observed that peptidoglycan-synthesis genes such as, *murG*, *murC*, *murD,* and *pbp2A* were upregulated in both strains after HPP. Upregulation of peptidoglycan-synthesis genes with simultaneous downregulation of cell-division genes indicates that an active cell-wall repair occurs in both strains after HPP. *pbp2A* encodes a penicillin-binding protein that was shown to contribute to β-lactam resistance and cell morphology in *L. monocytogenes* [68]. To further investigate the role of this gene in response to HPP, a mutant carrying a deletion of the corresponding gene (*lmo2229*) was generated in *L. monocytogenes* strain EGDe. It is possible that the product of these genes may have different functionalities in EGDe than in RO15 and ScottA. However, amino acid sequence identity of *lmo2229* and pbp2A of RO15 and ScottA are above 99% (Figure S15) suggesting that they are indeed homologs with identical function. The strain EGDe훥*lmo2229* was tested for resistance against HPP and results show that the *lmo2229* mutant was significantly more sensitive to HPP than the parental wildtype strain (Fig. 7). This supports that *pbp2A* is an important factor in *L. monocytogenes* RO15 and ScottA for recovery after HPP and extends this finding to the widely used model strain EGDe. However, further deletion mutants need to be created in RO15 and ScottA and other strains to dissect the role of *pbp2A* in *L. monocytogenes* especially when exposed to extreme pressure in food matrices.

Peptidoglycan of bacteria consists of a backbone of alternating N-acetylglucosamine (GlcNAc) and N-acetylmuramic acid (MurNAc) units interconnected with peptide side-chains [69]. It has been shown that bacteria are able to recycle N-Acetylglucosamine from peptidoglycan using the proteins encoded by *nagA* and *nagB* [58, 70]. The genomes of *L. monocytogenes* RO15 and ScottA each contain two copies of *nagA* and *nagB,* respectively, and one of the copies of each *nagA* and *nagB* are organized in an operon. Expression of these *nagAB* copies were upregulated in both strains after HPP at 200 and 400 MPa. The second copy of *nagA* was not differentially expressed in either strains. Interestingly, the second copy of *nagB* gene (OCPFDLNE_02454) was only upregulated in RO15. This difference might partly explain the barotolerance difference between strains. Increasing protein levels of the NagB are associated with increased growth rate in *E. coli* [71]. Thus, more efficient biosynthesis of cell-wall peptidoglycans due to higher *NagB* levels may contribute to the higher barotolerance of RO15.

HPP creates a mechanical force that may result in deformation of the membrane. Mechanosensitive channels were shown to respond to membrane stress and help bacteria to cope with this stress [72]. We were intrigued by the observation that the *mscL* gene encoding a MS channel protein of large conductance was upregulated after 400 MPa pressure treatment in both strains. In addition, *ykuT* (encoding small MS channel protein) was upregulated at the 200 MPa 48 h time-point in RO15. However, the obtained *lmo1013* mutant showed no significant difference in susceptibility/resistance to HPP indicating that the small MS channel protein was not directly involved in pressure resistance or only has a minor effect. Further studies that use deletion mutants in RO15, ScottA and possible other existing strains are needed to resolve the functionality of *ykuT* in *L. monocytogenes*.

Gene expression profiling under pressure treatment in *Listeria* was studied previously by Bowman et al. [32] using *L. monocytogenes* strain S2542. Notably, a significant negative correlation was observed for log_2_ FC results between the study by Bowman et al. [32] and our study. *L. monocytogenes* strain S2542 was reported as serotype 1/2a [32], which is the same as strain RO15. Our RNA-seq results were validated using ddPCR (Figure S9, S10, S11) and they are consistent for two different strains under different pressure levels and several time points. We speculate that discordance between the results could potentially arise from different growth conditions, different methods for measuring gene-expression levels (microarray vs RNA-seq), different strain or different treatment time and temperature.

In our previous pan-genome study, we predicted that certain phage genes might be related to barotolerance since they are only detected in barotolerant strains [12]. In the same study we also provided a basic view of transcriptional activity of strain RO15 and ScottA using RNA-seq, which showed upregulation of heat-shock genes under HPP [12] similar to this study. Previously, only simple analysis was able to be performed since the RNA-seq data was limited and only one time point was provided [12]. In contradistinction to previous study, here we performed detailed RNA-seq by focusing recovery of *L. monocytogenes* after HPP by using several time points. We discussed gene expression differences between barotolerant strain RO15 and barosensitive strain ScottA. It is possible that barotolerant strain uses antibiotic resistance and biosynthesis of cell-wall peptidoglycans genes (such as *NagB*) more efficiently to protect itself from pressure stress. Even the general gene expression response looks similar for both strains (Figure S16), there were several more genes expressed differently between studied strains, which might be the reason of barotolerance (Figure S16).

Overall, these findings may lead to new approaches to improve HPP efficacy. For example, we observed that the mannose phosphotransferase system (Man-PTS) was upregulated after HPP treatment. Man-PTS is the receptor for class IIa bacteriocins, such as pediocin or garvicin [73–75]. Thus, increased expression of these receptors may provide an opportunity to pre-treat food with IIa bacteriocins, which may increase susceptibility to HPP. However, *dltD* upregulation in RO15 may lead to incorporation of more alanine residues [76], which increases the positive charge and consequently reduces affinity to cationic antimicrobials and bacteriocins. Interestingly, *dltD* was downregulated in ScottA indicating pre-treatment might be more effective for barosensitive strains. In addition, among peptidoglycan biosynthesis genes, deletion of *pbp2A* causes significant susceptibility to HPP. Hence new approaches could be sought by using peptidoglycan cross-linking.

## Conclusion

Recovery and outgrowth of *L. monocytogenes* in food after HPP treatment is a serious problem for the food industry. The mechanism of recovery of *L. monocytogenes* after HPP has not been studied by genome-wide transcriptional profiling. Understanding how bacteria recover from HPP injury may help the food industry to develop new strategies for better inactivation of food pathogens. Here we reported a very detailed gene expression response of *L. monocytogenes* during recovery from HPP treatment using two strains (barotolerant and barosensitive), several time points, and two different pressure levels. Protein folding, PTS system, universal stress response, and cobalamin biosynthesis were the main activated functions in response to HPP treatment in *L. monocytogenes*. We showed that ncRNAs may also play a role in HPP injury recovery. Based on our results, several genes involved in barotolerance and recovery from HPP injury were predicted. Deletion of *pbp2A* suggests that it plays a role in barotolerance of *L. monocytogenes*. Further reverse-genetics experiments are required to validate our predictions based on RNA-seq.

## Methods

### High pressure processing for inactivation of strain RO15 and ScottA

Pressure treatment testing the log reduction was carried out using the QFP 2 L-700 (Avure Technologies Inc., Columbus, USA) as previously described [12], except that a holding time of 8 min, vessel water temp of 8 °C, and pressures of 200 and 400 MPa were used for strain RO15 (isolated as described previously [12]) and ScottA (CIP103575, obtained from Centre de Ressources Biologiques de l’Institut Pasteur, Paris, France). The compression rate during pressure build up was 50 s for 200 MPa and 85 s for 400 MPa. The pressure release was immediate. The duration of treatment did not include the come up time. Adiabatic heating caused ~ 6 °C and ~ 12 °C temperature increases for 200 and 400 MPa, respectively. Immediately after pressure treatment, pressurized and untreated samples were serially ten-fold diluted in tryptic soy broth with 0.6% yeast extract (TSBYE; Oxoid, Basingstoke, Hampshire, England) and plated in triplicate on tryptic soy agar with 0.6% yeast extract (TSAYE; Oxoid, Basingstoke, Hampshire, England) by using a spiral plater (Eddy Jet; IUL Instruments, Barcelona, Spain). Pressurized samples at 400 MPa were additionally plated manually (100 uL) without being diluted. TSAYE plates were incubated at 37 °C for 48 h prior to counting the colonies and estimating bacterial inactivation.

### High pressure processing for RNA-seq

Volumes of 350 mL BHI broth (Oxoid, Basingstoke Hampshire, England) were inoculated with *L. monocytogenes* RO15 or Scott A (CIP103575, obtained from Centre de Ressources Biologiques de l’Institut Pasteur, Paris, France), respectively, from fresh over-night cultures grown in the same medium at 37 °C to an OD_600_ of ~ 0.1. The precultures’ volumes necessary to adjust the Scott A and RO15 cultures to 0.1 OD_600_ were 34 and 25 mL, respectively. The cultures were grown at 37 °C to early stationary phase (~ 1.3 OD_600_) transferred into 2 mL tubes, and cooled at 4 °C for 1 h. The samples were then treated at 200 and 400 MPa, 8 °C, for 8 min, in multi-vessel high-pressure equipment (Resato, Roden, the Netherlands), using a mixture of water and propylene glycol as transmitting fluid (TR15, Resato). Due to adiabatic heating, liquid temperatures inside the vessel after pressure build-up increased to 16 °C after the 200 MPa treatment, and to 23 °C after the 400 MPa treatment. The compression rate during pressure build up was 100 MPa/min and an extra 1 min was considered as equilibration time. The decompression of the vessels took approximately 5 s. After HPP, the samples were maintained at 8 °C for recovery, as follows: 0 min (T1), 5 min (T2), 10 min (T3), 30 min (T4), 45 min (T5), 60 min (T6), 6 h (T7), 24 h (T8), and 48 h (T9). Control samples were held at 8 °C at atmospheric pressure (Figure S17). In order to stabilize the RNA, both treated samples (5 replicates) and corresponding controls (4 replicates) were transferred in 4 mL of RNA protect reagent (Qiagen, Hilden, Germany), incubated at room temperature for 5 min, pelleted by centrifugation at 5000 rpm and stored at − 80 °C, until RNA was extracted.

### RNA extraction

RNA was extracted from samples obtained from the barotolerance experiment (*n* = 320) with NucleoSpin RNA kit (Macherey-Nagel, Düren, Germany) as described previously [12]. BioAnalyzer RIN values were checked after RNA extraction to evaluate the integrity of the isolated RNA. The average of the RIN value was 9.97 with a range of 8 to 10 across all the samples.

### RNA-sequencing

RNA-sequencing was performed for 216 samples from the barotolerance experiment including three replicate samples for each treatment and time point (Table S16). Prior to RNA-seq library preparation, rRNAs were removed by hybridizing extracted RNA with DNA oligos complementary to 16S, 23S, and 5S rRNAs followed by digestion of resulting DNA-RNA hybrid molecules. Hybridization reactions included 1 μg RNA, 1 x buffer (16.7 mM Tris-HCl, pH 8, 33.3 mM KCl), and 250 nM pooled DNA oligos in a total volume of 12 μl. Reactions were incubated at 95 °C for 2 min, slowly (0.1 °C/sec) cooled to 22 °C, incubated at 22 °C for a further 5 min, and placed on ice. For digestion of rRNAs, 1.5 μl of 10 x RNaseH buffer, 0.2 μl RNAseH (Thermo Scientific, Waltham, Massachusetts, United States), 0.5 μl Ribolock RNase inhibitor (Thermo Scientific), and 0.8 μl of water were added. Digestion reactions were incubated at 37 °C for 60 min, and inactivated at 65 °C for 20 min. Remaining unhybridized DNA oligos were removed with RapidOut DNA Removal kit (Thermo Scientific) according to manufacturer’s instructions. RNA-seq libraries were prepared using QIAseq stranded Total RNA Lib kit (Qiagen) using $$ \raisebox{1ex}{$1$}\!\left/ \!\raisebox{-1ex}{$3$}\right. $$ reaction volumes. Due to the large number of samples, purification steps were performed with a Magnatrix1200 pipetting robot (Magnetic Biosolutions) using precipitation on Dynabeads MyOne Carboxylic acid beads (Invitrogen, Carlsbad, California, United States), and 10% PEG after reverse transcriptase and second strand synthesis steps, and 9.5% PEG after the strand-specific ligation step. Instead of the kit’s adapter, a truncated TruSeq adapter was used in the strand-specific ligation. Libraries were amplified using half of the purified ligation product (10 μl) in 1 x HF buffer with 0.2 mM dNTPs, 0.6 μM dual-index primer [77], and one unit of Phusion Hot Start II High-Fidelity DNA polymerase (Thermo Fisher Scientific) in a total volume of 50 μl. The PCR protocol that was used was 98 °C, 30 s; 20 x (98 °C, 10 s; 65 °C, 30 s; 72 °C, 10 s); 72 °C, 5 min. Concentrations of amplified libraries were measured with a Qubit fluorometer and dsDNA HS assay kit (Invitrogen, Carlsbad, California, United States), and size distributions visualized with Fragment Analyzer and High Sensitivity NGS Fragment Analysis kit (Advanced Analytical, Parkersburg, WV, USA). Amplified libraries were pooled into two batches, each including 108 samples. The first pool was concentrated using an Amicon Ultra 100 K column (Millipore, Burlington, MA, USA), purified once with 0.9 x AMPure XP beads (Beckman Coulter, Brea, CA, USA), and once with PEG (8–8.5%)/NaCl precipitation on Dynabeads MyOne™ carboxylic beads (Invitrogen). The second pool was purified twice with 0.9 x AMPure XP beads (Beckman Coulter). For both pools, size selection of 300–600 bp fragments was performed using BluePippin and 2% agarose gel cassette (Sage Science). Finally, the pools were purified with S-400 Microspin HR columns (GE Healthcare, Chicago, Illinois, United States). NextSeq 500 (Illumina, San Diego, CA, USA) was used to sequence the RNA-seq libraries twice. Altogether, seven sequencing runs were performed to produce 76 bp single-end reads.

### RNA-seq data pre-processing and differential expression analysis

RNA-seq reads obtained from all samples were processed using Trimmomatic v0.36 [78] to trim off the low-quality bases and filter out the adapter sequences. SortmeRNA v2.1b [79] was used to filter out ribosomal RNA reads. Reads were then mapped against the corresponding genomes (ScottA: GCA_000212455.1, RO15: GCA_902827145.1) using bowtie2 v2.3.4.3 [80] with default settings. Counting of reads per gene was performed using HTSeq v0.9.1 [81] with union mode. Hierarchical clustering of samples (HCA) based on Euclidean distances and principal-component analysis (PCA) of the samples was done for each *L. monocytogenes* strain as described in the manual for the DESeq2 R package v1.22.2 [82] on “regularized log” (rlog)-transformed read count data to visually explore sample relationships. One sample in ScottA (R_046) and two samples in RO15 (R_045, R_055) belonged neither to control nor to HPP clusters and were thus discarded as potential outliers. In addition, two RO15 samples (R_057, R_235) contained a very low number of CDS-mapped reads (approx. 0.05 million) and were also discarded (Table S17).

Differential expression analysis for each *L. monocytogenes* strain was performed using R package DESeq2 v1.22.2 [82]. The comparisons were made in multiple ways: 1) Comparing the HPP-treated samples to the corresponding controls for each time point and at different HPP level, separately; 2) Comparing the time dynamics of gene expression (as described in DESeq2 manual) between control and treated samples, separately for 200 and 400 MPa series, to find the genes that changed expression at least at one time point; the gene expression at time point 0 in control was taken as a proxy for gene expression before the HPP treatment (designated as T00). We used Benjamini-Hochberg procedure for correction of multiple testing during differential gene expression analysis. Genes were considered to be significantly differentially expressed if their adjusted *p*-value ≤0.05 and their log_2_ fold change (log_2_ FC) ≥ 0.6, (therefore, fold change FC ≥ 1.5). For the time dynamic comparison, genes were considered to be significantly differentially expressed at least at one time point if the adjusted *p*-value ≤0.05.

### Gene regulatory network construction, clustering and visualization

The initial set of genes used to build the network contained 1964 genes for strain RO15 and 1852 genes for ScottA that changed expression between the corresponding controls and treatments at least at one time point (adjusted p-value < 0.05). The gene expression values used to build a network were regularized log (rlog) transformed as described in DESeq2 [82] to stabilize the variance and normalize the count data. Eleven networks have been built using 11 publicly available algorithms (clr, genie3, aracne, pearson_corr, narromi, pcor, plsnet, tigress, llr-ensemble, el-ensemble) embedded into Seidr toolkit [83] and integrated into one network using Seidr. The hard threshold for the edge score was manually chosen to be 0.4 based on the criteria described here (https://seidr.readthedocs.io/en/latest/source/threshold/threshold.html) leaving only 1506 genes, 3661 edges for strain RO15, and 1389 genes, 3427 edges for strain ScottA in the final weighted undirected network. Infomap v0.19.26 [34] two-level clustering with options “--bftree -2 --flow-network -N 10000” was used to find clusters (modules) of genes in the network and the flow between the modules (the strength of interactions between the modules). The two-level representation was visualized using the map&alluvial generator (http://www.mapequation.org/apps/MapGenerator.html). The network was visualized and centrality metrics calculated for the nodes (e.g. Degree, Betweenness and Closeness) using Cytoscape [84].

MEME suite v5.0.5 [85] was used for motif analysis. The upstream regions of genes ranging from 50 to 300 nucleotides were extracted using python script (https://github.com/peterthorpe5/intergenic_regions). De novo motif discovery was performed using MEME [85]. The discovered motifs were searched against transcription-factor binding-site databases, such as CollecTF [86], PRODORIC [35], RegTransBase [87], RegPrecise [88], DPINTERACT [89], and Swiss Regulon [90], using Tomtom tool [91]. The listed databases were downloaded from the MEME suite as meme database format, except RegPrecise. For RegPrecise, all transcription factor binding site motifs for *Listeriaceae* were downloaded and converted to the meme database using sites2meme script from the MEME suite v5.0.5 [85].

### Functional enrichment analyses of the DE gene lists

GO terms of the genes have been predicted using PANNZER2 [92] with default parameters. The lists of DE genes obtained from the different comparisons and the network gene clusters were tested for the enrichment of GO terms (belonging to the Biological Process ontology) using topGO package [93]. Enrichment for GO terms was tested separately for up- and downregulated genes. The reference set included all the GO term-annotated genes in the genome. The functional categories were considered to be enriched if p-value ≤0.05. KAAS (KEGG Automatic Annotation Server) [94] was also used to obtain KO (KEGG Orthology) assignments of genes.

### ddPCR

ddPCR was used to verify RNA-seq results of the barotolerance experiment (*n* = 18). Three replicate samples of each treatment or strain and their corresponding control samples were always analysed. Expression levels of ten genes: *recG*, *fusA, clpE, hly, agrB, ftsE, mscL, pflA, dnaK*, and *murA*, were quantified from ScottA samples treated with 200 MPa and recovered for 24 h, or treated with 400 MPa and recovered for 10 min. Expression levels of seven genes: *recG, fusA, clpE, hly, agrB, ftsE*, and *mscL* were quantified using ScottA samples treated with 400 MPa and recovered for 24 h. Primers (Table S15) were designed using Primer3Plus [95] and manufactured by Integrated DNA Technologies. The protocol included gDNA removal and RT-PCR steps as described previously [96]. To be able to compare expression levels of different samples, expression of the target genes (cDNA copies/μl) was normalized using concentrations of two stably expressed genes: *recG* and *fusA*. To help comparison to RNA-seq, the results were expressed as log_2_ (gene concentration in treated sample/gene concentration in control sample) values.

### Deletion of *lmo1013* and *lmo2229* genes from *L. monocytogenes* EGDe genome

The bacterial strains and plasmids used in the present study are listed in Table 1. Culture media utilized for the cultivation of *E. coli* and *L. monocytogenes* were Luria-Bertani (LB) broth (Sigma Aldrich, St. Louis, Missouri, United States) and brain heart infusion (BHI) broth (Oxoid), respectively. *E. coli* EC10B chemically competent cells were prepared with the CaCl_2_ method [101] and *L. monocytogenes* EGDe electrocompetent cells were obtained as described earlier [99]. The selective antibiotics and their concentrations were used as follows: kanamycin, 50 μg/mL for *E. coli*; erythromycin, 250 μg/mL for *E. coli* and 5 μg/mL for *L. monocytogenes*; chloramphenicol, 7.5 μg/ml for *L. monocytogenes.*

**Table 1:** Strains and plasmids used in this study

Plasmids and strains	Characteristics	Source/ Reference
**Plasmids**		
*p*ORI280	RepA− gene replacement vector, constitutive *lacZ*, 5.3 kb, Em^r^	[97]
*p*ORI280ADΔ*lmo1013*	*p*ORI280 containing 503-bp region on either side flanking *lmo1013* deletion *p*ORI280 including both upstream and downstream flanking regions (503 bp each) of *lmo1013* deletion	This study
*p*ORI280ADΔ*lmo2229*	*p*ORI280 containing 503-bp region on either side flanking *lmo2229* deletion *p*ORI280 including both upstream and downstream flanking regions (503 bp each) of *lmo2229* deletion	This study
*p*VE6007	Temperature-sensitive helper plasmid that provides RepA in *trans*, Cm^r^	[98]
***E. coli*** **strains**		
EC10B	DH10B derivative with Kan^r^ and RepA integrated in the *glgB* gene	[99]
EC10B/*p*ORI280ADΔ*lmo1013*	E. coli EC10B with *p*ORI280ADΔ*lmo1013*	This study
EC10B/pORI280ADΔ*lmo2229*	E. coli EC10B with *p*ORI280ADΔ*lmo2229*	This study
***L. monocytogenes*** **strains**		
EGDe	Serotype 1/2a	[99, 100]
EGDeΔ*lmo1013*	EGDe with the entire *lmo1013* gene deleted	This study
EGDeΔ*lmo2229*	EGDe with the entire *lmo2229* gene deleted	This study

Two *L. monocytogenes* EGDe mutants were generated by chromosomal mutagenesis of *lmo1013* and *lmo2229* genes, based on the system composed of *p*ORI280AD recombinant vector and *p*VE6007 helper plasmid, following the protocol provided by Monk et al. [99].

The oligonucleotide primers used to amplify the flanking regions that shared 20 bp overlapping ends with *Pst*I linearized *p*ORI280 vector (Thermo Scientific) and 20 bp overlapping ends between them are presented in Table 2.

**Table 2:** Primers used in the amplification of AB and CD fragments from *L. monocytogenes* EGDe genomic DNA

Primers	Sequence 5′- 3′	Amplimer obtained
*lmo*1013_AB_fwd	TCGAATTCGAAGCTTCTGCATAACACCAATAGTCGCCCCT	Upstream flanking fragment (AB) of *lmo1013* coding region including the start codon (ATG)
*lmo*1013_AB_rev	AGCGAATTGGCGTCTTTTTACATTTTTTGGTCCACATCCT	
*lmo*1013_CD_fwd	AGGATGTGGACCAAAAAATGTAAAAAGACGCCAATTCGCT	Downstream flanking fragment (CD) of the *lmo1013* coding region including the stop codon (TAA)
*lmo*1013_CD_rev	ATGACGTCGACGCGTCTGCATAGTGCAGTTATTACGATTG	
*lmo*2229_AB_fwd	TCGAATTCGAAGCTTCTGCATCAGGTGGCTCGATTGCAAA	Upstream flanking fragment (AB) of the *lmo2229* coding region including the start codon (ATG)
*lmo*2229_AB_rev	GTAAAATGTGCTTTTAATTACATGTAACTCTCCTATCTTC	
*lmo*2229_CD_fwd	GAAGATAGGAGAGTTACATGTAATTAAAAGCACATTTTAC	Downstream flanking fragment (CD) of the *lmo2229* coding region including the stop codon (TAA)
*lmo*2229_CD_rev	ATGACGTCGACGCGTCTGCATTCACCATCTAAAGTAATTT	

AB and CD fragments were generated by high-fidelity PCR amplification from *L. monocytogenes* EGDe genomic DNA isolated with Jena Bioscience kit (Jena, Germany) according to the manufacturer instructions. The reaction mixture (25 μL) was prepared in accordance with the indications of Phusion High-Fidelity DNA (Thermo Scientific) manufacturer, including 3% DMSO, 0.5 μM of each primer and 200 μM of each deoxynucleoside triphosphate. The PCR reaction conditions consisted of an initial denaturation step at 98 °C for 30 s followed by 30 cycles of 98 °C for 10 s, 60 °C for 30 s, and 72 °C for 30 s/kb and then a final extension at 72 °C for 5 min.

After gel extraction and purification (FavorPrep™ GEL/PCR Purification Kit, Favorgen), the gene deletion flanking fragments were ligated into the cut *p*ORI280 backbone by Gibson assembly (2X Gibson Assembly® Ultra Master Mix, Synthetic Genomics Inc) reaction (*p*ORI280AD). Following chemically competent *E. coli* EC10B cells’ transformation with the recombinant vector by heat shock, colony PCR was employed to screen for transformants. This was done by AD fragment amplification from the total DNA released from the heat treated cells (94 °C, 15 min.) with KAPA Taq DNA Polymerase (Sigma Aldrich) and primers pairs *lmo1013*_AB_fwd / *lmo1013*_CD_rev and *lmo2229*_AB_fwd / *lmo2229*_CD_rev.

Further, the electroporation of *L. monocytogenes* EGDe and the site-directed mutagenesis of *lmo1013* and *lmo2229* genes were performed according to the protocol of Monk et al. [99]. Gene deletion screening was performed by Colony PCR using the primers pairs AB_fwd/CD_rev to amplify the genomic region that encompasses both flanking fragments and gene of interest.

Gene deletion from *L. monocytogenes EGDe* genome was further confirmed by DNA sequencing. The same methods were used for genome sequencing and assembly of wild- type EGDe, Δ*lmo1013*, and Δ*lmo2229* as described previously [12]. In addition, the reads were mapped to reference *L. monocytogenes* EGDe genome and using Bowtie2 v2.3.4.3 [80] and visualized using IGV v2.4.19 [102] to focus on deletion regions.

### Resistance of *L. monocytogenes* EGDe mutants to HPP

The *L. monocytogenes EGDe* strains were grown in TSB with 0.6% yeast extract (TSB-YE) to early stationary phase, prepared as described previously in High pressure processing for RNA-seq method and then subjected to HPP at 300 and 350 MPa, 8 °C, for 5 min. Due to adiabatic heating, water temperatures in the middle of the vessel at the end of pressure treatment had risen to 23.28 °C after the 300 MPa treatment, and 23.88 °C after the 350 MPa treatment. Lower pressures than those chosen in the recovery experiments were selected to demonstrate if increased susceptibility of the mutants to HPP was acquired. Both control and treated samples were serially diluted 1:10 in PBS, plated on TSA with 0.6% yeast extract (TSA-YE) and incubated at 37 °C for 48 h.

## Supplementary Information

**Additional file 1: Figure S1.** PCA plot for RNA-seq count data. Figure shows the PCA plot for RNA-seq count data for a) strain RO15 all samples, b) strain ScottA all samples, c) RO15 200 MPa samples, d) RO15 400 MPa samples, e) ScottA 200 MPa samples, f) ScottA 200 MPa samples. For both strains we can see a clear separation between treatment and control samples. Each circle represents one sample. Colour representations are given on the right side of the figure.

**Additional file 2: Figure S2**. Number of significantly differentially expressed genes. Figure shows number of differentially expressed genes after pressure treatment for each time point for (a) strain RO15 and (b) strain ScottA. Red bar represents the number of upregulated genes, and the blue bar represents the number of downregulated genes.

**Additional file 3: Figure S3**. The log_2_ fold change heatmap of propanediol utilization, ethanolamine utilization, and cobalamin biosynthesis genes. A) log_2_ fold change values for 200 MPa treatment, b) log2 fold change values for 400 MPa treatment. The gene name and locus tag of genes for RO15 and ScottA were given at the end of the row. The log_2_ fold change scale is shown at the right corner. In addition, gene homology of cobalamin biosynthesis is shown on the second page.

**Additional file 4: Figure S4**. The log_2_ fold change heatmap of transcription factor genes. a) RO15 genes, b) ScottA genes, c) ortholog genes for 200 MPa treatment, d) ortholog genes for 400 MPa treatment. The gene name and locus tag of genes for RO15 and ScottA are given at the end of the row. The log_2_ fold change scale is shown at the right corner.

**Additional file 5: Figure S5**. The log_2_ fold change heatmap of transcription machinery genes. a) RO15 genes, b) ScottA genes, c) ortholog genes for 200 MPa treatment, d) ortholog genes for 400 MPa treatment. The gene name and locus tag of genes for RO15 and ScottA are given at the end of the row. The log_2_ fold change scale is shown at the right corner.

**Additional file 6: Figure S6.** The log_2_ fold change heatmap of genes of RO15 that is not found in ScottA. Locus tag of genes are given at the end of the row. Phage genes were indicated with “phage” text. The log_2_ fold change scale is shown at the right corner.

**Additional file 7: Figure S7**. The log2 fold change heatmap of genes of ScottA that is not found in RO15. Locus tag of genes were given at the end of the row. Phage genes were indicated with “phage” text. The log_2_ fold change scale is shown at the right corner.

**Additional file 8: Figure S8**. The log_2_ fold change heatmap of ncRNA genes. a) log_2_ fold change values for 200 MPa treatment, b) log_2_ fold change values for 400 MPa treatment. The gene name and locus tag of genes for RO15 and ScottA were given at the end of the row. The log_2_ fold change scale is shown at the right corner.

**Additional file 9: Figure S9.** Figure ddPCR / RNA-seq correlation for log2 fold change. The figure shows log2 fold-changes observed by ddPCR plotted against RNA-seq data for samples of ScottA obtained 24 h after treatment with 200 MPa. Transcripts levels of individual genes obtained by ddPCR were normalized to recG levels. Blue circles represent genes that were significantly differentially expressed in both ddPCR and RNA-seq results. White circles represent genes that were not significantly differentially expressed in both ddPCR and RNA-seq. The orange and blue gene names indicate significant up- and down regulation, respectively. The Pearson correlation coefficient between ddPCR and RNA-seq log2 fold change results was 0.99 (r=0.99).

**Additional file 10: Figure S10.** ddPCR / RNA-seq correlation for log_2_ fold change. The figure shows log_2_ fold-changes observed by ddPCR plotted against RNA-seq data for samples of ScottA obtained 10 min after treatment with 400 MPa. Transcripts levels of individual genes obtained by ddPCR were normalized to *recG* levels. Blue circles represent genes that were significantly differentially expressed in both ddPCR and RNA-seq results. White circles represent genes that were not significantly differentially expressed in both ddPCR and RNA-seq. The orange and blue gene names indicate significant up- and down regulation, respectively. The Pearson correlation coefficient between ddPCR and RNA-seq log_2_ fold change results was 0.97 (r=0.97).

**Additional file 11: Figure S11**. ddPCR / RNA-seq correlation for log_2_ fold change. The figure shows log_2_ fold-changes observed by ddPCR plotted against RNA-seq data for samples of ScottA obtained 24 h after treatment with 400 MPa. Transcript levels of individual genes obtained by ddPCR were normalized to *recG* levels. Blue circles represent genes that were significantly differentially expressed in both ddPCR and RNA-seq results. White circles represent genes that were not significantly differentially expressed in both ddPCR and RNA-seq. The orange and blue gene names indicate significant up- and down regulation, respectively. The Pearson correlation coefficient between ddPCR and RNA-seq log_2_ fold change results was 0.97 (r=0.97).

**Additional file 12: Figure S12.** Visualization of DNA sequencing reads for strain EGDe, Δ*lmo1013*, and Δ*lmo2229*. a) focused the region includes the gene *lmo1013*, b) focused the region includes the gene *lmo2229*.

**Additional file 13: Figure S13.** Log_2_ fold change heatmap of hypothetical/uncharacterized genes of RO15 and ScottA.

**Additional file 14: Figure S14.** Log_2_ fold change heatmap of elongation factor, ribosomal protein, and tRNA genes at 400 MPa.

**Additional file 15: Figure S15.** Visualization of amino acid sequence similarity of *lmo1013* and *lmo2229* genes in strain EGDe, RO15, and ScottA.

**Additional file 16: Figure S16.** Log_2_ fold change heatmap of all differentially expressed genes in both strains. The rows were clustered based on log_2_ fold change. Clusters that show strain-specific differences were shown in detail.

**Additional file 17: Figure S17.** Visualization of the experimental design.

**Additional file 18: Table S1.** List of up- and downregulated genes after 200 MPa treatment in RO15.

**Additional file 19: Table S2.** List of up- and downregulated genes after 400 MPa treatment in RO15.

**Additional file 20: Table S3.** List of up- and downregulated genes after 200 MPa treatment in ScottA.

**Additional file 21: Table S4.** List of up- and downregulated genes after 400 MPa treatment in ScottA.

**Additional file 22: Table S5.** List of enriched GO terms for both up- and downregulated genes after 200 MPa and 400 MPa HPP.

**Additional file 23: Table S6.** 200 MPa and 400 MPa specific upregulated genes in RO15. The table is divided into five main columns. First column shows genes upregulated both at 200 MPa and 400 MPa. Second column shows genes upregulated at 200 MPa but not at 400 MPa. Third column shows genes upregulated at 400 MPa but not at 200 MPa. Fourth column shows GO terms enriched for second column genes. Fifth column shows GO terms enriched for third column genes. Time points were divided with sheets.

**Additional file 24: Table S7.** 200 MPa and 400 MPa specific upregulated genes in ScottA. The table is divided into five main columns. First column shows genes upregulated both at 200 MPa and 400 MPa. Second column shows genes upregulated at 200 MPa but not at 400 MPa. Third column shows genes upregulated at 400 MPa but not at 200 MPa. Fourth column shows GO terms enriched for second column genes. Fifth column shows GO terms enriched for third column genes. Time points were divided with sheets.

**Additional file 25: Table S8.** Annotation of ncRNA genes in RO15 and RNA-seq read counts for them in RO15.

**Additional file 26: Table S9.** Annotation of ncRNA genes in RO15 and RNA-seq read counts for them in ScottA.

**Additional file 27: Table S10.** Table shows gene network link and score of the aggregated network. Undirected links between genes were shown in this table, the third column represents the score of the aggregated network calculated using Seidr. Sheet 1: RO15, Sheet 2: ScottA.

**Additional file 28: Table S11.** List of clustered genes and cluster links in RO15.

**Additional file 29: Table S12.** List of clustered genes and cluster links in ScottA.

**Additional file 30: Table S13.** Significant de novo predicted binding motif from upstream regions of clustered genes in RO15. Predicted motifs and upstream region sites were listed. If a database match was seen for the predicted motif, tomtom database results were also listed after the motifs.

**Additional file 31: Table S14.** Significant de novo predicted binding motif from upstream regions of clustered genes in ScottA. Predicted motifs and upstream region sites were listed. If a database match was seen for the predicted motif, tomtom database results were also listed after the motifs.

**Additional file 32: Table S15.** Primers used in ddPCR experiments.

**Additional file 33: Table S16.** Sample IDs used for RNA-seq. Table shows sample IDs and related experimental conditions for both RO15 and ScottA.

**Additional file 34: Table S17.** Raw CDS counts. Table shows raw RNA-seq read counts in CDS in both RO15 and ScottA.

## Data Availability

All sequencing data have been deposited in the European Nucleotide Archive (ENA) under accession code PRJEB34771 (www.ebi.ac.uk/ena/browser/view/PRJEB34771). We used publicly available genome assemblies of *L. monocytogenes* strain ScottA (https://www.ncbi.nlm.nih.gov/assembly/GCF_000212455.1/), strain RO15 (https://www.ncbi.nlm.nih.gov/assembly/GCF_902827145.1), and strain EGDe (https://www.ncbi.nlm.nih.gov/assembly/GCF_000196035.1) from NCBI database. CollecTF, PRODORIC, RegTransBase, DPINTERACT, and Swiss Regulon databases were downloaded from http://meme-suite.org/db/motifs. RegPrecise *Listeriaceae* transcription factor motifs were downloaded from https://regprecise.lbl.gov/collection_tax.jsp?collection_id=1006.

## Abbreviations

ncRNA: Non-coding RNA
CDS: Coding sequence
cfu: Colony forming units
HPP: High pressure processing
MPa: Megapascals
