# Supplementary material for: High-pressure processing-induced transcriptome response during recovery of Listeria monocytogenes
Source: BMC Genomics. 2021 Feb 12;22:117. doi: 10.1186/s12864-021-07407-6 (PMC7881616; doi:10.1186/s12864-021-07407-6)
Supplement: Supplementary file 1 — Additional file 1: Figure S1. PCA plot for RNA-seq count data. Figure shows the PCA plot for RNA-seq count data for a) strain RO15 all samples, b) strain ScottA all samples, c) RO15 200 MPa samples, d) RO15 400 MPa samples, e) ScottA 200 MPa samples, f) ScottA 200 MPa samples. For both strains we can see a clear separation between treatment and control samples. Each circle represents one sample. Colour representations are given on the right side of the figure. [file 12864_2021_7407_MOESM1_ESM.pdf]

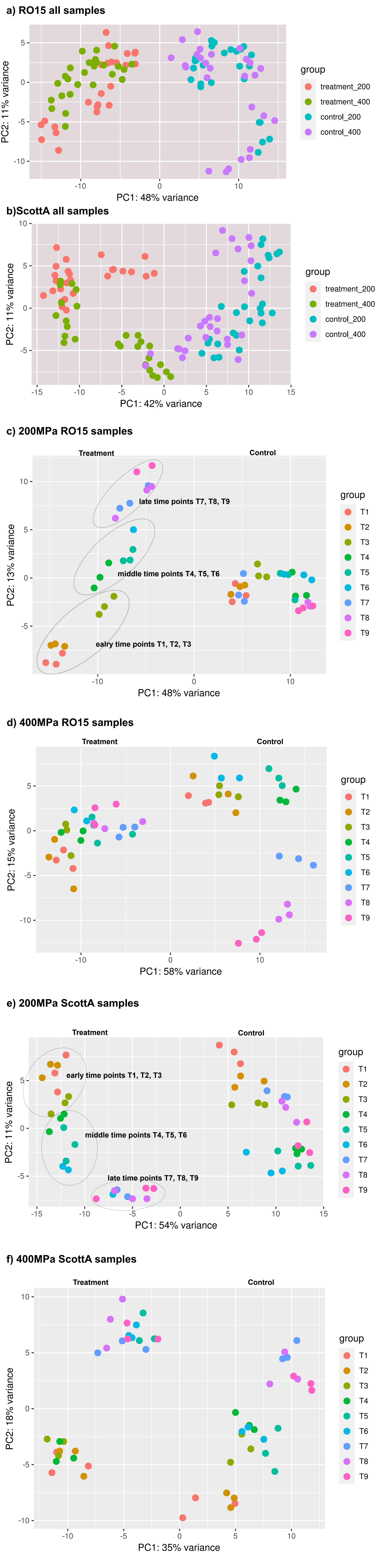
a) RO15 all samples
PC2: 11% variance
PC1: 48% variance
group
treatment_200
treatment_400
control_200
control_400
b)ScottA all samples
PC2: 11% variance
PC1: 42% variance
group
treatment_200
treatment_400
control_200
control_400
c) 200MPa RO15 samples
Treatment
Control
late time points T7, T8, T9
middle time points T4, T5, T6
ealry time points T1, T2, T3
PC2: 13% variance
PC1: 48% variance
group
T1
T2
T3
T4
T5
T6
T7
T8
T9
d) 400MPa RO15 samples
Treatment
Control
PC2: 15% variance
PC1: 58% variance
group
T1
T2
T3
T4
T5
T6
T7
T8
T9
e) 200MPa ScottA samples
Treatment
Control
early time points T1, T2, T3
middle time points T4, T5, T6
late time points T7, T8, T9
PC2: 11% variance
PC1: 54% variance
group
T1
T2
T3
T4
T5
T6
T7
T8
T9
f) 400MPa ScottA samples
Treatment
Control
PC2: 18% variance
PC1: 35% variance
group
T1
T2
T3
T4
T5
T6
T7
T8
T9
