# Supplementary figures and images for: High-pressure processing-induced transcriptome response during recovery of Listeria monocytogenes
Source: BMC Genomics. 2021 Feb 12;22:117. doi: 10.1186/s12864-021-07407-6 (PMC7881616; doi:10.1186/s12864-021-07407-6)

a) RO15

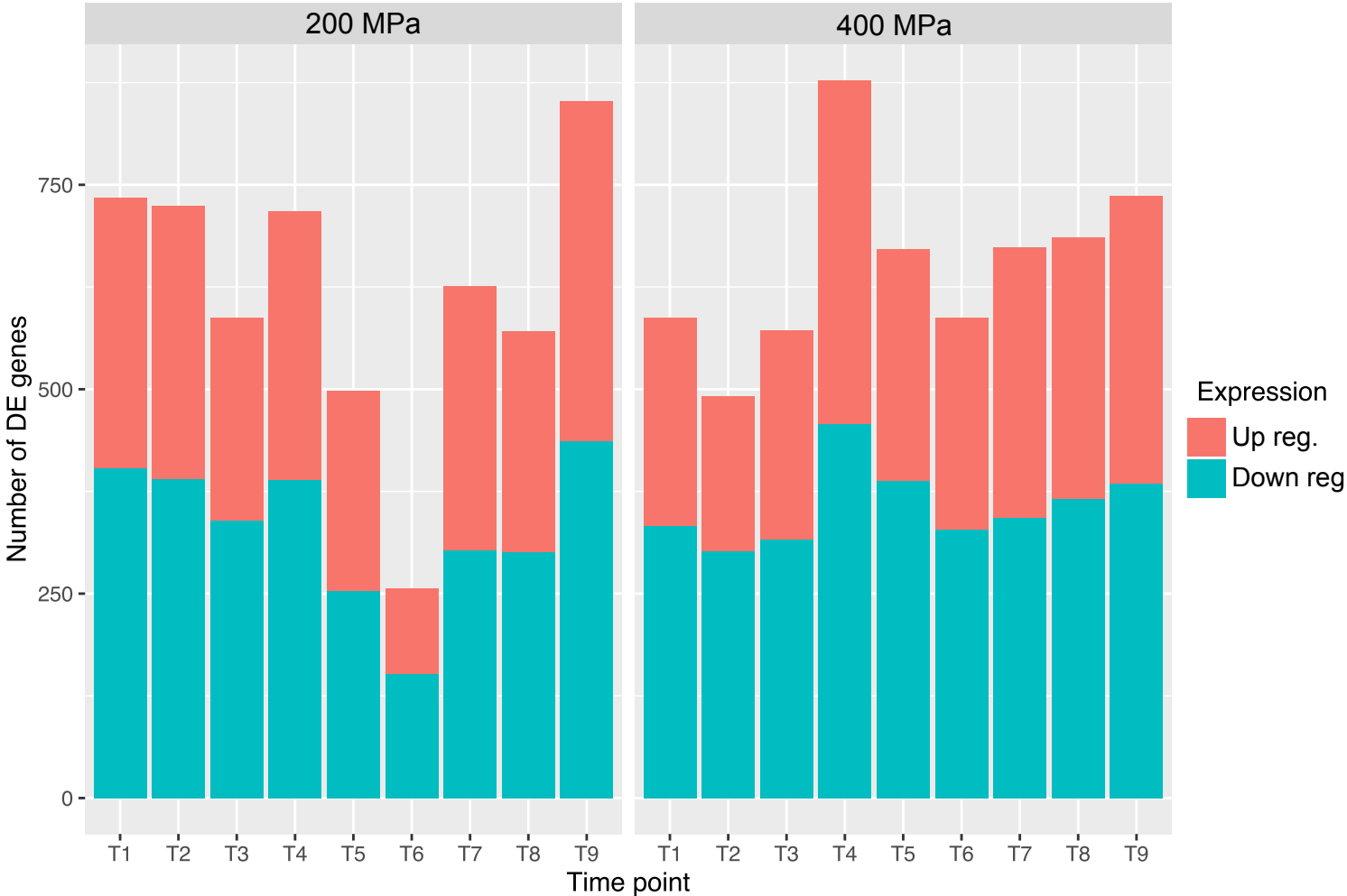

b) ScottA

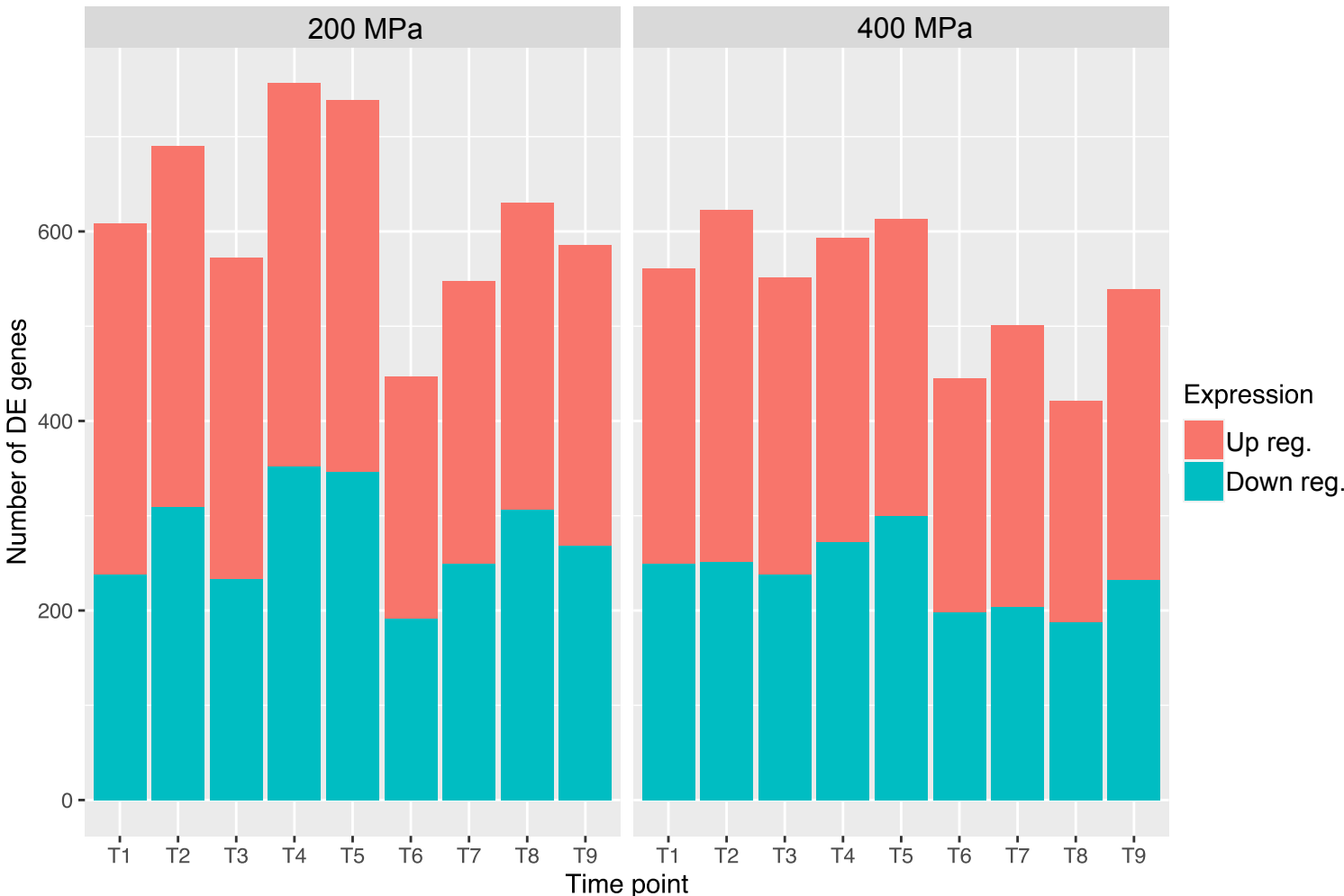

Supplement: Supplementary file 2 — Additional file 2: Figure S2. Number of significantly differentially expressed genes. Figure shows number of differentially expressed genes after pressure treatment for each time point for (a) strain RO15 and (b) strain ScottA. Red bar represents the number of upregulated genes, and the blue bar represents the number of downregulated genes. [file 12864_2021_7407_MOESM2_ESM.pdf]

# transcription factors

## a) RO15

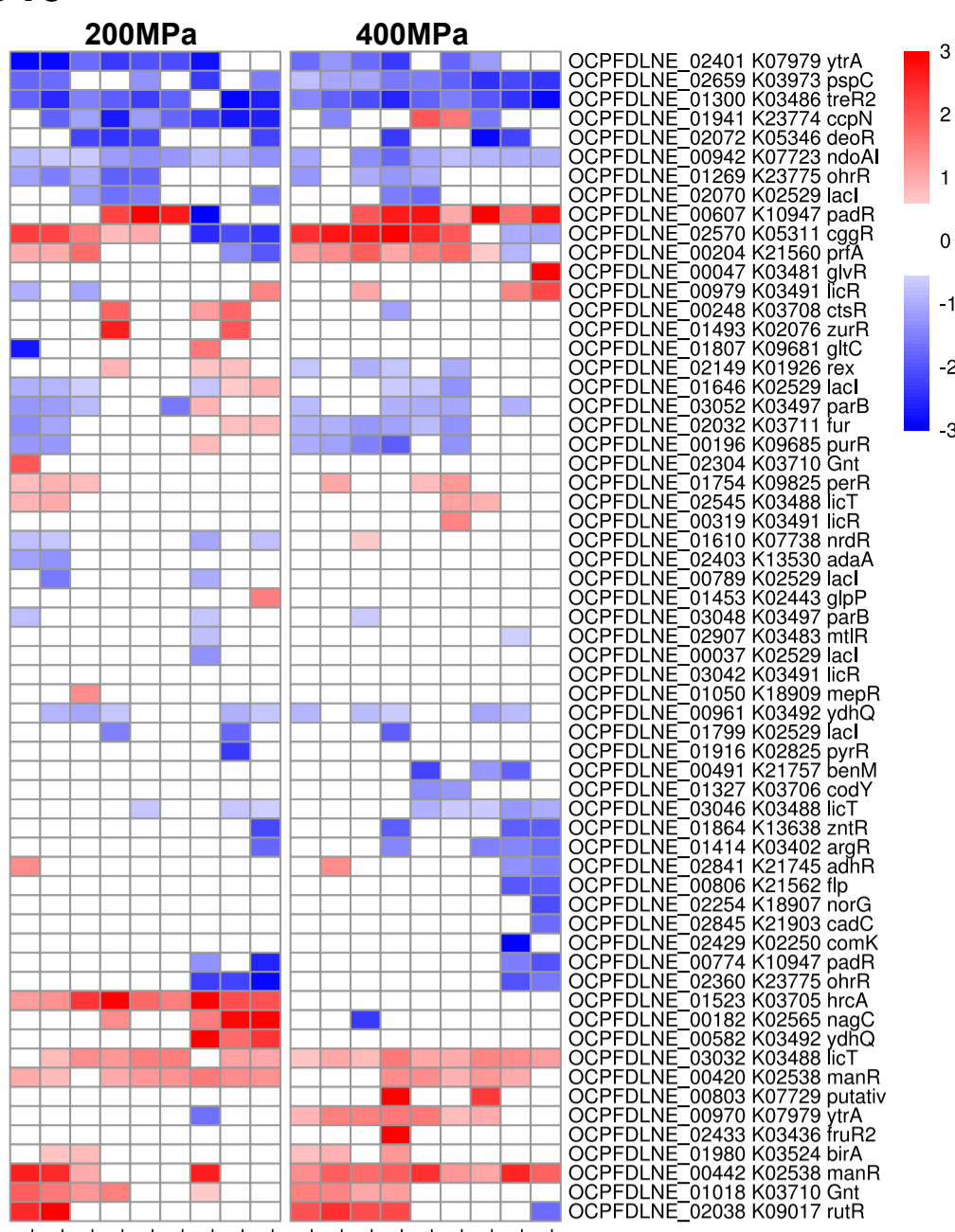

## b) ScottA

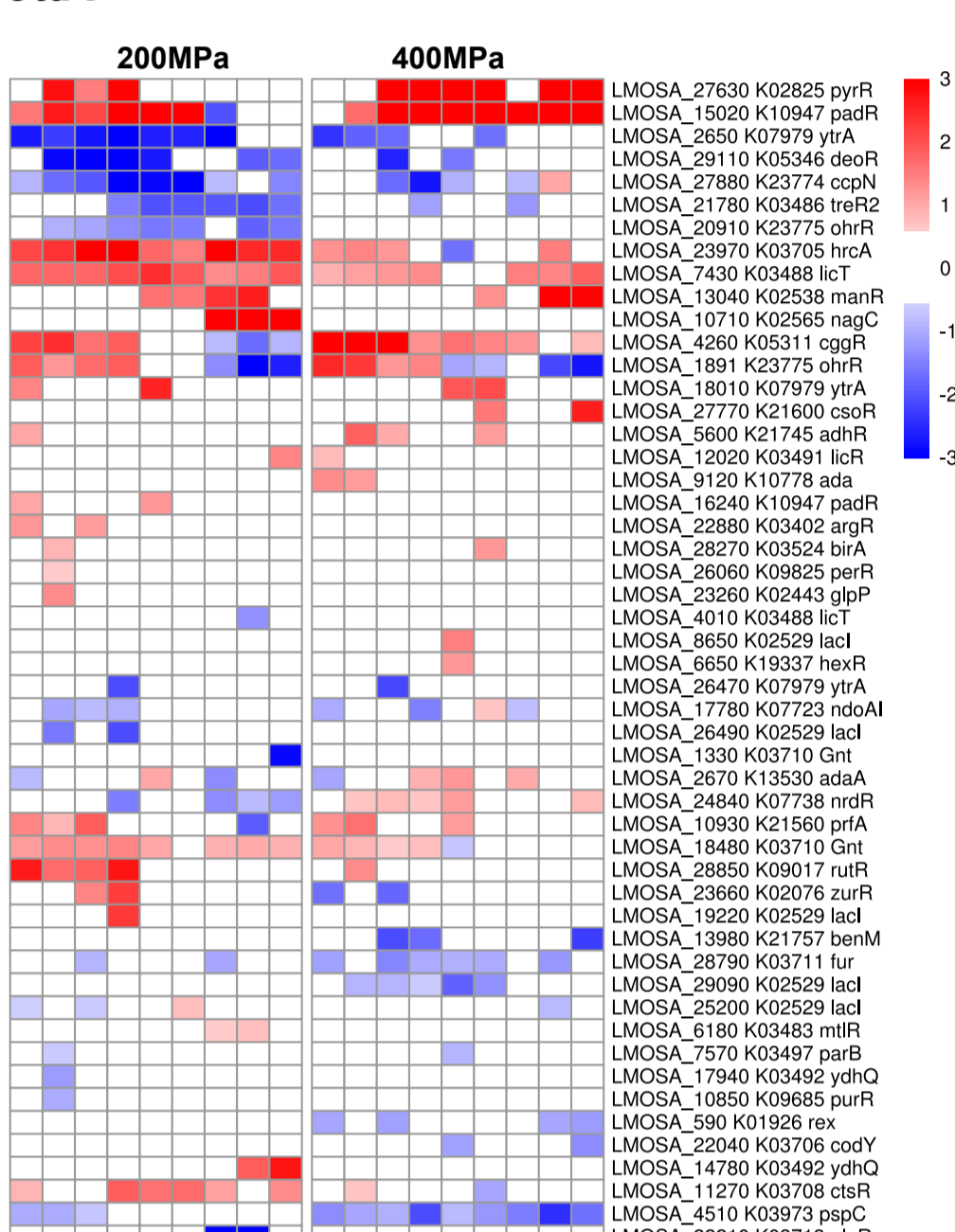

## c) Ortholog genes 200 MPa

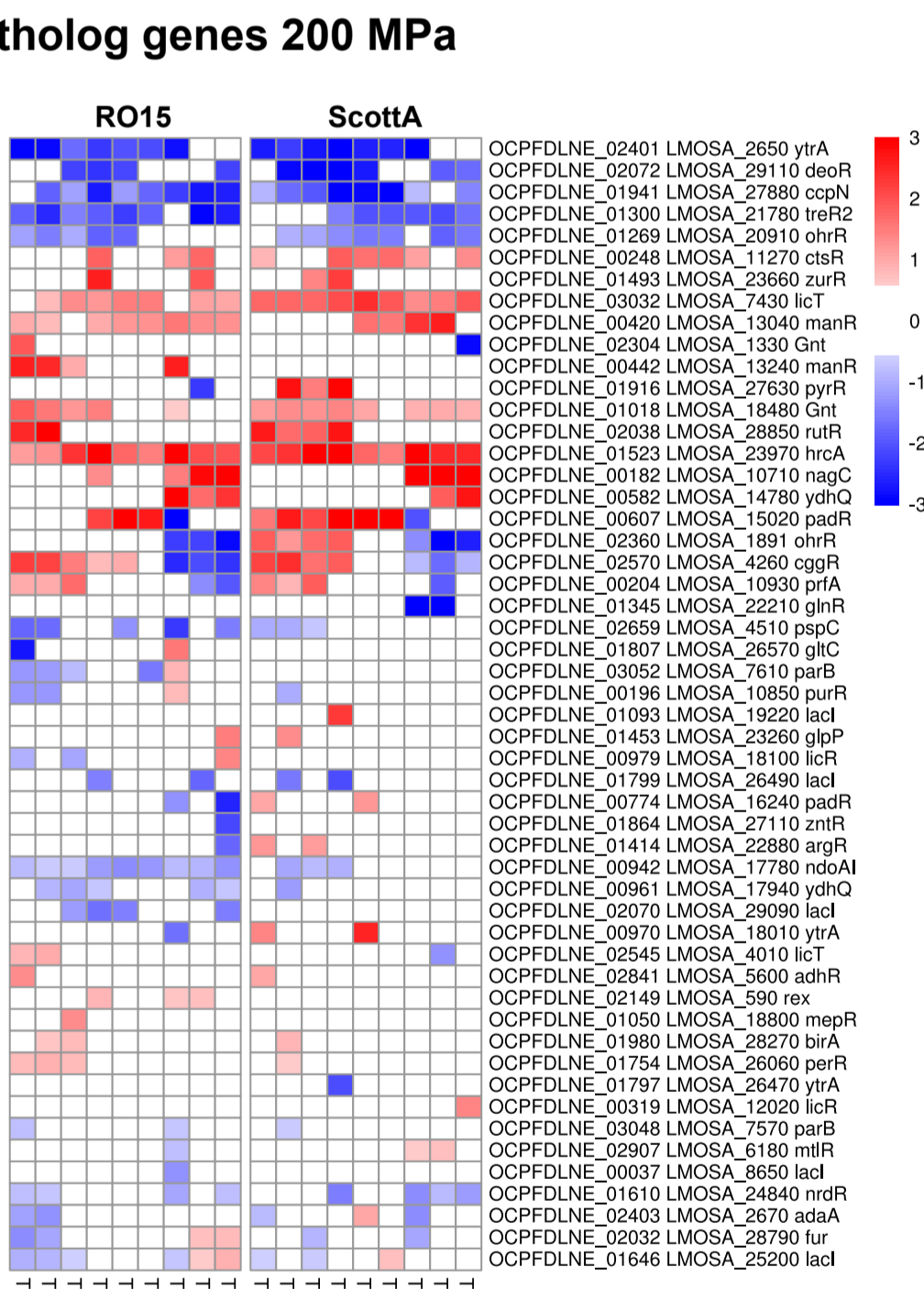

## d) Ortholog genes 400 MPa

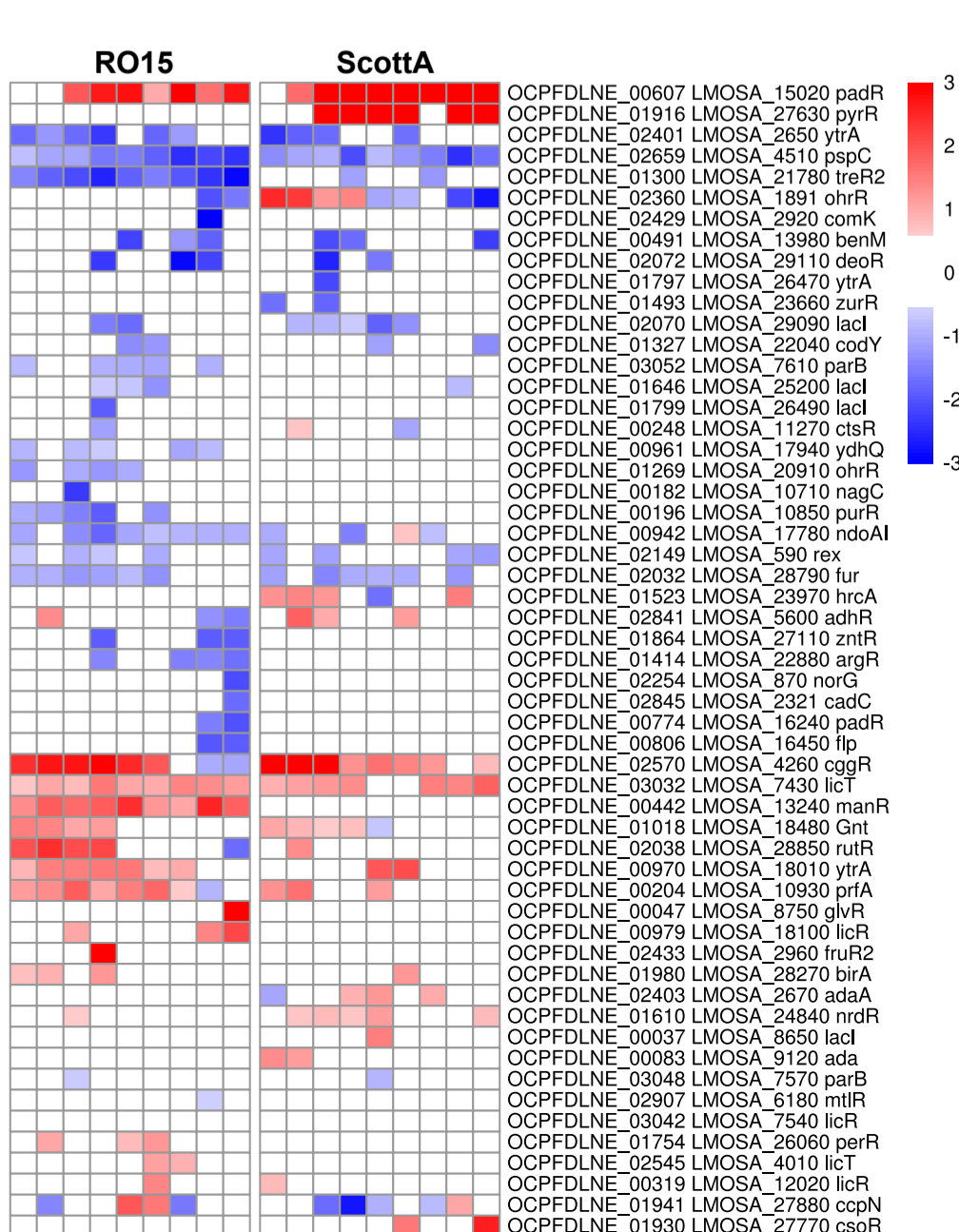

Supplement: Supplementary file 4 — Additional file 4: Figure S4. The log2 fold change heatmap of transcription factor genes. a) RO15 genes, b) ScottA genes, c) ortholog genes for 200 MPa treatment, d) ortholog genes for 400 MPa treatment. The gene name and locus tag of genes for RO15 and ScottA are given at the end of the row. The log2 fold change scale is shown at the right corner. [file 12864_2021_7407_MOESM4_ESM.pdf]

# Transcription machinery

## a) RO15

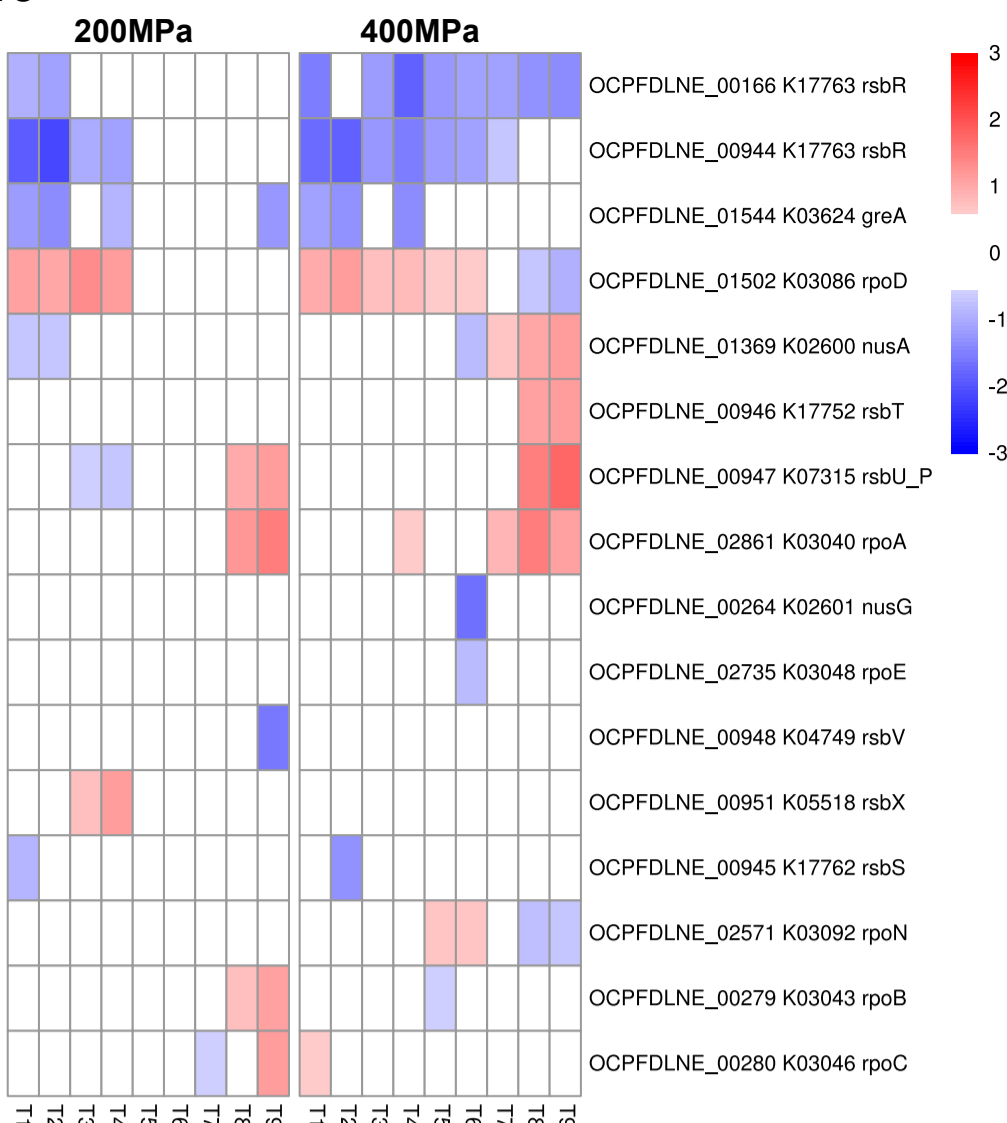

## b) ScottA

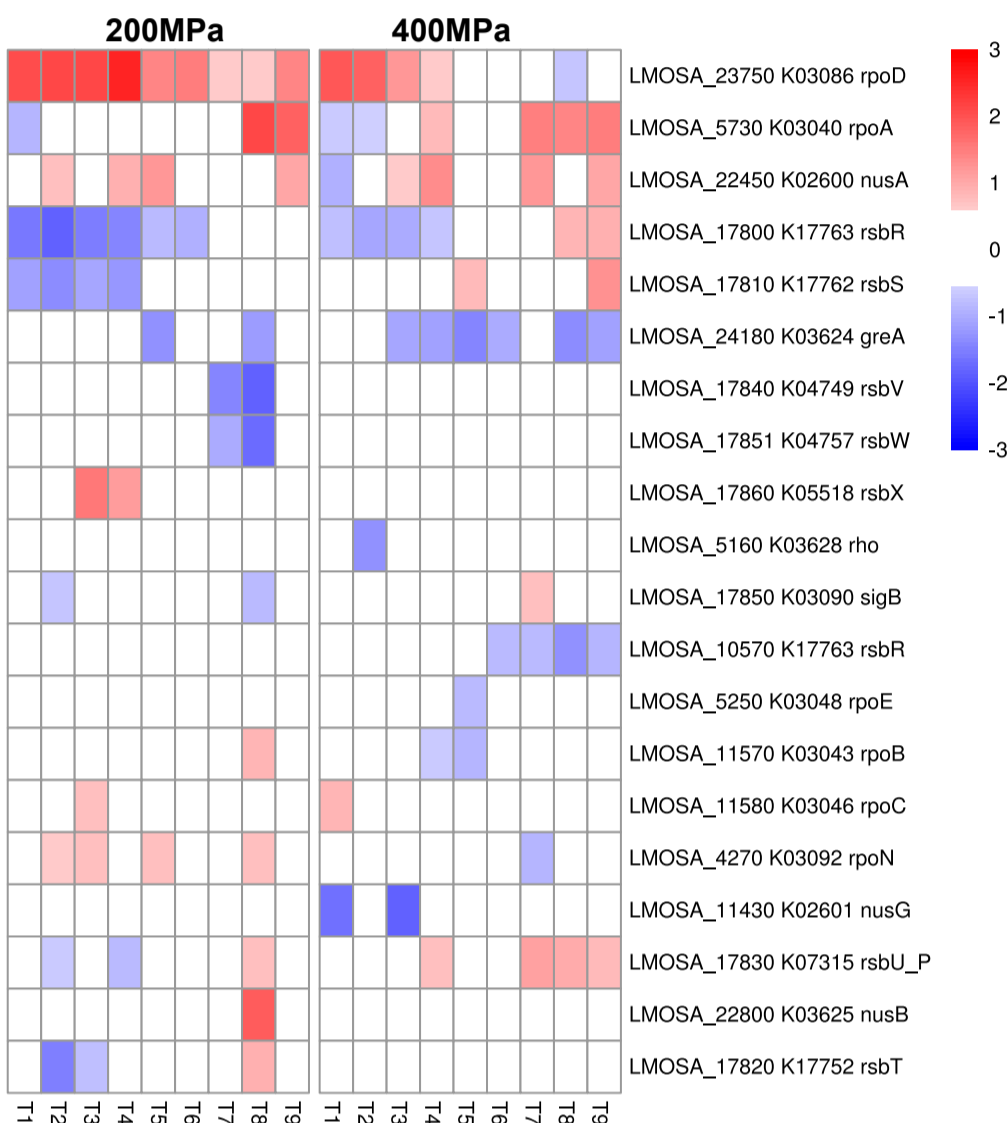

## c) Ortholog genes 200 MPa

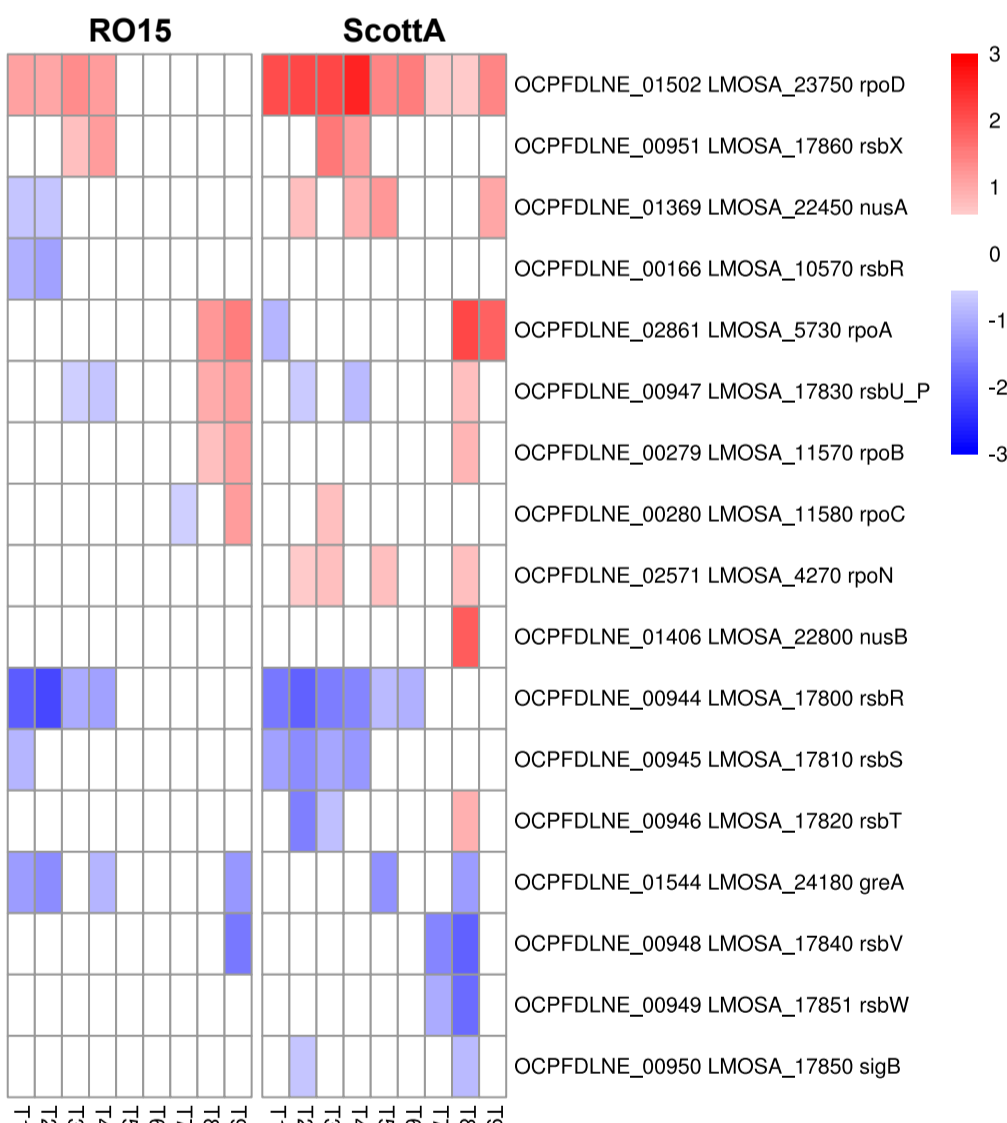

## d) Ortholog genes 400 MPa

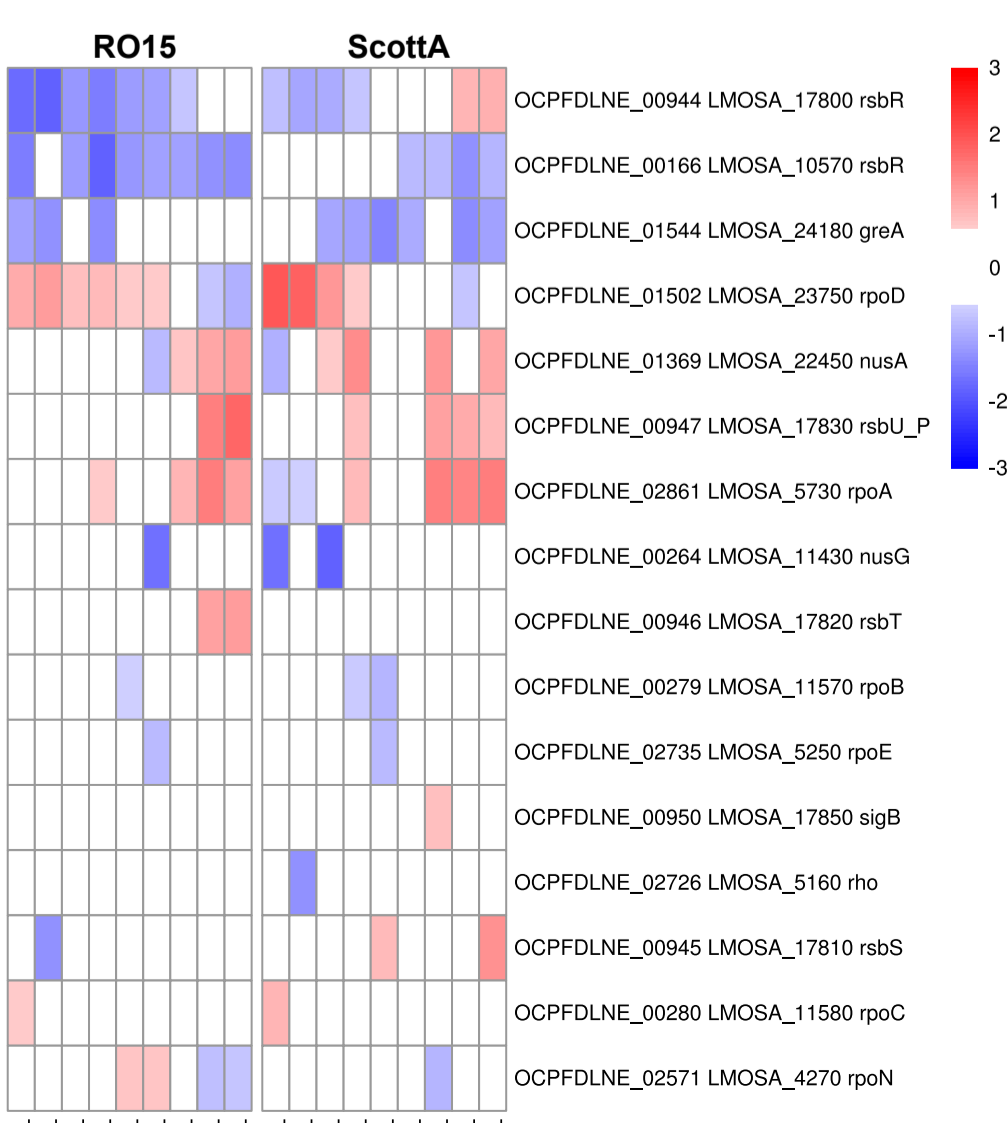

Supplement: Supplementary file 5 — Additional file 5: Figure S5. The log2 fold change heatmap of transcription machinery genes. a) RO15 genes, b) ScottA genes, c) ortholog genes for 200 MPa treatment, d) ortholog genes for 400 MPa treatment. The gene name and locus tag of genes for RO15 and ScottA are given at the end of the row. The log2 fold change scale is shown at the right corner. [file 12864_2021_7407_MOESM5_ESM.pdf]

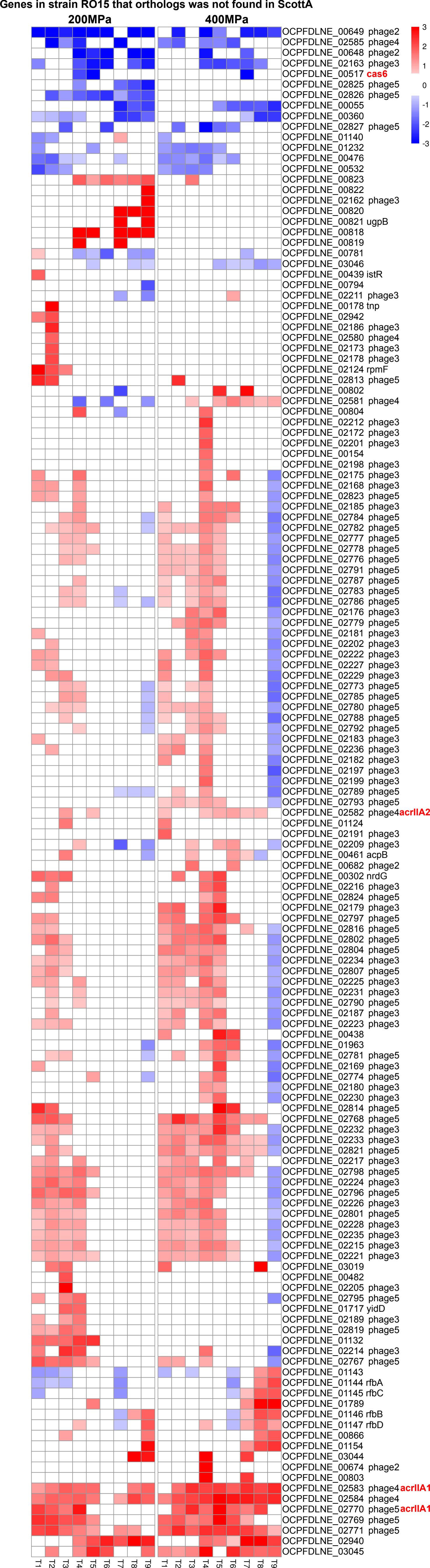

Supplement: Supplementary file 6 — Additional file 6: Figure S6. The log2 fold change heatmap of genes of RO15 that is not found in ScottA. Locus tag of genes are given at the end of the row. Phage genes were indicated with “phage” text. The log2 fold change scale is shown at the right corner. [file 12864_2021_7407_MOESM6_ESM.pdf]

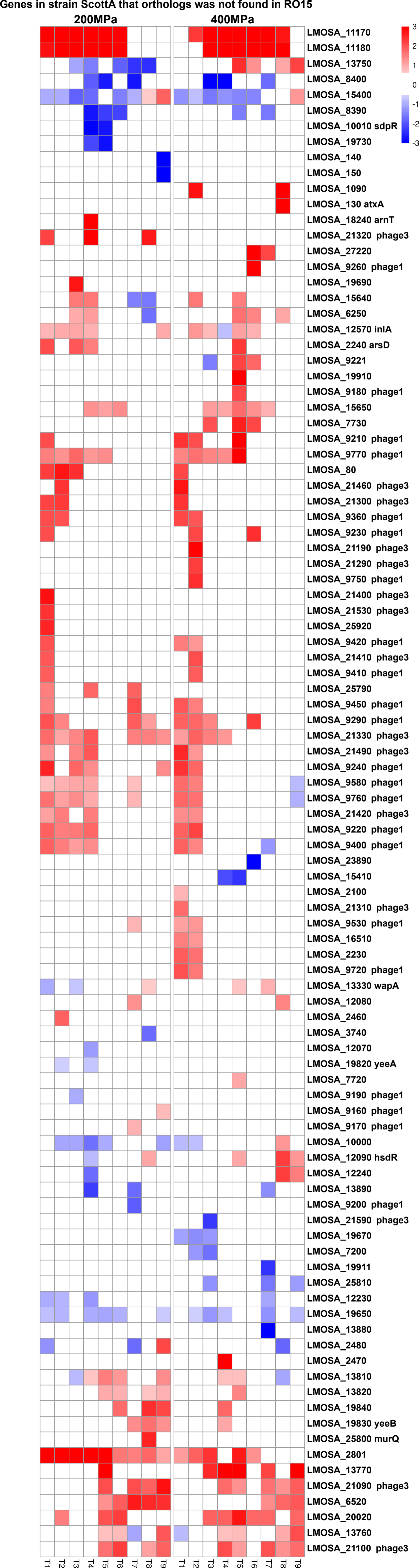

Supplement: Supplementary file 7 — Additional file 7: Figure S7. The log2 fold change heatmap of genes of ScottA that is not found in RO15. Locus tag of genes were given at the end of the row. Phage genes were indicated with “phage” text. The log2 fold change scale is shown at the right corner. [file 12864_2021_7407_MOESM7_ESM.pdf]

a) RO15 ncRNA genes

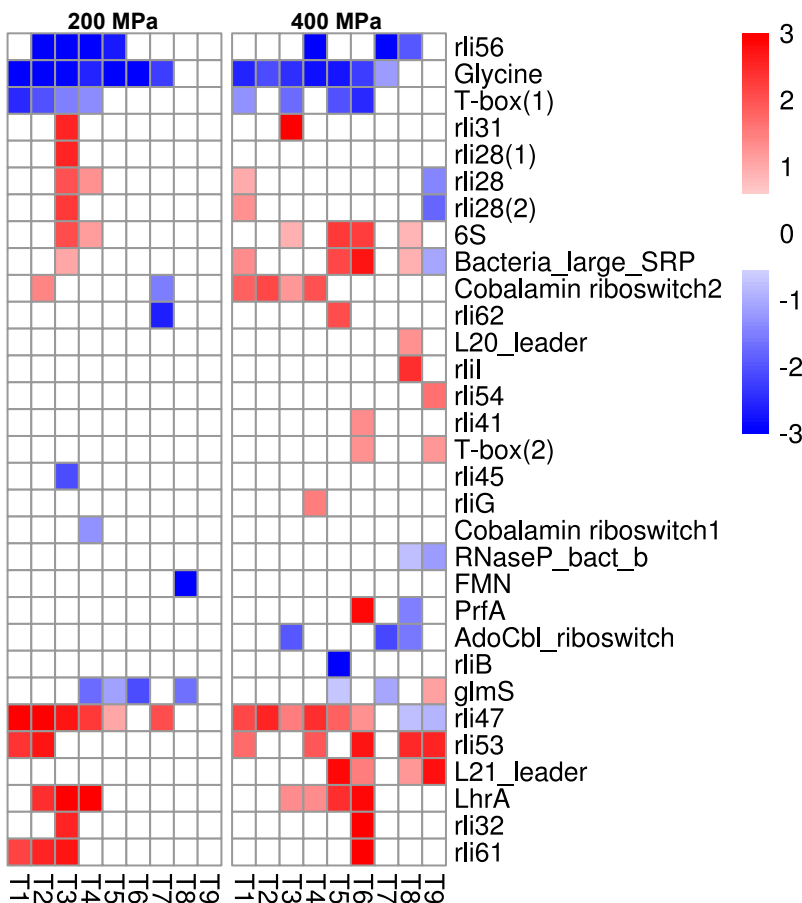

b) ScottA ncRNA genes

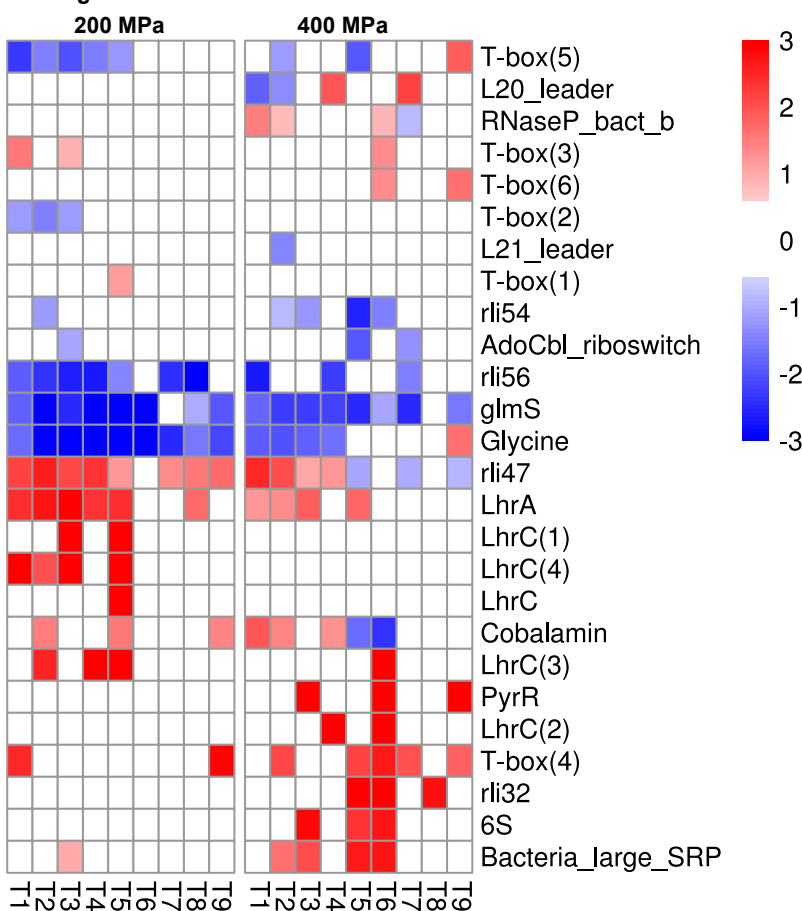

Supplement: Supplementary file 8 — Additional file 8: Figure S8. The log2 fold change heatmap of ncRNA genes. a) log2 fold change values for 200 MPa treatment, b) log2 fold change values for 400 MPa treatment. The gene name and locus tag of genes for RO15 and ScottA were given at the end of the row. The log2 fold change scale is shown at the right corner. [file 12864_2021_7407_MOESM8_ESM.pdf]

# **recG normalized ddPCR / RNA-Seq correlation (200 MPa, 24 h)**

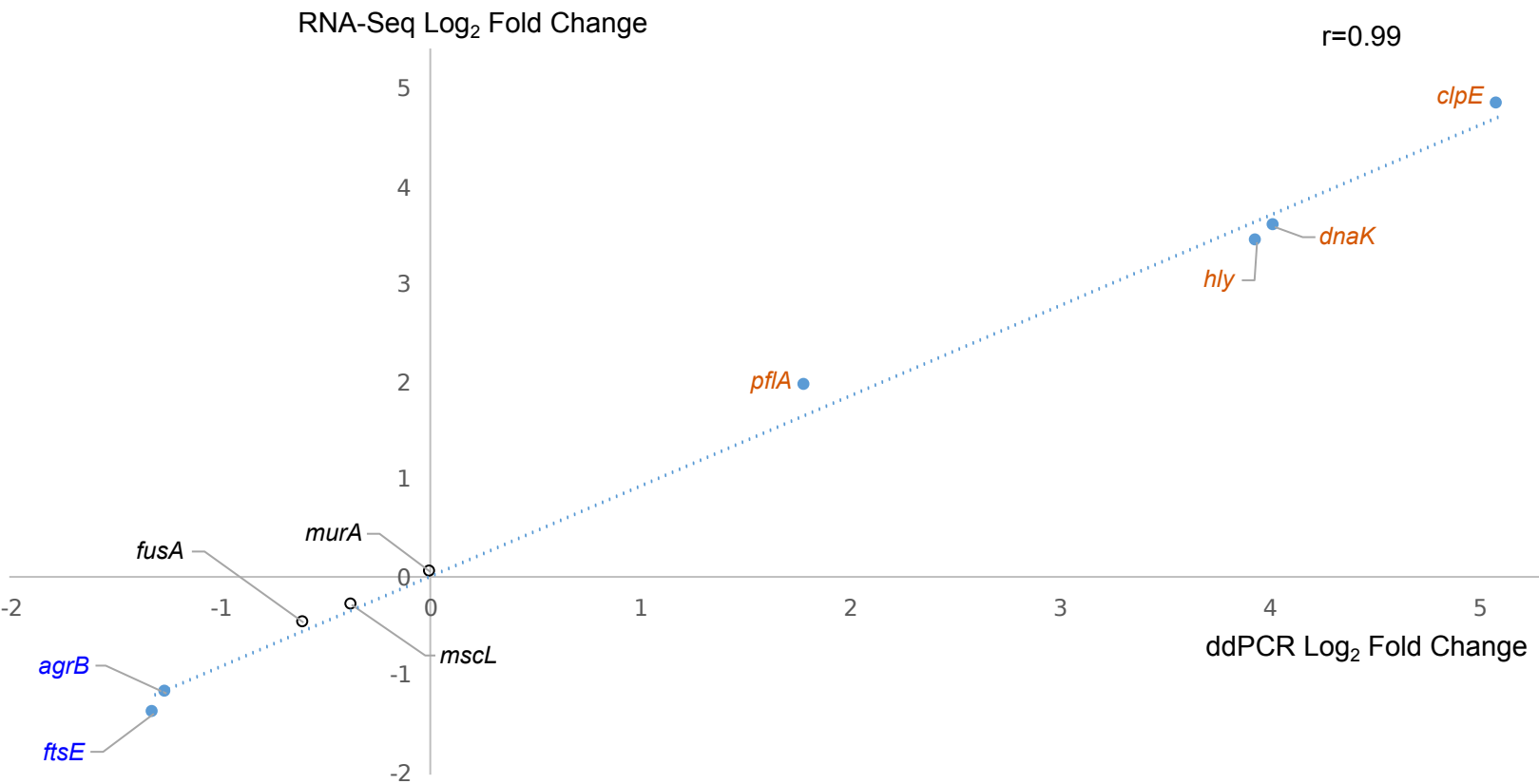

Supplement: Supplementary file 9 — Additional file 9: Figure S9. Figure ddPCR / RNA-seq correlation for log2 fold change. The figure shows log2 fold-changes observed by ddPCR plotted against RNA-seq data for samples of ScottA obtained 24 h after treatment with 200 MPa. Transcripts levels of individual genes obtained by ddPCR were normalized to recG levels. Blue circles represent genes that were significantly differentially expressed in both ddPCR and RNA-seq results. White circles represent genes that were not significantly differentially expressed in both ddPCR and RNA-seq. The orange and blue gene names indicate significant up- and down regulation, respectively. The Pearson correlation coefficient between ddPCR and RNA-seq log2 fold change results was 0.99 (r=0.99). [file 12864_2021_7407_MOESM9_ESM.pdf]

# recG normalized ddPCR / RNA-Seq correlation (400 MPa, 10 min)

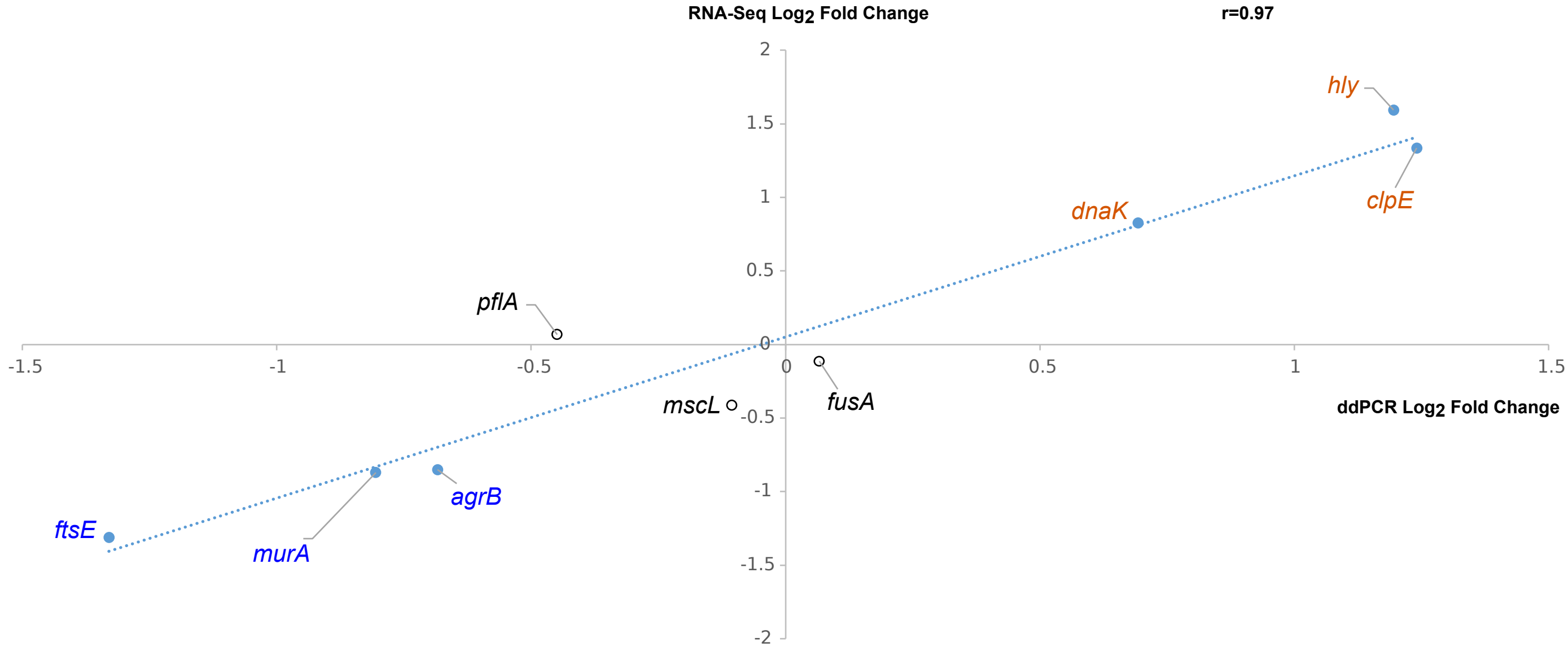

Supplement: Supplementary file 10 — Additional file 10: Figure S10. ddPCR / RNA-seq correlation for log2 fold change. The figure shows log2 fold-changes observed by ddPCR plotted against RNA-seq data for samples of ScottA obtained 10 min after treatment with 400 MPa. Transcripts levels of individual genes obtained by ddPCR were normalized to recG levels. Blue circles represent genes that were significantly differentially expressed in both ddPCR and RNA-seq results. White circles represent genes that were not significantly differentially expressed in both ddPCR and RNA-seq. The orange and blue gene names indicate significant up- and down regulation, respectively. The Pearson correlation coefficient between ddPCR and RNA-seq log2 fold change results was 0.97 (r=0.97). [file 12864_2021_7407_MOESM10_ESM.pdf]

**recG normalized ddPCR / RNA-Seq correlation (400 MPa, 24 h)**

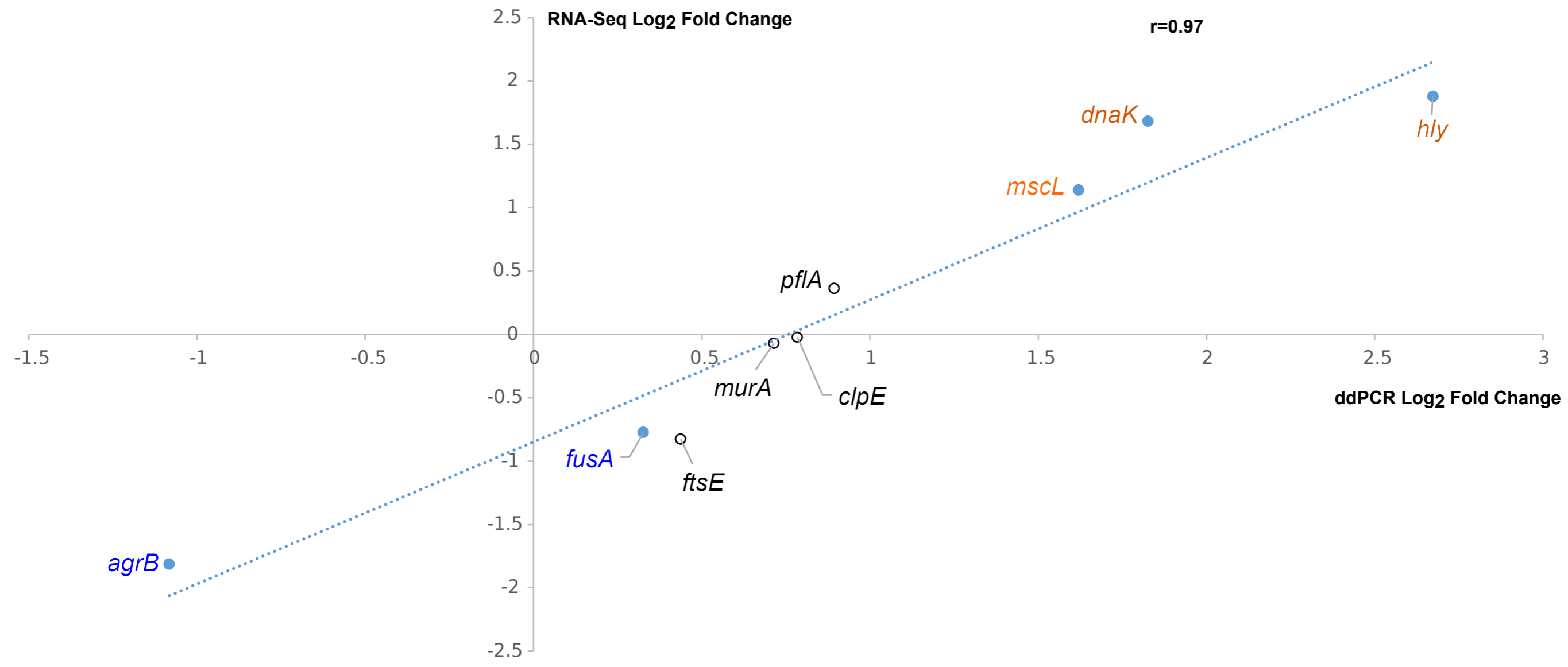

Supplement: Supplementary file 11 — Additional file 11: Figure S11. ddPCR / RNA-seq correlation for log2 fold change. The figure shows log2 fold-changes observed by ddPCR plotted against RNA-seq data for samples of ScottA obtained 24 h after treatment with 400 MPa. Transcript levels of individual genes obtained by ddPCR were normalized to recG levels. Blue circles represent genes that were significantly differentially expressed in both ddPCR and RNA-seq results. White circles represent genes that were not significantly differentially expressed in both ddPCR and RNA-seq. The orange and blue gene names indicate significant up- and down regulation, respectively. The Pearson correlation coefficient between ddPCR and RNA-seq log2 fold change results was 0.97 (r=0.97). [file 12864_2021_7407_MOESM11_ESM.pdf]

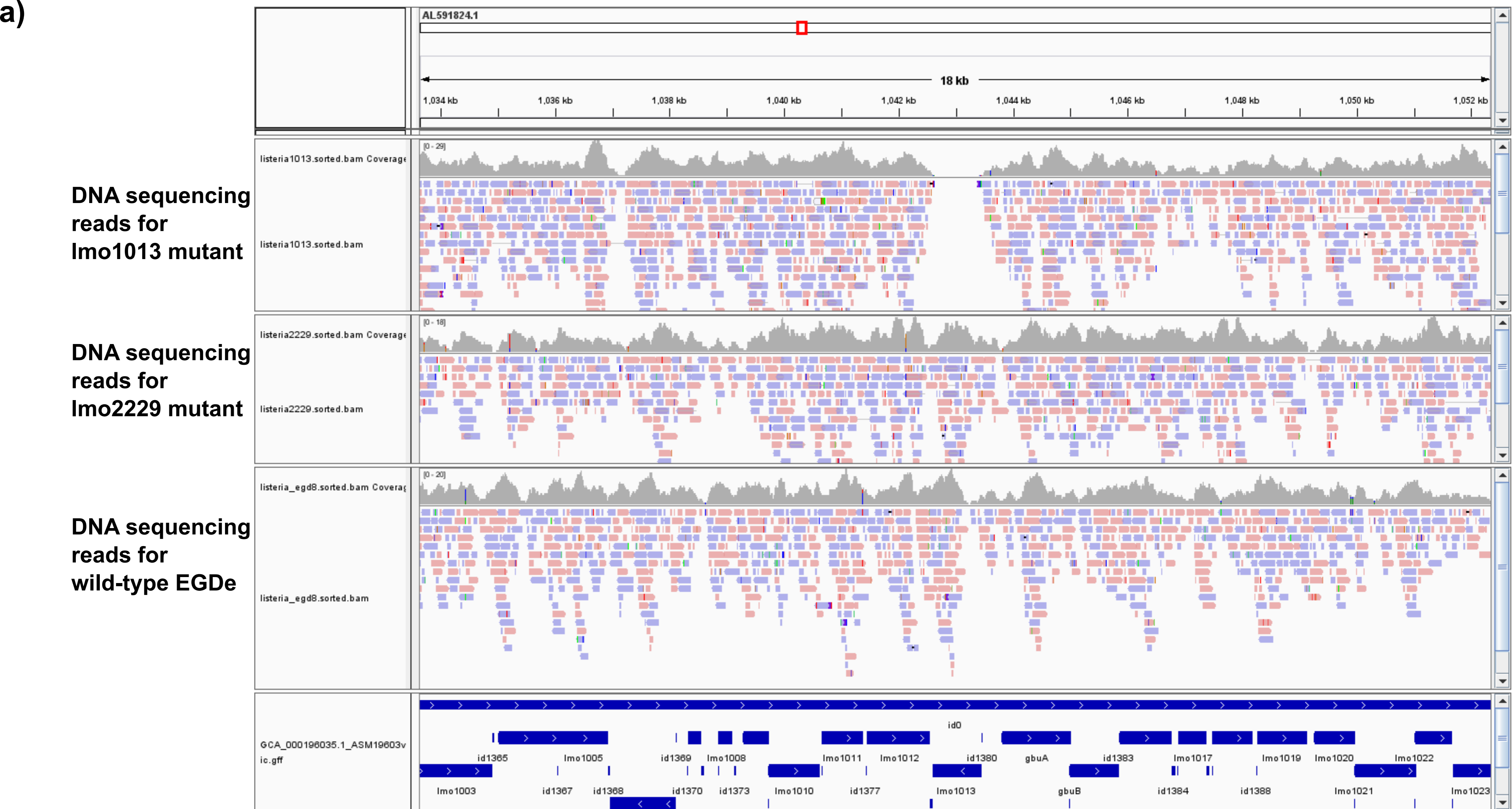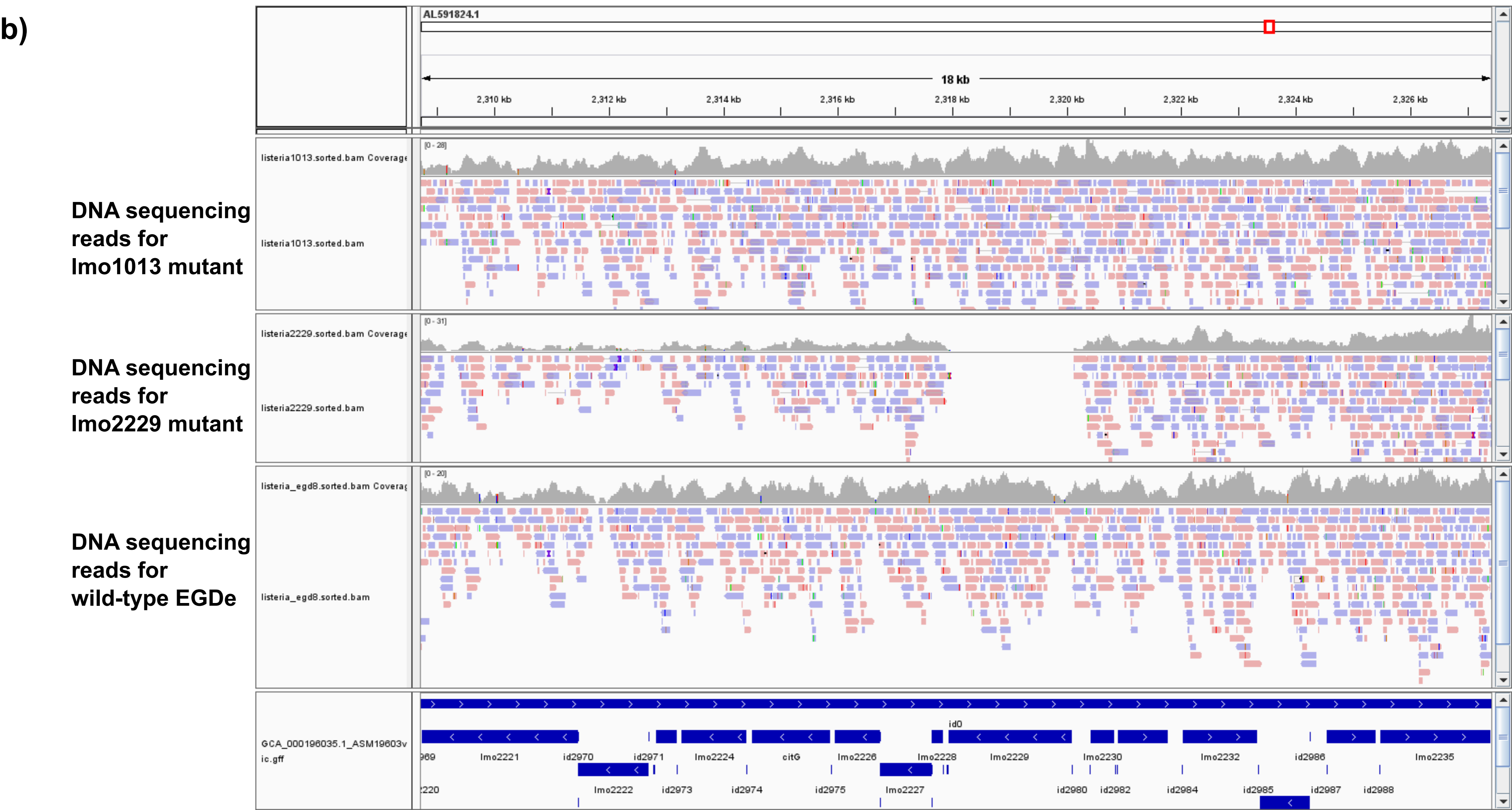

Supplement: Supplementary file 12 — Additional file 12: Figure S12. Visualization of DNA sequencing reads for strain EGDe, Δlmo1013, and Δlmo2229. a) focused the region includes the gene lmo1013, b) focused the region includes the gene lmo2229. [file 12864_2021_7407_MOESM12_ESM.pdf]

# 400 MPa

RO15

ScottA

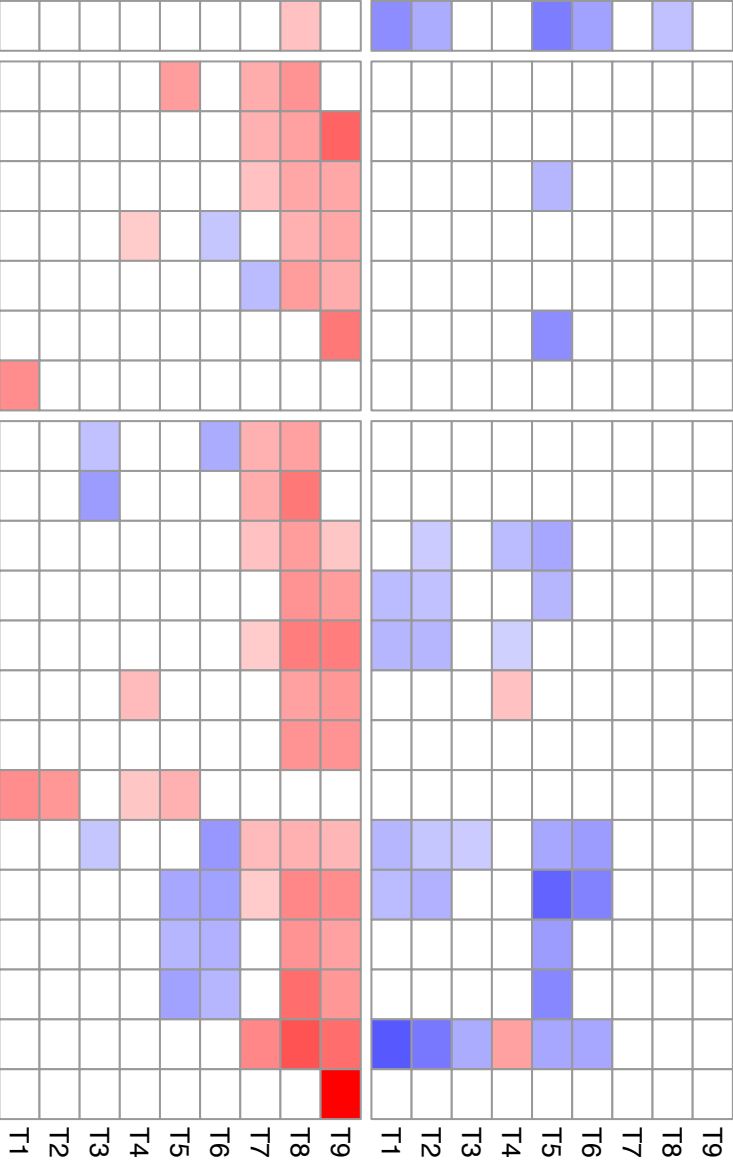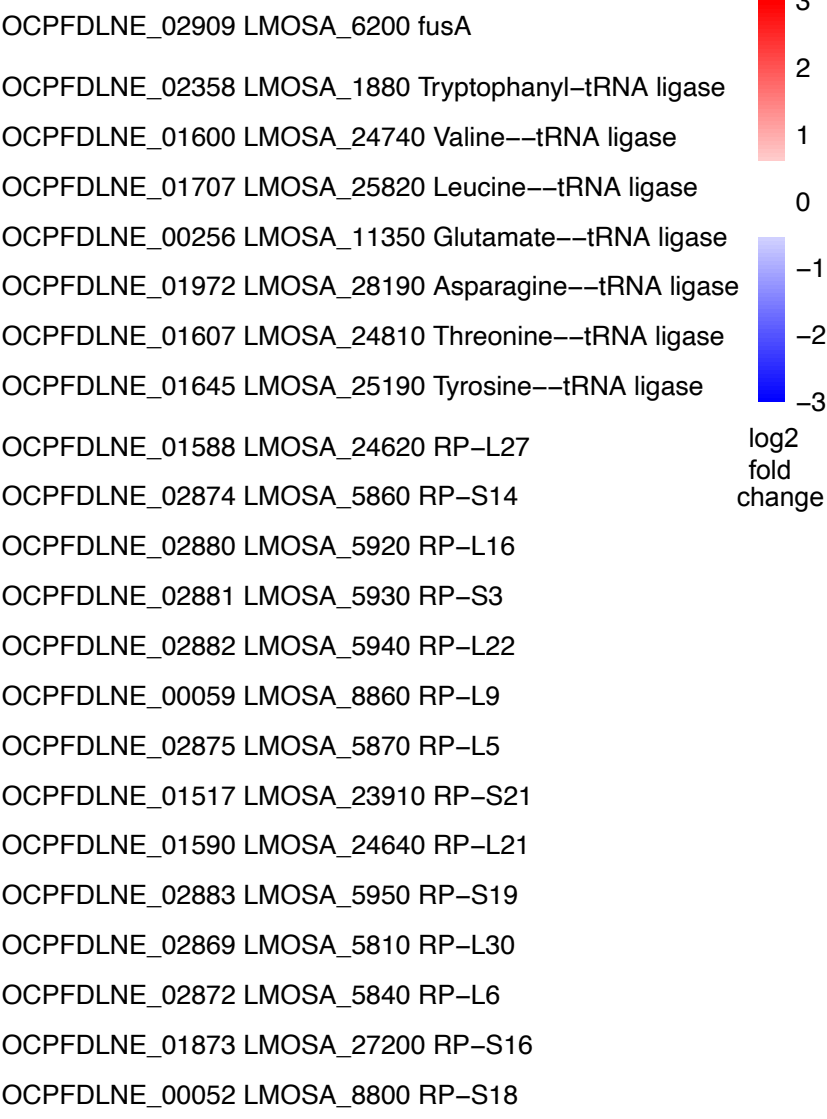

RP: ribosomal protein

Supplement: Supplementary file 14 — Additional file 14: Figure S14. Log2 fold change heatmap of elongation factor, ribosomal protein, and tRNA genes at 400 MPa. [file 12864_2021_7407_MOESM14_ESM.pdf]

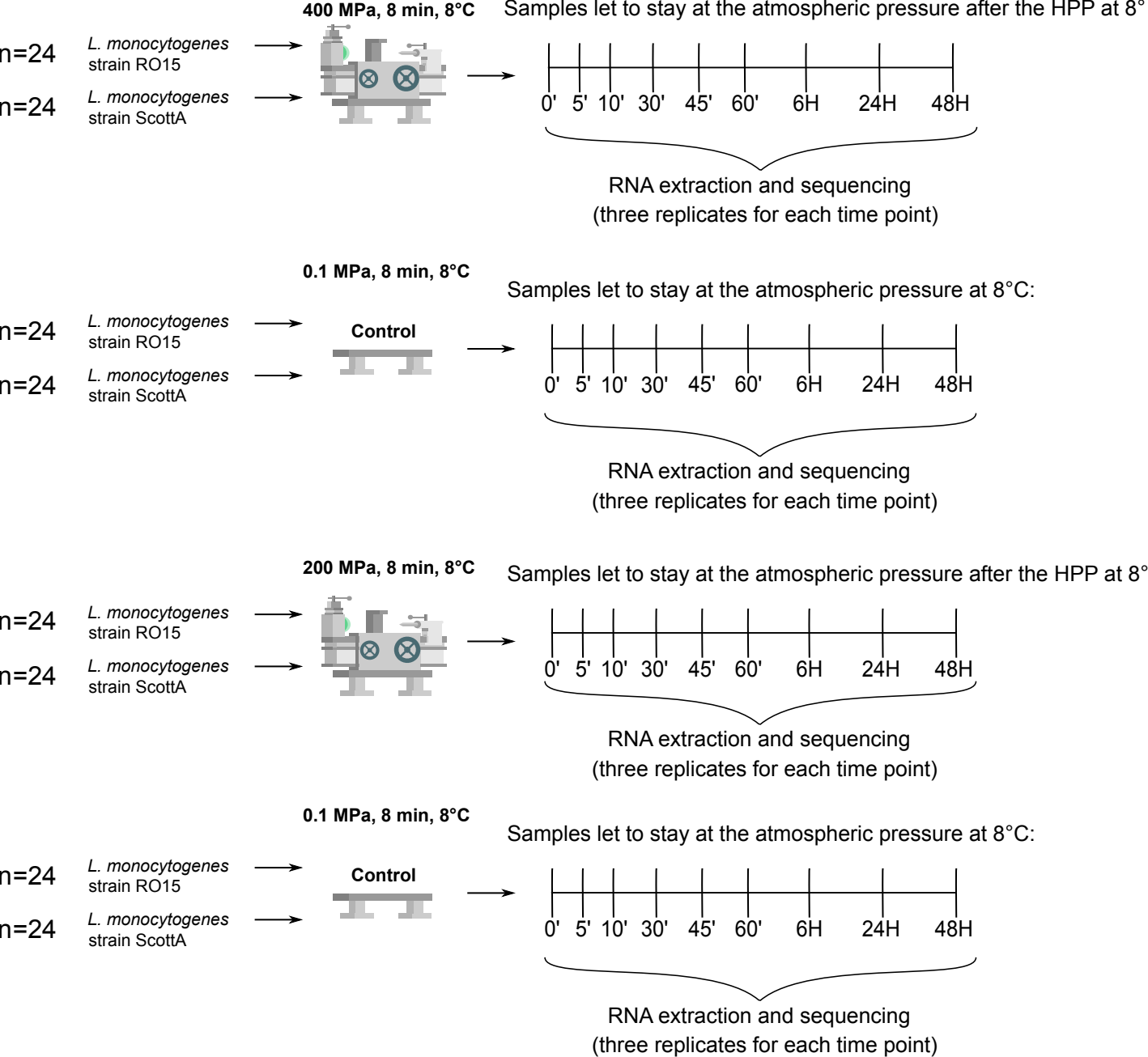

Supplement: Supplementary file 17 — Additional file 17: Figure S17. Visualization of the experimental design. [file 12864_2021_7407_MOESM17_ESM.pdf]
