# Supplementary material for: High-pressure processing-induced transcriptome response during recovery of Listeria monocytogenes
Source: BMC Genomics. 2021 Feb 12;22:117. doi: 10.1186/s12864-021-07407-6 (PMC7881616; doi:10.1186/s12864-021-07407-6)
Supplement: Supplementary file 3 — Additional file 3: Figure S3. The log2 fold change heatmap of propanediol utilization, ethanolamine utilization, and cobalamin biosynthesis genes. A) log2 fold change values for 200 MPa treatment, b) log2 fold change values for 400 MPa treatment. The gene name and locus tag of genes for RO15 and ScottA were given at the end of the row. The log2 fold change scale is shown at the right corner. In addition, gene homology of cobalamin biosynthesis is shown on the second page. [file 12864_2021_7407_MOESM3_ESM.pdf]

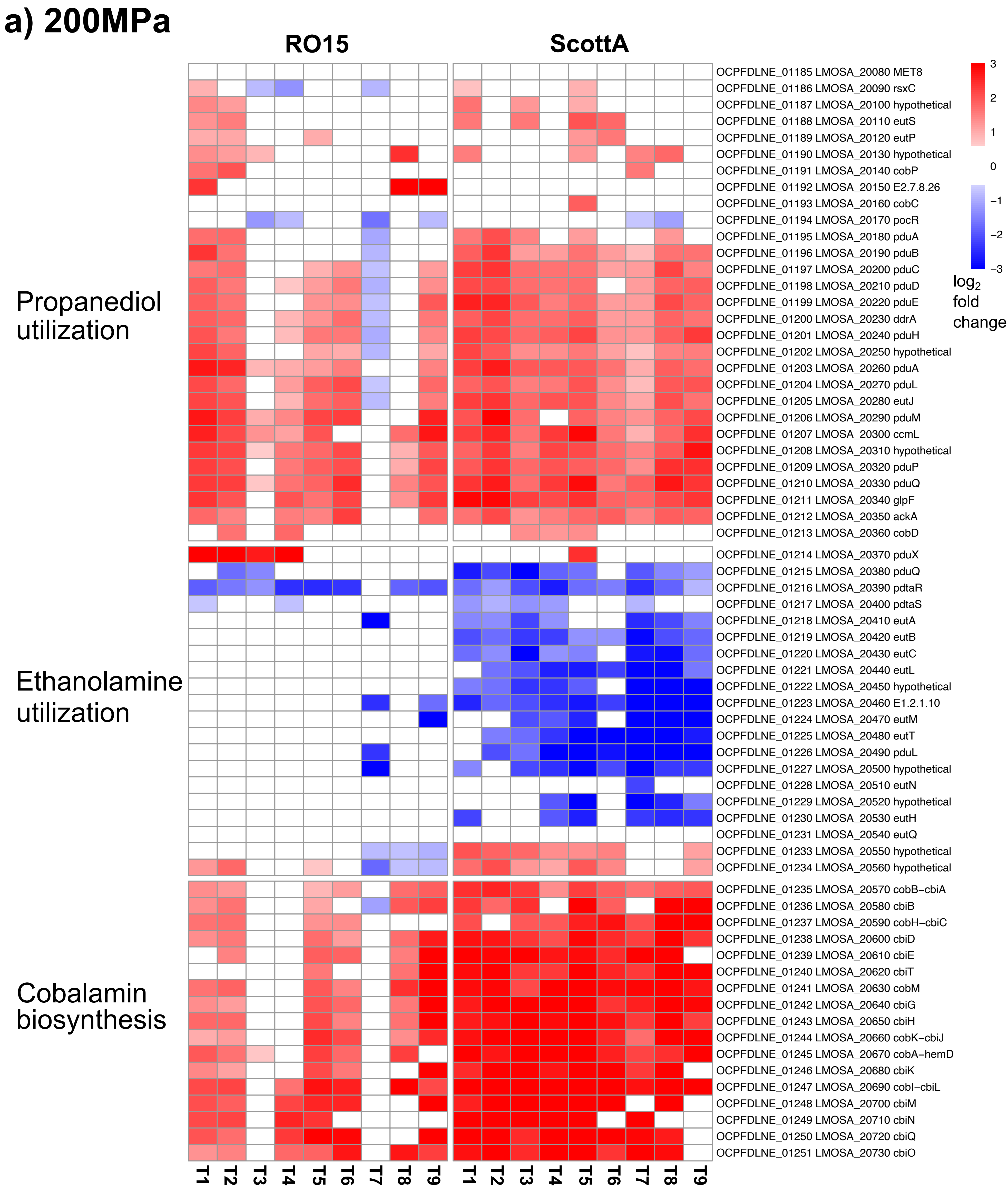
a) 200MPa
RO15
ScottA
Propanediol utilization
Ethanolamine utilization
Cobalamin biosynthesis
OCPFDLNE_01185 LMOSA_20080 MET8
OCPFDLNE_01186 LMOSA_20090 rsxC
OCPFDLNE_01187 LMOSA_20100 hypothetical
OCPFDLNE_01188 LMOSA_20110 eutS
OCPFDLNE_01189 LMOSA_20120 eutP
OCPFDLNE_01190 LMOSA_20130 hypothetical
OCPFDLNE_01191 LMOSA_20140 cobP
OCPFDLNE_01192 LMOSA_20150 E2.7.8.26
OCPFDLNE_01193 LMOSA_20160 cobC
OCPFDLNE_01194 LMOSA_20170 pocR
OCPFDLNE_01195 LMOSA_20180 pduA
OCPFDLNE_01196 LMOSA_20190 pduB
OCPFDLNE_01197 LMOSA_20200 pduC
OCPFDLNE_01198 LMOSA_20210 pduD
OCPFDLNE_01199 LMOSA_20220 pduE
OCPFDLNE_01200 LMOSA_20230 ddrA
OCPFDLNE_01201 LMOSA_20240 pduH
OCPFDLNE_01202 LMOSA_20250 hypothetical
OCPFDLNE_01203 LMOSA_20260 pduA
OCPFDLNE_01204 LMOSA_20270 pduL
OCPFDLNE_01205 LMOSA_20280 eutJ
OCPFDLNE_01206 LMOSA_20290 pduM
OCPFDLNE_01207 LMOSA_20300 ccmL
OCPFDLNE_01208 LMOSA_20310 hypothetical
OCPFDLNE_01209 LMOSA_20320 pduP
OCPFDLNE_01210 LMOSA_20330 pduQ
OCPFDLNE_01211 LMOSA_20340 glpF
OCPFDLNE_01212 LMOSA_20350 ackA
OCPFDLNE_01213 LMOSA_20360 cobD
OCPFDLNE_01214 LMOSA_20370 pduX
OCPFDLNE_01215 LMOSA_20380 pduQ
OCPFDLNE_01216 LMOSA_20390 pdtaR
OCPFDLNE_01217 LMOSA_20400 pdtaS
OCPFDLNE_01218 LMOSA_20410 eutA
OCPFDLNE_01219 LMOSA_20420 eutB
OCPFDLNE_01220 LMOSA_20430 eutC
OCPFDLNE_01221 LMOSA_20440 eutL
OCPFDLNE_01222 LMOSA_20450 hypothetical
OCPFDLNE_01223 LMOSA_20460 E1.2.1.10
OCPFDLNE_01224 LMOSA_20470 eutM
OCPFDLNE_01225 LMOSA_20480 eutT
OCPFDLNE_01226 LMOSA_20490 pduL
OCPFDLNE_01227 LMOSA_20500 hypothetical
OCPFDLNE_01228 LMOSA_20510 eutN
OCPFDLNE_01229 LMOSA_20520 hypothetical
OCPFDLNE_01230 LMOSA_20530 eutH
OCPFDLNE_01231 LMOSA_20540 eutQ
OCPFDLNE_01233 LMOSA_20550 hypothetical
OCPFDLNE_01234 LMOSA_20560 hypothetical
OCPFDLNE_01235 LMOSA_20570 cobB−cbiA
OCPFDLNE_01236 LMOSA_20580 cbiB
OCPFDLNE_01237 LMOSA_20590 cobH−cbiC
OCPFDLNE_01238 LMOSA_20600 cbiD
OCPFDLNE_01239 LMOSA_20610 cbiE
OCPFDLNE_01240 LMOSA_20620 cbiT
OCPFDLNE_01241 LMOSA_20630 cobM
OCPFDLNE_01242 LMOSA_20640 cbiG
OCPFDLNE_01243 LMOSA_20650 cbiH
OCPFDLNE_01244 LMOSA_20660 cobK−cbiJ
OCPFDLNE_01245 LMOSA_20670 cobA−hemD
OCPFDLNE_01246 LMOSA_20680 cbiK
OCPFDLNE_01247 LMOSA_20690 cobI−cbiL
OCPFDLNE_01248 LMOSA_20700 cbiM
OCPFDLNE_01249 LMOSA_20710 cbiN
OCPFDLNE_01250 LMOSA_20720 cbiQ
OCPFDLNE_01251 LMOSA_20730 cbiO
T1 T2 T3 T4 T5 T6 T7 T8 T9
T1 T2 T3 T4 T5 T6 T7 T8 T9
3
2
1
0
−1
−2
−3
log2 fold change

b) 400MPa

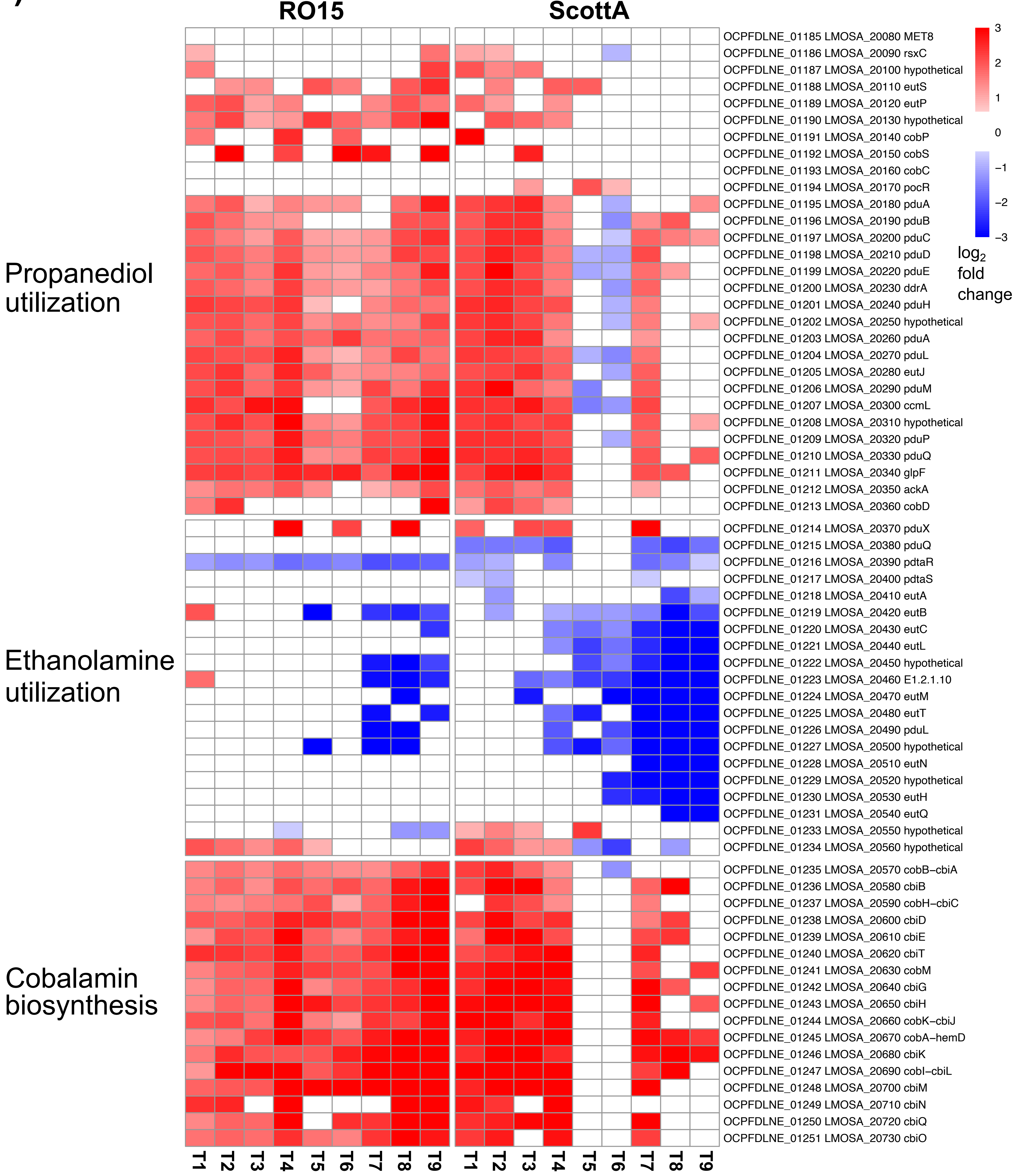
RO15
ScottA
OCPFDLNE_01185 LMOSA_20080 MET8
OCPFDLNE_01186 LMOSA_20090 rsxC
OCPFDLNE_01187 LMOSA_20100 hypothetical
OCPFDLNE_01188 LMOSA_20110 eutS
OCPFDLNE_01189 LMOSA_20120 eutP
OCPFDLNE_01190 LMOSA_20130 hypothetical
OCPFDLNE_01191 LMOSA_20140 cobP
OCPFDLNE_01192 LMOSA_20150 cobS
OCPFDLNE_01193 LMOSA_20160 cobC
OCPFDLNE_01194 LMOSA_20170 pocR
OCPFDLNE_01195 LMOSA_20180 pduA
OCPFDLNE_01196 LMOSA_20190 pduB
OCPFDLNE_01197 LMOSA_20200 pduC
OCPFDLNE_01198 LMOSA_20210 pduD
OCPFDLNE_01199 LMOSA_20220 pduE
OCPFDLNE_01200 LMOSA_20230 ddrA
OCPFDLNE_01201 LMOSA_20240 pduH
OCPFDLNE_01202 LMOSA_20250 hypothetical
OCPFDLNE_01203 LMOSA_20260 pduA
OCPFDLNE_01204 LMOSA_20270 pduL
OCPFDLNE_01205 LMOSA_20280 eutJ
OCPFDLNE_01206 LMOSA_20290 pduM
OCPFDLNE_01207 LMOSA_20300 ccmL
OCPFDLNE_01208 LMOSA_20310 hypothetical
OCPFDLNE_01209 LMOSA_20320 pduP
OCPFDLNE_01210 LMOSA_20330 pduQ
OCPFDLNE_01211 LMOSA_20340 glpF
OCPFDLNE_01212 LMOSA_20350 ackA
OCPFDLNE_01213 LMOSA_20360 cobD
OCPFDLNE_01214 LMOSA_20370 pduX
OCPFDLNE_01215 LMOSA_20380 pduQ
OCPFDLNE_01216 LMOSA_20390 pdtaR
OCPFDLNE_01217 LMOSA_20400 pdtaS
OCPFDLNE_01218 LMOSA_20410 eutA
OCPFDLNE_01219 LMOSA_20420 eutB
OCPFDLNE_01220 LMOSA_20430 eutC
OCPFDLNE_01221 LMOSA_20440 eutL
OCPFDLNE_01222 LMOSA_20450 hypothetical
OCPFDLNE_01223 LMOSA_20460 E1.2.1.10
OCPFDLNE_01224 LMOSA_20470 eutM
OCPFDLNE_01225 LMOSA_20480 eutT
OCPFDLNE_01226 LMOSA_20490 pduL
OCPFDLNE_01227 LMOSA_20500 hypothetical
OCPFDLNE_01228 LMOSA_20510 eutN
OCPFDLNE_01229 LMOSA_20520 hypothetical
OCPFDLNE_01230 LMOSA_20530 eutH
OCPFDLNE_01231 LMOSA_20540 eutQ
OCPFDLNE_01233 LMOSA_20550 hypothetical
OCPFDLNE_01234 LMOSA_20560 hypothetical
OCPFDLNE_01235 LMOSA_20570 cobB−cbiA
OCPFDLNE_01236 LMOSA_20580 cbiB
OCPFDLNE_01237 LMOSA_20590 cobH−cbiC
OCPFDLNE_01238 LMOSA_20600 cbiD
OCPFDLNE_01239 LMOSA_20610 cbiE
OCPFDLNE_01240 LMOSA_20620 cbiT
OCPFDLNE_01241 LMOSA_20630 cobM
OCPFDLNE_01242 LMOSA_20640 cbiG
OCPFDLNE_01243 LMOSA_20650 cbiH
OCPFDLNE_01244 LMOSA_20660 cobK−cbiJ
OCPFDLNE_01245 LMOSA_20670 cobA−hemD
OCPFDLNE_01246 LMOSA_20680 cbiK
OCPFDLNE_01247 LMOSA_20690 cobI−cbiL
OCPFDLNE_01248 LMOSA_20700 cbiM
OCPFDLNE_01249 LMOSA_20710 cbiN
OCPFDLNE_01250 LMOSA_20720 cbiQ
OCPFDLNE_01251 LMOSA_20730 cbiO
Propanediol utilization
Ethanolamine utilization
Cobalamin biosynthesis
T1 T2 T3 T4 T5 T6 T7 T8 T9
T1 T2 T3 T4 T5 T6 T7 T8 T9
3
2
1
0
−1
−2
−3
log2 fold change

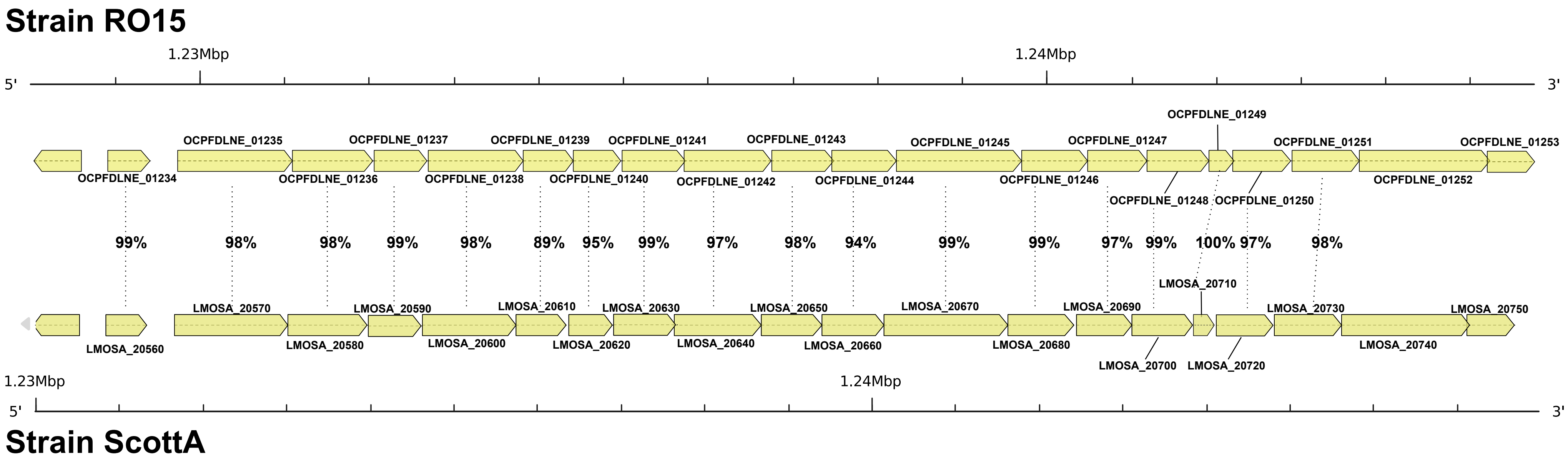
Strain RO15
1.23Mbp
1.24Mbp
5'
3'
OCPFDLNE_01234
OCPFDLNE_01235
OCPFDLNE_01236
OCPFDLNE_01237
OCPFDLNE_01238
OCPFDLNE_01239
OCPFDLNE_01240
OCPFDLNE_01241
OCPFDLNE_01242
OCPFDLNE_01243
OCPFDLNE_01244
OCPFDLNE_01245
OCPFDLNE_01246
OCPFDLNE_01247
OCPFDLNE_01248
OCPFDLNE_01249
OCPFDLNE_01250
OCPFDLNE_01251
OCPFDLNE_01252
OCPFDLNE_01253
99%
98%
98%
99%
98%
89%
95%
99%
97%
98%
94%
99%
99%
97%
99%
100%
97%
98%
LMOSA_20560
LMOSA_20570
LMOSA_20580
LMOSA_20590
LMOSA_20600
LMOSA_20610
LMOSA_20620
LMOSA_20630
LMOSA_20640
LMOSA_20650
LMOSA_20660
LMOSA_20670
LMOSA_20680
LMOSA_20690
LMOSA_20700
LMOSA_20710
LMOSA_20720
LMOSA_20730
LMOSA_20740
LMOSA_20750
1.23Mbp
1.24Mbp
5'
3'
Strain ScottA
