# Supplementary material for: High-pressure processing-induced transcriptome response during recovery of Listeria monocytogenes
Source: BMC Genomics. 2021 Feb 12;22:117. doi: 10.1186/s12864-021-07407-6 (PMC7881616; doi:10.1186/s12864-021-07407-6)

a) 200 MPa Ortholog Genes

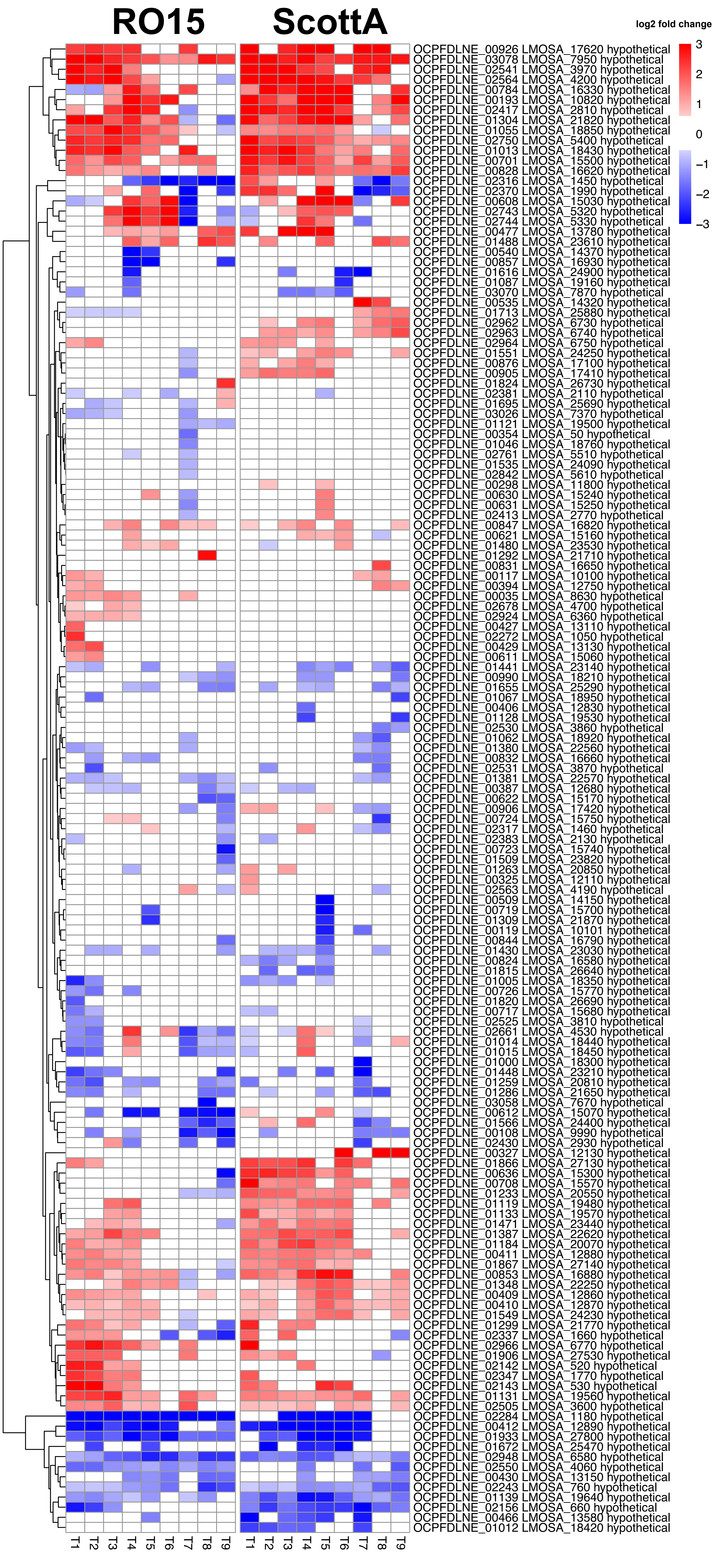

b) 400 MPa Ortholog Genes

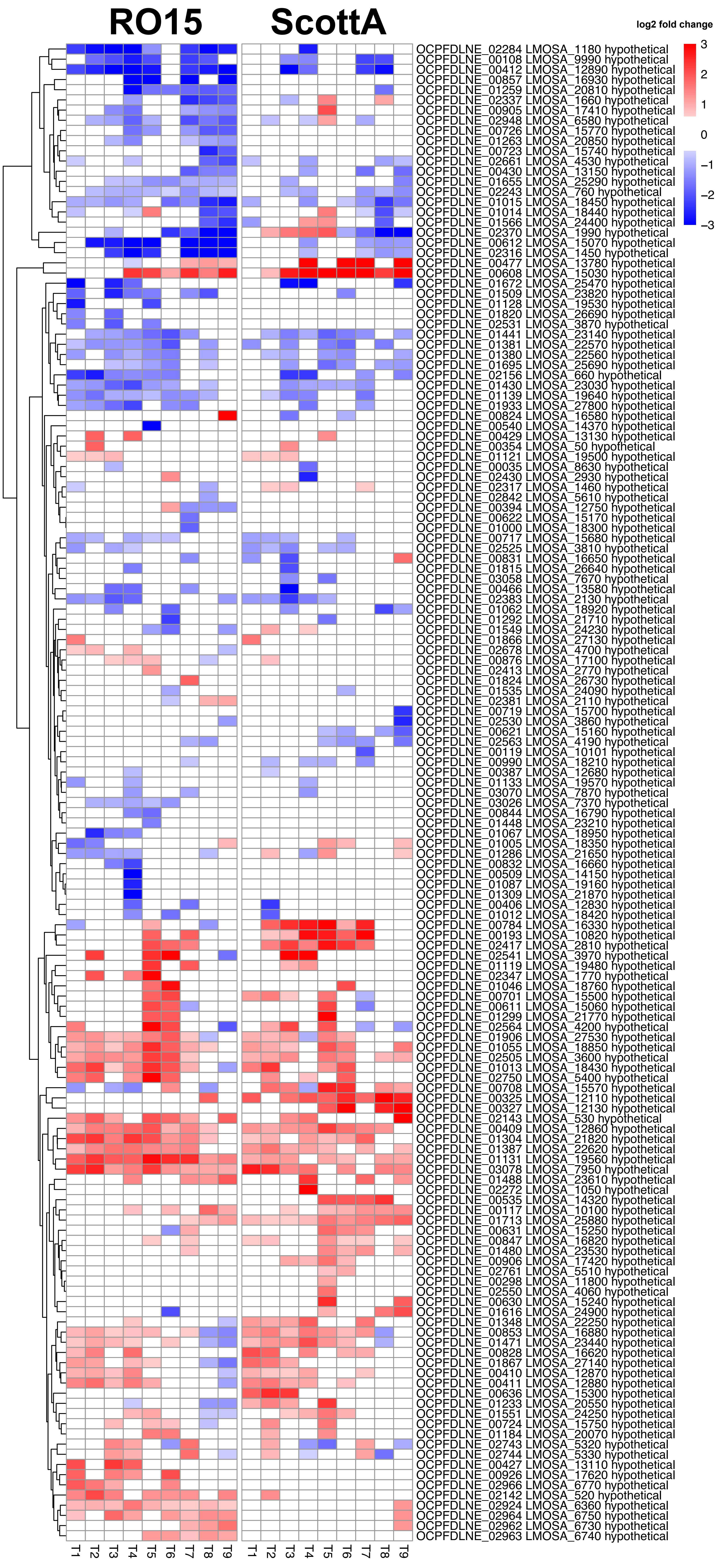

c) DE only at 200 MPa treatment

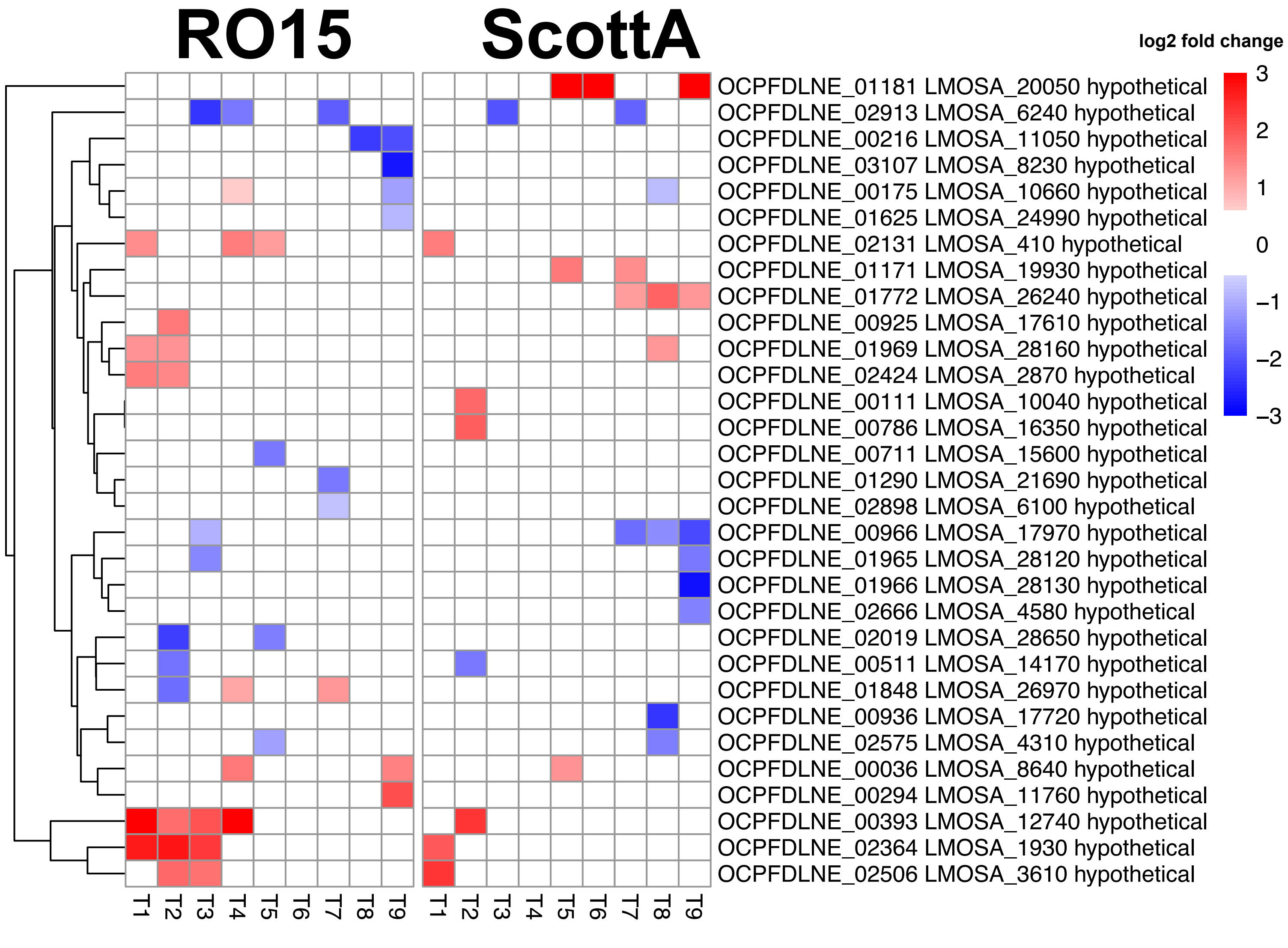

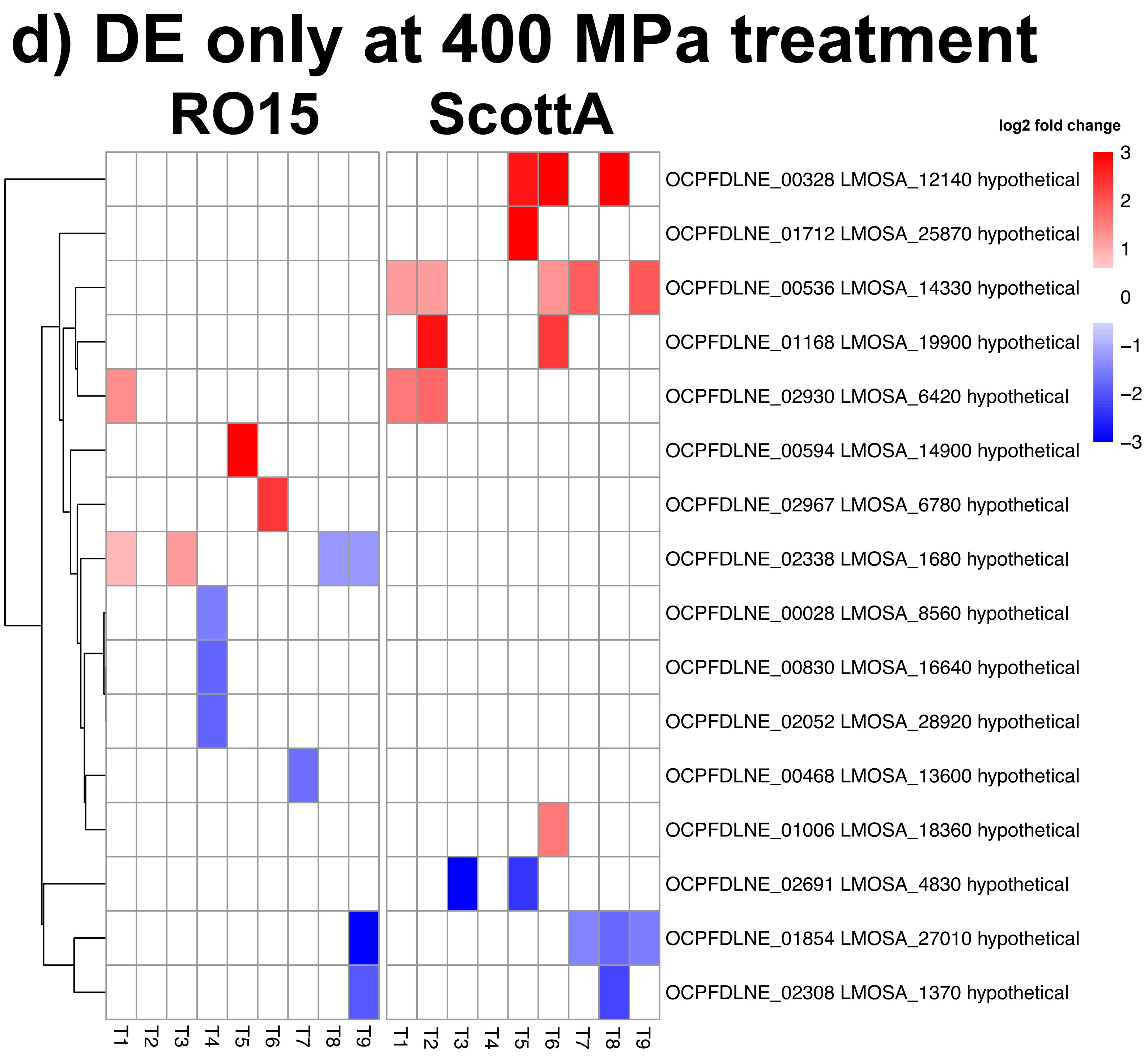
d) DE only at 400 MPa treatment
RO15
ScottA
log2 fold change
3
2
1
0
−1
−2
−3
OCPFDLNE_00328 LMOSA_12140 hypothetical
OCPFDLNE_01712 LMOSA_25870 hypothetical
OCPFDLNE_00536 LMOSA_14330 hypothetical
OCPFDLNE_01168 LMOSA_19900 hypothetical
OCPFDLNE_02930 LMOSA_6420 hypothetical
OCPFDLNE_00594 LMOSA_14900 hypothetical
OCPFDLNE_02967 LMOSA_6780 hypothetical
OCPFDLNE_02338 LMOSA_1680 hypothetical
OCPFDLNE_00028 LMOSA_8560 hypothetical
OCPFDLNE_00830 LMOSA_16640 hypothetical
OCPFDLNE_02052 LMOSA_28920 hypothetical
OCPFDLNE_00468 LMOSA_13600 hypothetical
OCPFDLNE_01006 LMOSA_18360 hypothetical
OCPFDLNE_02691 LMOSA_4830 hypothetical
OCPFDLNE_01854 LMOSA_27010 hypothetical
OCPFDLNE_02308 LMOSA_1370 hypothetical
T1 T2 T3 T4 T5 T6 T7 T8 T9
T1 T2 T3 T4 T5 T6 T7 T8 T9

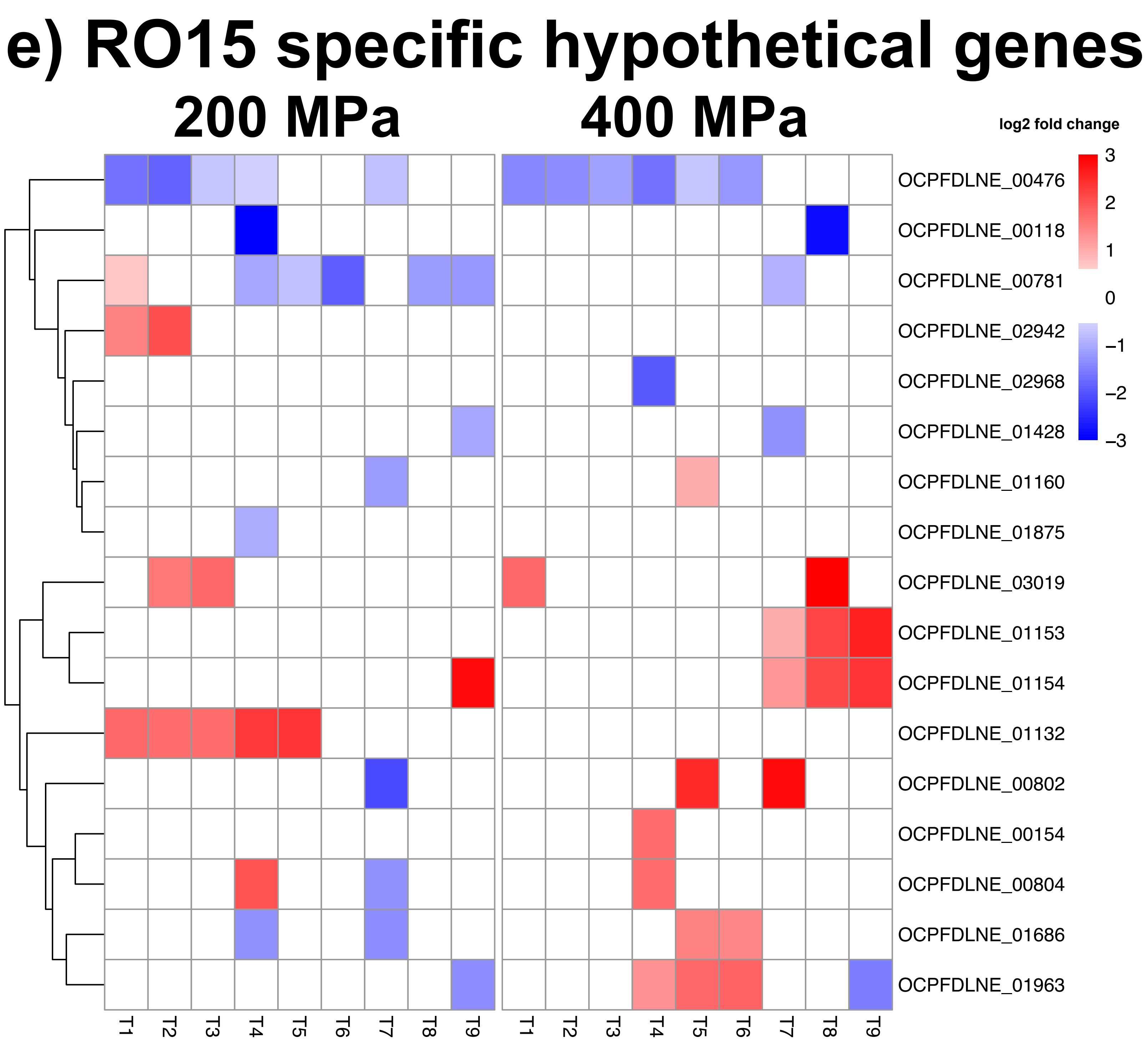
e) RO15 specific hypothetical genes
200 MPa
400 MPa
log2 fold change
3
2
1
0
−1
−2
−3
OCPFDLNE_00476
OCPFDLNE_00118
OCPFDLNE_00781
OCPFDLNE_02942
OCPFDLNE_02968
OCPFDLNE_01428
OCPFDLNE_01160
OCPFDLNE_01875
OCPFDLNE_03019
OCPFDLNE_01153
OCPFDLNE_01154
OCPFDLNE_01132
OCPFDLNE_00802
OCPFDLNE_00154
OCPFDLNE_00804
OCPFDLNE_01686
OCPFDLNE_01963
T1
T2
T3
T4
T5
T6
T7
T8
T9
T1
T2
T3
T4
T5
T6
T7
T8
T9

f) ScottA specific hypothetical genes

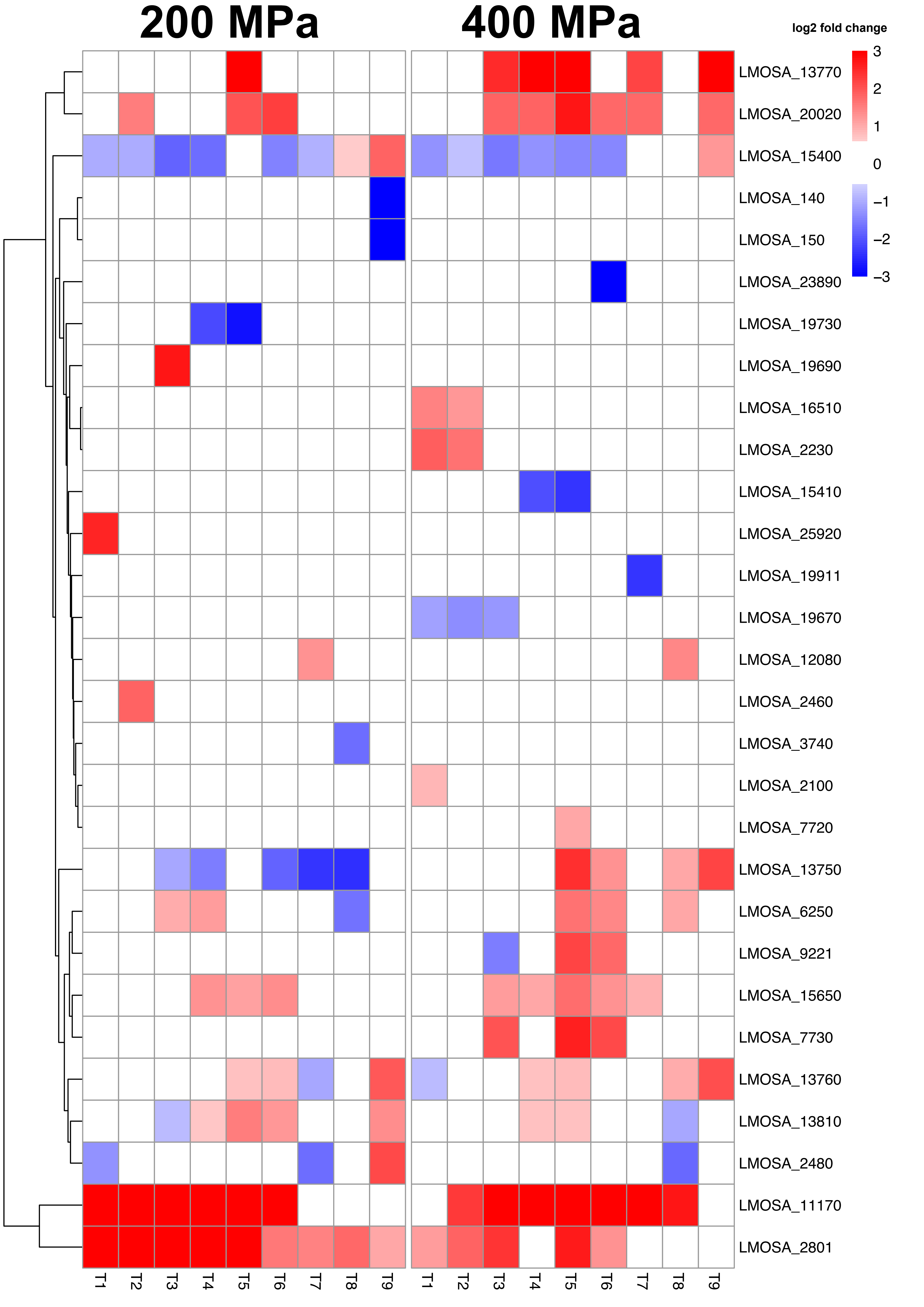

Supplement: Supplementary file 13 — Additional file 13: Figure S13. Log2 fold change heatmap of hypothetical/uncharacterized genes of RO15 and ScottA. [file 12864_2021_7407_MOESM13_ESM.pdf]
