# Supplementary material for: High-pressure processing-induced transcriptome response during recovery of Listeria monocytogenes
Source: BMC Genomics. 2021 Feb 12;22:117. doi: 10.1186/s12864-021-07407-6 (PMC7881616; doi:10.1186/s12864-021-07407-6)
Supplement: Supplementary file 15 — Additional file 15: Figure S15. Visualization of amino acid sequence similarity of lmo1013 and lmo2229 genes in strain EGDe, RO15, and ScottA. [file 12864_2021_7407_MOESM15_ESM.pdf]

## *lmo1013* vs LMOSA_19040 (ScottA) BLASTP alignment

```
Query= EGJ24516.1 [locustag:LMOSA_19040]

Length=293
                                                                      Score     E
Sequences producing significant alignments:                          (Bits)  Value

lmo1013                                                               566     0.0


>lmo1013
Length=281

 Score = 566 bits (1460),  Expect = 0.0, Method: Compositional matrix adjust.
 Identities = 279/281 (99%), Positives = 281/281 (100%), Gaps = 0/281 (0%)

Query  13   MNLLKKWFSSIDWDQFWNHIISVGIKIAILIALYFIFRVVGNKIIRSFFRKYRQQQAVSV  72
            MNLLKKWFSSIDWDQFWNHIISVGIKIAILIALYFIFRV+GNKIIRSFFRKYRQQQAVSV
Sbjct  1    MNLLKKWFSSIDWDQFWNHIISVGIKIAILIALYFIFRVIGNKIIRSFFRKYRQQQAVSV  60

Query  73   GRADTLESLISNFYGYVLFFTFAILLLQNFMDVTAIIASAGVASLAIAFGAQGLVSDVVT  132
            GRADTLESLISNFYGYVLFFTFAILLLQNFMDVTAIIASAGVASLAIAFGAQGLVSDVVT
Sbjct  61   GRADTLESLISNFYGYVLFFTFAILLLQNFMDVTAIIASAGVASLAIAFGAQGLVSDVVT  120

Query  133  GFFILLERQLDVGDTITIGLVNGTVEALGLRTTQVRDFDGTLHFIPNRQIMVVSNHSRGN  192
            GFFILLERQLDVGDTITIGLVNGTVEALGLRTTQVRDFDGTLHFIPNRQIMVVSNHSRGN
Sbjct  121  GFFILLERQLDVGDTITIGLVNGTVEALGLRTTQVRDFDGTLHFIPNRQIMVVSNHSRGN  180

Query  193  MRVMVDIQISPHEDPEKAIKIIGEVCDVAAKENKNIVEPPVVLGVQNIDATNMIIRVVGK  252
            MRVMVDIQISPHEDPEKAIKIIGEVCDVAAKENKNIVEPPVVLGVQNIDATNMIIRVVGK
Sbjct  181  MRVMVDIQISPHEDPEKAIKIIGEVCDVAAKENKNIVEPPVVLGVQNIDATNMIIRVVGK  240

Query  253  AVNGEQYSVQRDLLKDIREALAENEIELPLNFVSTFGPNNN  293
            AVNGEQYSVQRDLLKDIREALAEN+IELPLNFVSTFGPNNN
Sbjct  241  AVNGEQYSVQRDLLKDIREALAENKIELPLNFVSTFGPNNN  281
```

## *lmo1013* vs OCPFDLNE_01076 (RO15) BLASTP alignment

```
Query= OCPFDLNE_01076

Length=293
                                                                      Score     E
Sequences producing significant alignments:                          (Bits)  Value

lmo1013                                                               565     0.0


>lmo1013
Length=281

 Score = 565 bits (1455),  Expect = 0.0, Method: Compositional matrix adjust.
 Identities = 279/281 (99%), Positives = 280/281 (99%), Gaps = 0/281 (0%)

Query  13   MNLLKKWFSSIDWDQFWNHIISVGIKIAILIALYFIFRVIGNKIIRSFFRKYRQQQAVSV  72
            MNLLKKWFSSIDWDQFWNHIISVGIKIAILIALYFIFRVIGNKIIRSFFRKYRQQQAVSV
Sbjct  1    MNLLKKWFSSIDWDQFWNHIISVGIKIAILIALYFIFRVIGNKIIRSFFRKYRQQQAVSV  60

Query  73   GRADTLESLISNFYGYVLFFTFAILLLQNFMDVTAIIASAGVASLAIAFGAQGLVSDVVT  132
            GRADTLESLISNFYGYVLFFTFAILLLQNFMDVTAIIASAGVASLAIAFGAQGLVSDVVT
Sbjct  61   GRADTLESLISNFYGYVLFFTFAILLLQNFMDVTAIIASAGVASLAIAFGAQGLVSDVVT  120

Query  133  GFFILLERQLDVGDTITIGLVNGTVEALGLRTTLVRDFDGTLHFIPNRQIMVVSNHSRGN  192
            GFFILLERQLDVGDTITIGLVNGTVEALGLRTT VRDFDGTLHFIPNRQIMVVSNHSRGN
Sbjct  121  GFFILLERQLDVGDTITIGLVNGTVEALGLRTTQVRDFDGTLHFIPNRQIMVVSNHSRGN  180

Query  193  MRVMVDIQISPHEDPEKAIKIIGEVCDIAAKENKNIVEPPVVLGVQNIDATNMIIRVVGK  252
            MRVMVDIQISPHEDPEKAIKIIGEVCD+AAKENKNIVEPPVVLGVQNIDATNMIIRVVGK
Sbjct  181  MRVMVDIQISPHEDPEKAIKIIGEVCDVAAKENKNIVEPPVVLGVQNIDATNMIIRVVGK  240

Query  253  AVNGEQYSVQRDLLKDIREALAENKIELPLNFVSTFGPNNN  293
            AVNGEQYSVQRDLLKDIREALAENKIELPLNFVSTFGPNNN
Sbjct  241  AVNGEQYSVQRDLLKDIREALAENKIELPLNFVSTFGPNNN  281
```

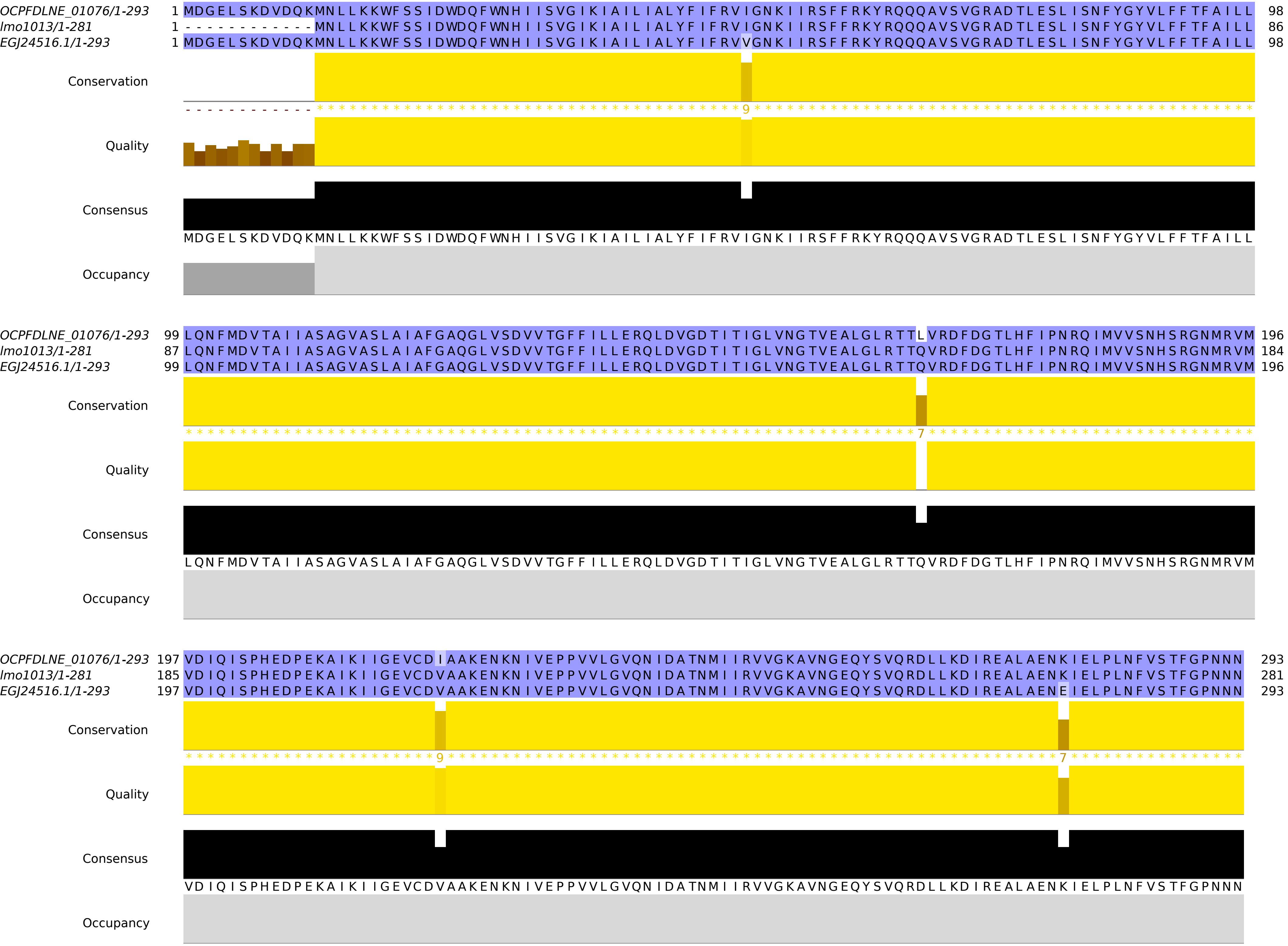

OCPFDLNE_01076/1-293 1 MDGELSKDVDQKMNLLKKWFSSIDWDQFWNHIISVGIKIAILIALYFIFRVIGNKIIRSFFRKYRQQQAVSVGRADTLESLISNFYGYVLFFTFAILL 98
lmo1013/1-281 1 ------------MNLLKKWFSSIDWDQFWNHIISVGIKIAILIALYFIFRVIGNKIIRSFFRKYRQQQAVSVGRADTLESLISNFYGYVLFFTFAILL 86
EGJ24516.1/1-293 1 MDGELSKDVDQKMNLLKKWFSSIDWDQFWNHIISVGIKIAILIALYFIFRVVGNKIIRSFFRKYRQQQAVSVGRADTLESLISNFYGYVLFFTFAILL 98
Conservation
Quality
Consensus
MDGELSKDVDQKMNLLKKWFSSIDWDQFWNHIISVGIKIAILIALYFIFRVIGNKIIRSFFRKYRQQQAVSVGRADTLESLISNFYGYVLFFTFAILL
Occupancy
OCPFDLNE_01076/1-293 99 LQNFMDVTAIIASAGVASLAIAFGAQGLVSDVVTGFFILLERQLDVGDTITIGLVNGTVEALGLRTTLVRDFDGTLHFIPNRQIMVVSNHSRGNMRVM 196
lmo1013/1-281 87 LQNFMDVTAIIASAGVASLAIAFGAQGLVSDVVTGFFILLERQLDVGDTITIGLVNGTVEALGLRTTQVRDFDGTLHFIPNRQIMVVSNHSRGNMRVM 184
EGJ24516.1/1-293 99 LQNFMDVTAIIASAGVASLAIAFGAQGLVSDVVTGFFILLERQLDVGDTITIGLVNGTVEALGLRTTQVRDFDGTLHFIPNRQIMVVSNHSRGNMRVM 196
Conservation
Quality
Consensus
LQNFMDVTAIIASAGVASLAIAFGAQGLVSDVVTGFFILLERQLDVGDTITIGLVNGTVEALGLRTTQVRDFDGTLHFIPNRQIMVVSNHSRGNMRVM
Occupancy
OCPFDLNE_01076/1-293 197 VDIQISPHEDPEKAIKIIGEVCDIAAKENKNIVEPPVVLGVQNIDATNMIIRVVGKAVNGEQYSVQRDLLKDIREALAENKIELPLNFVSTFGPNNN 293
lmo1013/1-281 185 VDIQISPHEDPEKAIKIIGEVCDVAAKENKNIVEPPVVLGVQNIDATNMIIRVVGKAVNGEQYSVQRDLLKDIREALAENKIELPLNFVSTFGPNNN 281
EGJ24516.1/1-293 197 VDIQISPHEDPEKAIKIIGEVCDVAAKENKNIVEPPVVLGVQNIDATNMIIRVVGKAVNGEQYSVQRDLLKDIREALAENEIELPLNFVSTFGPNNN 293
Conservation
Quality
Consensus
VDIQISPHEDPEKAIKIIGEVCDVAAKENKNIVEPPVVLGVQNIDATNMIIRVVGKAVNGEQYSVQRDLLKDIREALAENKIELPLNFVSTFGPNNN
Occupancy

## *lmo2229* vs LMOSA_2530 (ScottA) BLASTP alignment

```
>lmo2229
Length=714

 Score = 1447 bits (3746),  Expect = 0.0, Method: Compositional matrix adjust.
 Identities = 711/714 (99%), Positives = 712/714 (99%), Gaps = 2/714 (0%)

Query  1    MDKFKQQLIKYLKLFFGFIGKWLSKGWQKFRRFWKNKHIGKIFLLAGLVFLLSFIIYLVI  60
            MDKFKQQLIKYLKLFFGFIGKWL+KGWQKFRRFWKNKHIGKIFLLAGLVFLLSFIIYLVI
Sbjct  1    MDKFKQQLIKYLKLFFGFIGKWLNKGWQKFRRFWKNKHIGKIFLLAGLVFLLSFIIYLVI  60

Query  61   VAKSADIDALKKGLESATIIYDKDGDKAGELSSTDATFVSIDKISKNLQNAVVSIEDRKF  120
            VAKSADIDALKKGLESATIIYDKDGDKAGELSSTDATFVSIDKISKNLQNAVVSIEDRKF
Sbjct  61   VAKSADIDALKKGLESATIIYDKDGDKAGELSSTDATFVSIDKISKNLQNAVVSIEDRKF  120

Query  121  YEHKGFDLKGIARAGVNLVTSGGISGGGSTITQQLAKNALLTQEQTFTRKAKEIFMAREI  180
            YEHKGFDLKGIARAGVNLVTSGGISGGGSTITQQLAKNALLTQEQTFTRKAKEIFMAREI
Sbjct  121  YEHKGFDLKGIARAGVNLVTSGGISGGGSTITQQLAKNALLTQEQTFTRKAKEIFMAREI  180

Query  181  EKTYSKDEIMEMYLNRSYFGNGEWGVENASLKYFGKSAADLNIPEAATIAGLLQAPSAYD  240
            EKTYSKDEIMEMYLNRSYFGNGEWGVENASLKYFGKSAADLNIPEAATIAGLLQAPSAYD
Sbjct  181  EKTYSKDEIMEMYLNRSYFGNGEWGVENASLKYFGKSAADLNIPEAATIAGLLQAPSAYD  240

Query  241  PYQHIDKATNRRNMVLNAMVETGTISKAEGDKYKATKIVLNDQSKDPLANKYPWYVDAVI  300
            PYQHIDKATNRRNMVLNAMVETGTISKAEGDKYKATKIVLNDQSKDPLANKYPWYVDAVI
Sbjct  241  PYQHIDKATNRRNMVLNAMVETGTISKAEGDKYKATKIVLNDQSKDPLANKYPWYVDAVI  300

Query  301  NEAVNEADITQDEIMQKGYKIYTELDQNYQTSLENVYNNDGLFPSNANDGTLVQSGAVLM  360
            NEAVNEADITQDEIMQKGYKIYTELDQNYQTSLENVYNNDGLFPSNANDGTLVQSGAVLM
Sbjct  301  NEAVNEADITQDEIMQKGYKIYTELDQNYQTSLENVYNNDGLFPSNANDGTLVQSGAVLM  360

Query  361  DPATGGIRALVGGRGEHVFRGFNRATQMKAQPGSTMKPLAVYTPALQSGYDVDSMLKDEK  420
            DPATGGIRALVGGRGEHVFRGFNRATQMKAQPGSTMKPLAVYTPALQSGYDVDSMLKDEK
Sbjct  361  DPATGGIRALVGGRGEHVFRGFNRATQMKAQPGSTMKPLAVYTPALQSGYDVDSMLKDEK  420

Query  421  ITYKGNYTPTNVGGVYSGEVPMYKAVANSINAPAVWLLDQIGIDKGVKSVEKFGITVPEK  480
            ITYKGNYTPTNVGGVYSGEVPMYKAVANSINAPAVWLLDQIGIDKGVKSVEKFGITVPEK
Sbjct  421  ITYKGNYTPTNVGGVYSGEVPMYKAVANSINAPAVWLLDQIGIDKGVKSVEKFGITVPEK  480

Query  481  DRTLGLALGGMSKGASPVEMATAYATFANNGAKPESHIITKIVDPSGNTVYENVPKTKQI  540
            DRTLGLALGGMSKGASPVEMATAYATFANNGAKPESHIITKIVDPSGNTVYENVPKTKQI
Sbjct  481  DRTLGLALGGMSKGASPVEMATAYATFANNGAKPESHIITKIVDPSGNTVYENVPKTKQI  540

Query  541  ISETVSNEMTSMLLDVINTGTGQSAAVSGHEMAGKTGSTQVPFDDTSGTKDQWFVGYTPN  600
            ISETVSNEMTSMLLDVINTGTGQSAAVSGHEMAGKTGSTQVPFDDTSGTKDQWFVGYTPN
Sbjct  541  ISETVSNEMTSMLLDVINTGTGQSAAVSGHEMAGKTGSTQVPFDDTSGTKDQWFVGYTPN  600

Query  601  LVGAVWMGYDKTDKEHYLTTTSSAGVSSLAHYVMNSGLQYQKSADFSTKSAAQETAAKK-  659
            LVGAVWMGYDKTDKEHYLTTTSSAGVSSLAHYVMNSGLQYQKSADFSTKSAAQETAAKK
Sbjct  601  LVGAVWMGYDKTDKEHYLTTTSSAGVSSLAHYVMNSGLQYQKSADFSTKSAAQETAAKKE  660

Query  660  -EEKEKNSGSDFWSGVKEKADEAGETIKKGADKVKEFGGKVSDGIGNLIDSIGN  712
             EEKEKNSGSDFWSGVKEKADEAGETIKKGADKVKEFGGKVSDGIGNLIDSIGN
Sbjct  661  EEEKEKNSGSDFWSGVKEKADEAGETIKKGADKVKEFGGKVSDGIGNLIDSIGN  714
```

## *lmo2229* vs OCPFDLNE_02389 (RO15) BLASTP alignment

```
>lmo2229
Length=714

 Score = 1457 bits (3772),  Expect = 0.0, Method: Compositional matrix adjust.
 Identities = 714/714 (100%), Positives = 714/714 (100%), Gaps = 0/714 (0%)

Query  1    MDKFKQQLIKYLKLFFGFIGKWLNKGWQKFRRFWKNKHIGKIFLLAGLVFLLSFIIYLVI  60
            MDKFKQQLIKYLKLFFGFIGKWLNKGWQKFRRFWKNKHIGKIFLLAGLVFLLSFIIYLVI
Sbjct  1    MDKFKQQLIKYLKLFFGFIGKWLNKGWQKFRRFWKNKHIGKIFLLAGLVFLLSFIIYLVI  60

Query  61   VAKSADIDALKKGLESATIIYDKDGDKAGELSSTDATFVSIDKISKNLQNAVVSIEDRKF  120
            VAKSADIDALKKGLESATIIYDKDGDKAGELSSTDATFVSIDKISKNLQNAVVSIEDRKF
Sbjct  61   VAKSADIDALKKGLESATIIYDKDGDKAGELSSTDATFVSIDKISKNLQNAVVSIEDRKF  120

Query  121  YEHKGFDLKGIARAGVNLVTSGGISGGGSTITQQLAKNALLTQEQTFTRKAKEIFMAREI  180
            YEHKGFDLKGIARAGVNLVTSGGISGGGSTITQQLAKNALLTQEQTFTRKAKEIFMAREI
Sbjct  121  YEHKGFDLKGIARAGVNLVTSGGISGGGSTITQQLAKNALLTQEQTFTRKAKEIFMAREI  180

Query  181  EKTYSKDEIMEMYLNRSYFGNGEWGVENASLKYFGKSAADLNIPEAATIAGLLQAPSAYD  240
            EKTYSKDEIMEMYLNRSYFGNGEWGVENASLKYFGKSAADLNIPEAATIAGLLQAPSAYD
Sbjct  181  EKTYSKDEIMEMYLNRSYFGNGEWGVENASLKYFGKSAADLNIPEAATIAGLLQAPSAYD  240

Query  241  PYQHIDKATNRRNMVLNAMVETGTISKAEGDKYKATKIVLNDQSKDPLANKYPWYVDAVI  300
            PYQHIDKATNRRNMVLNAMVETGTISKAEGDKYKATKIVLNDQSKDPLANKYPWYVDAVI
Sbjct  241  PYQHIDKATNRRNMVLNAMVETGTISKAEGDKYKATKIVLNDQSKDPLANKYPWYVDAVI  300

Query  301  NEAVNEADITQDEIMQKGYKIYTELDQNYQTSLENVYNNDGLFPSNANDGTLVQSGAVLM  360
            NEAVNEADITQDEIMQKGYKIYTELDQNYQTSLENVYNNDGLFPSNANDGTLVQSGAVLM
Sbjct  301  NEAVNEADITQDEIMQKGYKIYTELDQNYQTSLENVYNNDGLFPSNANDGTLVQSGAVLM  360

Query  361  DPATGGIRALVGGRGEHVFRGFNRATQMKAQPGSTMKPLAVYTPALQSGYDVDSMLKDEK  420
            DPATGGIRALVGGRGEHVFRGFNRATQMKAQPGSTMKPLAVYTPALQSGYDVDSMLKDEK
Sbjct  361  DPATGGIRALVGGRGEHVFRGFNRATQMKAQPGSTMKPLAVYTPALQSGYDVDSMLKDEK  420

Query  421  ITYKGNYTPTNVGGVYSGEVPMYKAVANSINAPAVWLLDQIGIDKGVKSVEKFGITVPEK  480
            ITYKGNYTPTNVGGVYSGEVPMYKAVANSINAPAVWLLDQIGIDKGVKSVEKFGITVPEK
Sbjct  421  ITYKGNYTPTNVGGVYSGEVPMYKAVANSINAPAVWLLDQIGIDKGVKSVEKFGITVPEK  480

Query  481  DRTLGLALGGMSKGASPVEMATAYATFANNGAKPESHIITKIVDPSGNTVYENVPKTKQI  540
            DRTLGLALGGMSKGASPVEMATAYATFANNGAKPESHIITKIVDPSGNTVYENVPKTKQI
Sbjct  481  DRTLGLALGGMSKGASPVEMATAYATFANNGAKPESHIITKIVDPSGNTVYENVPKTKQI  540

Query  541  ISETVSNEMTSMLLDVINTGTGQSAAVSGHEMAGKTGSTQVPFDDTSGTKDQWFVGYTPN  600
            ISETVSNEMTSMLLDVINTGTGQSAAVSGHEMAGKTGSTQVPFDDTSGTKDQWFVGYTPN
Sbjct  541  ISETVSNEMTSMLLDVINTGTGQSAAVSGHEMAGKTGSTQVPFDDTSGTKDQWFVGYTPN  600

Query  601  LVGAVWMGYDKTDKEHYLTTTSSAGVSSLAHYVMNSGLQYQKSADFSTKSAAQETAAKKE  660
            LVGAVWMGYDKTDKEHYLTTTSSAGVSSLAHYVMNSGLQYQKSADFSTKSAAQETAAKKE
Sbjct  601  LVGAVWMGYDKTDKEHYLTTTSSAGVSSLAHYVMNSGLQYQKSADFSTKSAAQETAAKKE  660

Query  661  EEEKEKNSGSDFWSGVKEKADEAGETIKKGADKVKEFGGKVSDGIGNLIDSIGN  714
            EEEKEKNSGSDFWSGVKEKADEAGETIKKGADKVKEFGGKVSDGIGNLIDSIGN
Sbjct  661  EEEKEKNSGSDFWSGVKEKADEAGETIKKGADKVKEFGGKVSDGIGNLIDSIGN  714
```

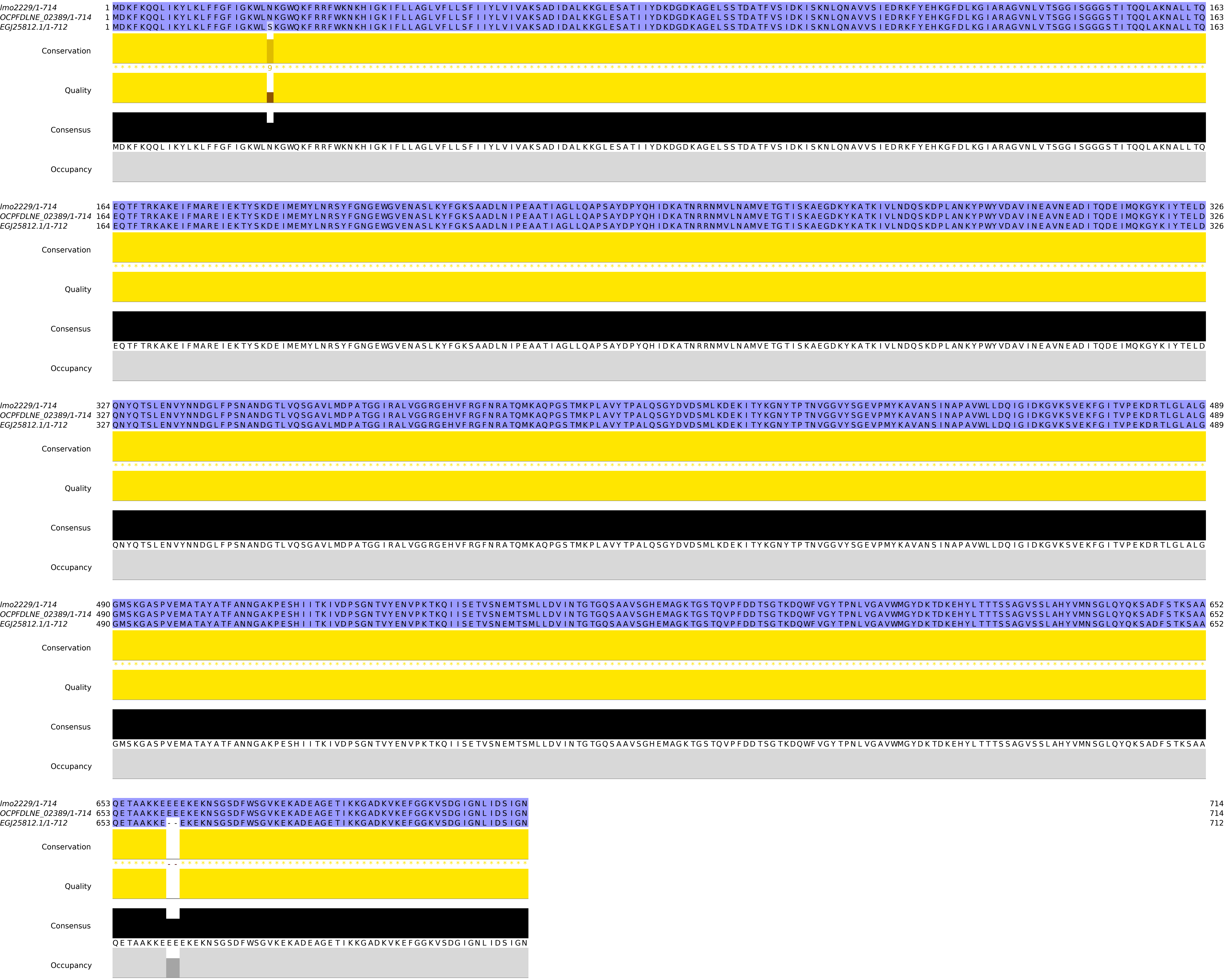
lmo2229/1-714 1 MDKFKQQLIKYLKLFFGFIGKWLNKGWQKFRRFWKNKHIGKIFLLAGLVFLLSFIIYLVIVAKSADIDALKKGLESATIIYDKDGDKAGELSSTDATFVSIDKISKNLQNAVVSIEDRKFYEHKGFDLKGIARAGVNLVTSGGISGGGSTITQQLAKNALLTQ 163
OCPFDLNE_02389/1-714 1 MDKFKQQLIKYLKLFFGFIGKWLNKGWQKFRRFWKNKHIGKIFLLAGLVFLLSFIIYLVIVAKSADIDALKKGLESATIIYDKDGDKAGELSSTDATFVSIDKISKNLQNAVVSIEDRKFYEHKGFDLKGIARAGVNLVTSGGISGGGSTITQQLAKNALLTQ 163
EGJ25812.1/1-712 1 MDKFKQQLIKYLKLFFGFIGKWLSKGWQKFRRFWKNKHIGKIFLLAGLVFLLSFIIYLVIVAKSADIDALKKGLESATIIYDKDGDKAGELSSTDATFVSIDKISKNLQNAVVSIEDRKFYEHKGFDLKGIARAGVNLVTSGGISGGGSTITQQLAKNALLTQ 163
Conservation
9
Quality
Consensus
MDKFKQQLIKYLKLFFGFIGKWLNKGWQKFRRFWKNKHIGKIFLLAGLVFLLSFIIYLVIVAKSADIDALKKGLESATIIYDKDGDKAGELSSTDATFVSIDKISKNLQNAVVSIEDRKFYEHKGFDLKGIARAGVNLVTSGGISGGGSTITQQLAKNALLTQ
Occupancy
lmo2229/1-714 164 EQTFTRKAKEIFMAREIEKTYSKDEIMEMYLNRSYFGNGEWGVENASLKYFGKSAADLNIPEAATIAGLLQAPSAYDPYQHIDKATNRRNMVLNAMVETGTISKAEGDKYKATKIVLNDQSKDPLANKYPWYVDAVINEAVNEADITQDEIMQKGYKIYTELD 326
OCPFDLNE_02389/1-714 164 EQTFTRKAKEIFMAREIEKTYSKDEIMEMYLNRSYFGNGEWGVENASLKYFGKSAADLNIPEAATIAGLLQAPSAYDPYQHIDKATNRRNMVLNAMVETGTISKAEGDKYKATKIVLNDQSKDPLANKYPWYVDAVINEAVNEADITQDEIMQKGYKIYTELD 326
EGJ25812.1/1-712 164 EQTFTRKAKEIFMAREIEKTYSKDEIMEMYLNRSYFGNGEWGVENASLKYFGKSAADLNIPEAATIAGLLQAPSAYDPYQHIDKATNRRNMVLNAMVETGTISKAEGDKYKATKIVLNDQSKDPLANKYPWYVDAVINEAVNEADITQDEIMQKGYKIYTELD 326
Conservation
Quality
Consensus
EQTFTRKAKEIFMAREIEKTYSKDEIMEMYLNRSYFGNGEWGVENASLKYFGKSAADLNIPEAATIAGLLQAPSAYDPYQHIDKATNRRNMVLNAMVETGTISKAEGDKYKATKIVLNDQSKDPLANKYPWYVDAVINEAVNEADITQDEIMQKGYKIYTELD
Occupancy
lmo2229/1-714 327 QNYQTSLENVYNNDGLFPSNANDGTLVQSGAVLMDPATGGIRALVGGRGEHVFRGFNRATQMKAQPGSTMKPLAVYTPALQSGYDVDSMLKDEKITYKGNYTPTNVGGVYSGEVPMYKAVANSINAPAVWLLDQIGIDKGVKSVEKFGITVPEKDRTLGLALG 489
OCPFDLNE_02389/1-714 327 QNYQTSLENVYNNDGLFPSNANDGTLVQSGAVLMDPATGGIRALVGGRGEHVFRGFNRATQMKAQPGSTMKPLAVYTPALQSGYDVDSMLKDEKITYKGNYTPTNVGGVYSGEVPMYKAVANSINAPAVWLLDQIGIDKGVKSVEKFGITVPEKDRTLGLALG 489
EGJ25812.1/1-712 327 QNYQTSLENVYNNDGLFPSNANDGTLVQSGAVLMDPATGGIRALVGGRGEHVFRGFNRATQMKAQPGSTMKPLAVYTPALQSGYDVDSMLKDEKITYKGNYTPTNVGGVYSGEVPMYKAVANSINAPAVWLLDQIGIDKGVKSVEKFGITVPEKDRTLGLALG 489
Conservation
Quality
Consensus
QNYQTSLENVYNNDGLFPSNANDGTLVQSGAVLMDPATGGIRALVGGRGEHVFRGFNRATQMKAQPGSTMKPLAVYTPALQSGYDVDSMLKDEKITYKGNYTPTNVGGVYSGEVPMYKAVANSINAPAVWLLDQIGIDKGVKSVEKFGITVPEKDRTLGLALG
Occupancy
lmo2229/1-714 490 GMSKGASPVEMATAYATFANNGAKPESHIITKIVDPSGNTVYENVPKTKQIISETVSNEMTSMLLDVINTGTGQSAAVSGHEMAGKTGSTQVPFDDTSGTKDQWFVGYTPNLVGAVWMGYDKTDKEHYLTTTSSAGVSSLAHYVMNSGLQYQKSADFSTKSAA 652
OCPFDLNE_02389/1-714 490 GMSKGASPVEMATAYATFANNGAKPESHIITKIVDPSGNTVYENVPKTKQIISETVSNEMTSMLLDVINTGTGQSAAVSGHEMAGKTGSTQVPFDDTSGTKDQWFVGYTPNLVGAVWMGYDKTDKEHYLTTTSSAGVSSLAHYVMNSGLQYQKSADFSTKSAA 652
EGJ25812.1/1-712 490 GMSKGASPVEMATAYATFANNGAKPESHIITKIVDPSGNTVYENVPKTKQIISETVSNEMTSMLLDVINTGTGQSAAVSGHEMAGKTGSTQVPFDDTSGTKDQWFVGYTPNLVGAVWMGYDKTDKEHYLTTTSSAGVSSLAHYVMNSGLQYQKSADFSTKSAA 652
Conservation
Quality
Consensus
GMSKGASPVEMATAYATFANNGAKPESHIITKIVDPSGNTVYENVPKTKQIISETVSNEMTSMLLDVINTGTGQSAAVSGHEMAGKTGSTQVPFDDTSGTKDQWFVGYTPNLVGAVWMGYDKTDKEHYLTTTSSAGVSSLAHYVMNSGLQYQKSADFSTKSAA
Occupancy
lmo2229/1-714 653 QETAAKKEEEEEKEKNSGSDFWSGVKEKADEAGETIKKGADKVKEFGGKVSDGIGNLIDSIGN 714
OCPFDLNE_02389/1-714 653 QETAAKKEEEEEKEKNSGSDFWSGVKEKADEAGETIKKGADKVKEFGGKVSDGIGNLIDSIGN 714
EGJ25812.1/1-712 653 QETAAKKE--EKEKNSGSDFWSGVKEKADEAGETIKKGADKVKEFGGKVSDGIGNLIDSIGN 712
Conservation
Quality
Consensus
QETAAKKEEEEEKEKNSGSDFWSGVKEKADEAGETIKKGADKVKEFGGKVSDGIGNLIDSIGN
Occupancy
