# Supplementary material for: High-pressure processing-induced transcriptome response during recovery of Listeria monocytogenes
Source: BMC Genomics. 2021 Feb 12;22:117. doi: 10.1186/s12864-021-07407-6 (PMC7881616; doi:10.1186/s12864-021-07407-6)
Supplement: Supplementary file 16 — Additional file 16: Figure S16. Log2 fold change heatmap of all differentially expressed genes in both strains. The rows were clustered based on log2 fold change. Clusters that show strain-specific differences were shown in detail. [file 12864_2021_7407_MOESM16_ESM.pdf]

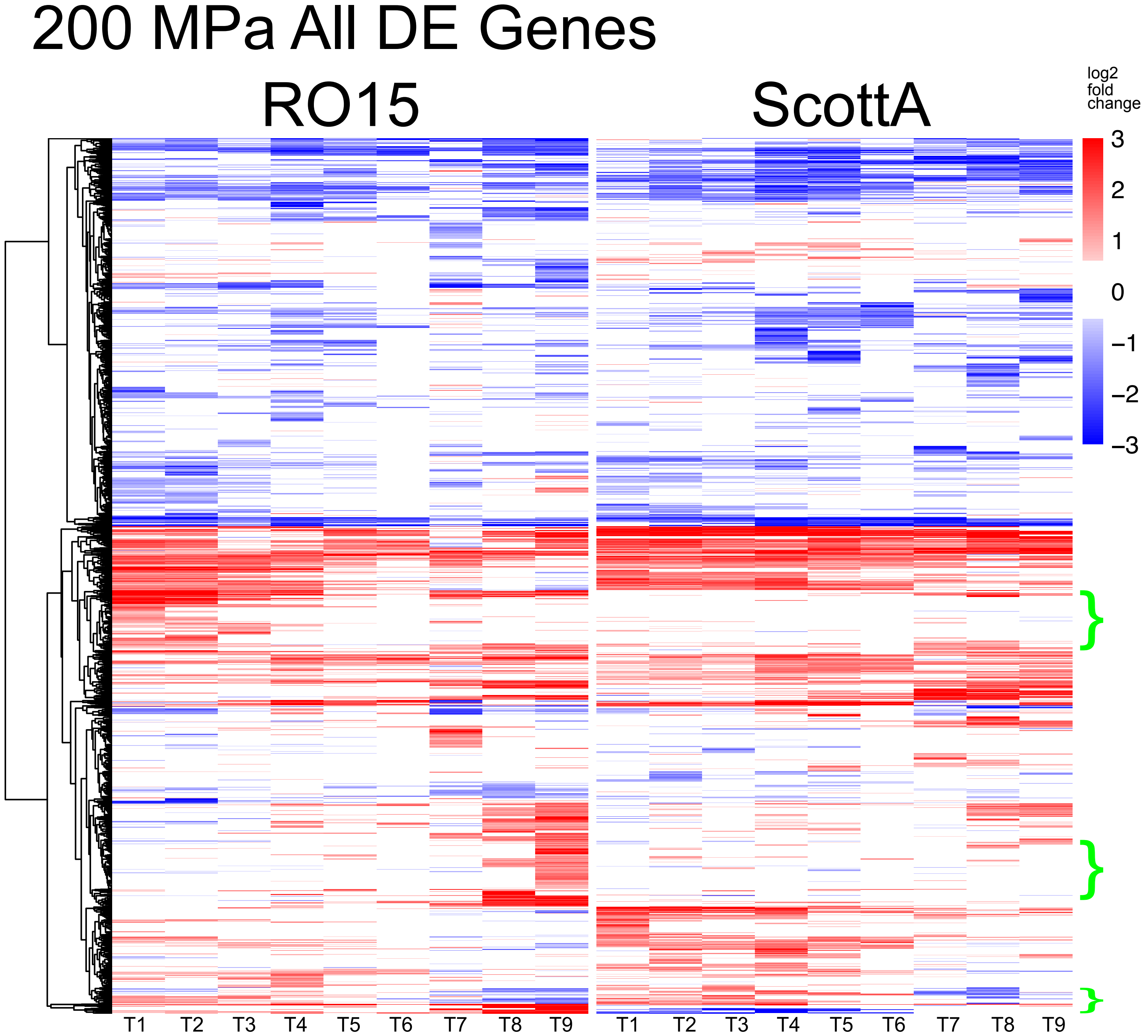
200 MPa All DE Genes
RO15
ScottA
log2 fold change
3
2
1
0
−1
−2
−3
T1
T2
T3
T4
T5
T6
T7
T8
T9
T1
T2
T3
T4
T5
T6
T7
T8
T9

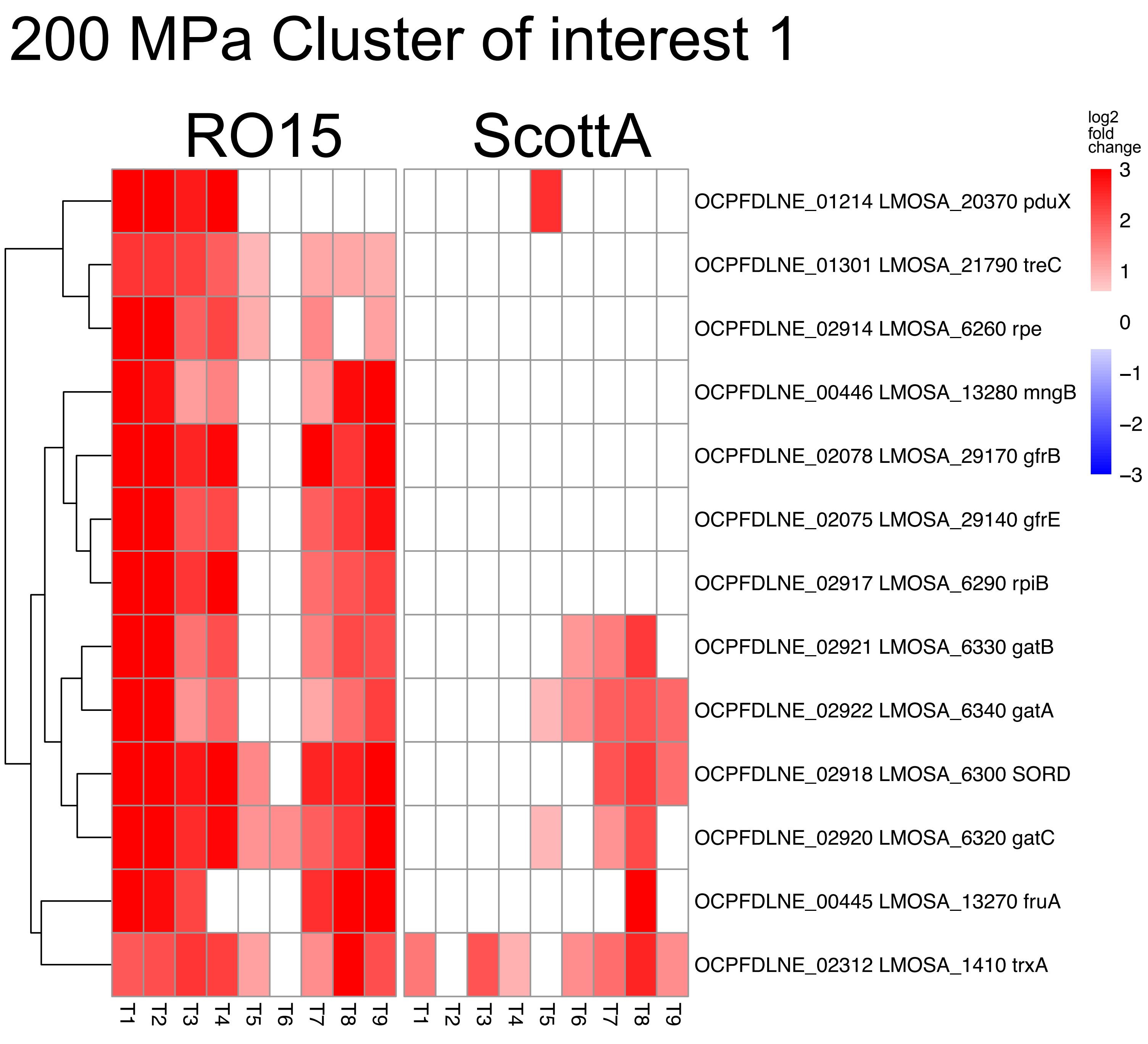
200 MPa Cluster of interest 1
RO15
ScottA
log2 fold change
3
2
1
0
−1
−2
−3
OCPFDLNE_01214 LMOSA_20370 pduX
OCPFDLNE_01301 LMOSA_21790 treC
OCPFDLNE_02914 LMOSA_6260 rpe
OCPFDLNE_00446 LMOSA_13280 mngB
OCPFDLNE_02078 LMOSA_29170 gfrB
OCPFDLNE_02075 LMOSA_29140 gfrE
OCPFDLNE_02917 LMOSA_6290 rpiB
OCPFDLNE_02921 LMOSA_6330 gatB
OCPFDLNE_02922 LMOSA_6340 gatA
OCPFDLNE_02918 LMOSA_6300 SORD
OCPFDLNE_02920 LMOSA_6320 gatC
OCPFDLNE_00445 LMOSA_13270 fruA
OCPFDLNE_02312 LMOSA_1410 trxA
T1
T2
T3
T4
T5
T6
T7
T8
T9
T1
T2
T3
T4
T5
T6
T7
T8
T9

200 MPa Cluster of interest 2

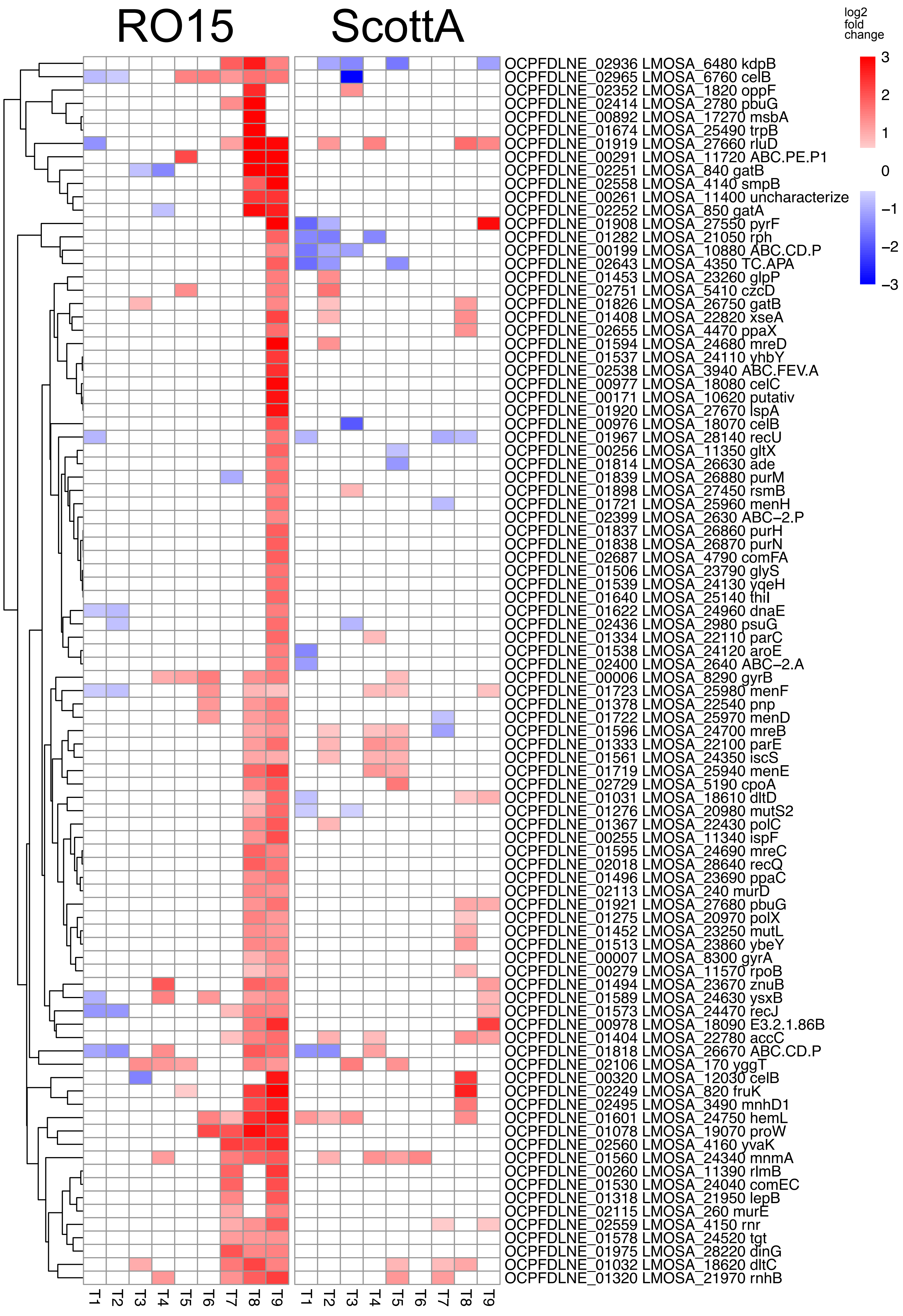

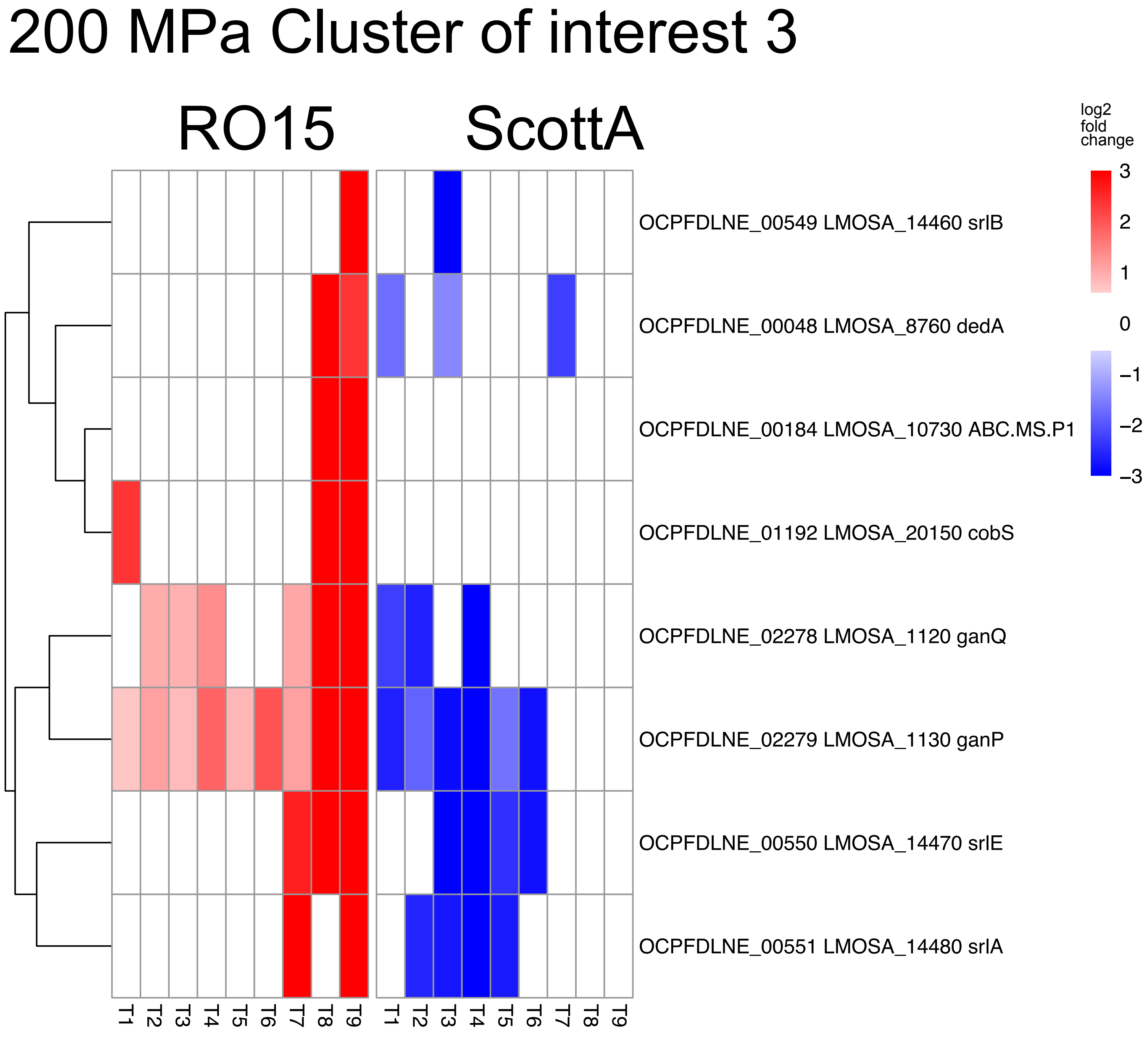
200 MPa Cluster of interest 3
RO15
ScottA
log2 fold change
3
2
1
0
−1
−2
−3
OCPFDLNE_00549 LMOSA_14460 srlB
OCPFDLNE_00048 LMOSA_8760 dedA
OCPFDLNE_00184 LMOSA_10730 ABC.MS.P1
OCPFDLNE_01192 LMOSA_20150 cobS
OCPFDLNE_02278 LMOSA_1120 ganQ
OCPFDLNE_02279 LMOSA_1130 ganP
OCPFDLNE_00550 LMOSA_14470 srlE
OCPFDLNE_00551 LMOSA_14480 srlA
T1
T2
T3
T4
T5
T6
T7
T8
T9
T1
T2
T3
T4
T5
T6
T7
T8
T9

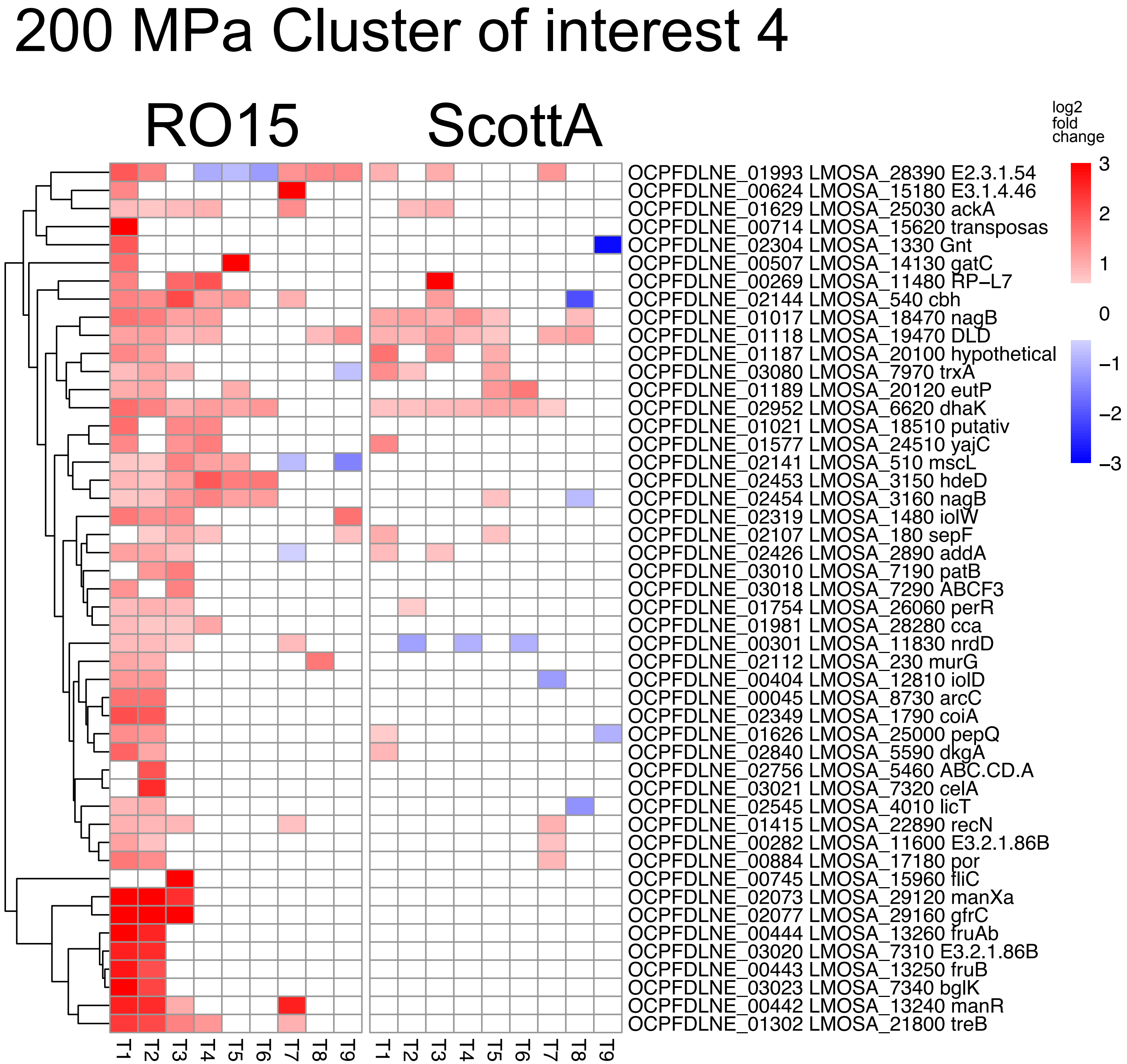
200 MPa Cluster of interest 4
RO15
ScottA
log2 fold change
3
2
1
0
−1
−2
−3
OCPFDLNE_01993 LMOSA_28390 E2.3.1.54
OCPFDLNE_00624 LMOSA_15180 E3.1.4.46
OCPFDLNE_01629 LMOSA_25030 ackA
OCPFDLNE_00714 LMOSA_15620 transposas
OCPFDLNE_02304 LMOSA_1330 Gnt
OCPFDLNE_00507 LMOSA_14130 gatC
OCPFDLNE_00269 LMOSA_11480 RP−L7
OCPFDLNE_02144 LMOSA_540 cbh
OCPFDLNE_01017 LMOSA_18470 nagB
OCPFDLNE_01118 LMOSA_19470 DLD
OCPFDLNE_01187 LMOSA_20100 hypothetical
OCPFDLNE_03080 LMOSA_7970 trxA
OCPFDLNE_01189 LMOSA_20120 eutP
OCPFDLNE_02952 LMOSA_6620 dhaK
OCPFDLNE_01021 LMOSA_18510 putativ
OCPFDLNE_01577 LMOSA_24510 yajC
OCPFDLNE_02141 LMOSA_510 mscL
OCPFDLNE_02453 LMOSA_3150 hdeD
OCPFDLNE_02454 LMOSA_3160 nagB
OCPFDLNE_02319 LMOSA_1480 iolW
OCPFDLNE_02107 LMOSA_180 sepF
OCPFDLNE_02426 LMOSA_2890 addA
OCPFDLNE_03010 LMOSA_7190 patB
OCPFDLNE_03018 LMOSA_7290 ABCF3
OCPFDLNE_01754 LMOSA_26060 perR
OCPFDLNE_01981 LMOSA_28280 cca
OCPFDLNE_00301 LMOSA_11830 nrdD
OCPFDLNE_02112 LMOSA_230 murG
OCPFDLNE_00404 LMOSA_12810 iolD
OCPFDLNE_00045 LMOSA_8730 arcC
OCPFDLNE_02349 LMOSA_1790 coiA
OCPFDLNE_01626 LMOSA_25000 pepQ
OCPFDLNE_02840 LMOSA_5590 dkgA
OCPFDLNE_02756 LMOSA_5460 ABC.CD.A
OCPFDLNE_03021 LMOSA_7320 celA
OCPFDLNE_02545 LMOSA_4010 licT
OCPFDLNE_01415 LMOSA_22890 recN
OCPFDLNE_00282 LMOSA_11600 E3.2.1.86B
OCPFDLNE_00884 LMOSA_17180 por
OCPFDLNE_00745 LMOSA_15960 fliC
OCPFDLNE_02073 LMOSA_29120 manXa
OCPFDLNE_02077 LMOSA_29160 gfrC
OCPFDLNE_00444 LMOSA_13260 fruAb
OCPFDLNE_03020 LMOSA_7310 E3.2.1.86B
OCPFDLNE_00443 LMOSA_13250 fruB
OCPFDLNE_03023 LMOSA_7340 bglK
OCPFDLNE_00442 LMOSA_13240 manR
OCPFDLNE_01302 LMOSA_21800 treB
T1 T2 T3 T4 T5 T6 T7 T8 T9
T1 T2 T3 T4 T5 T6 T7 T8 T9

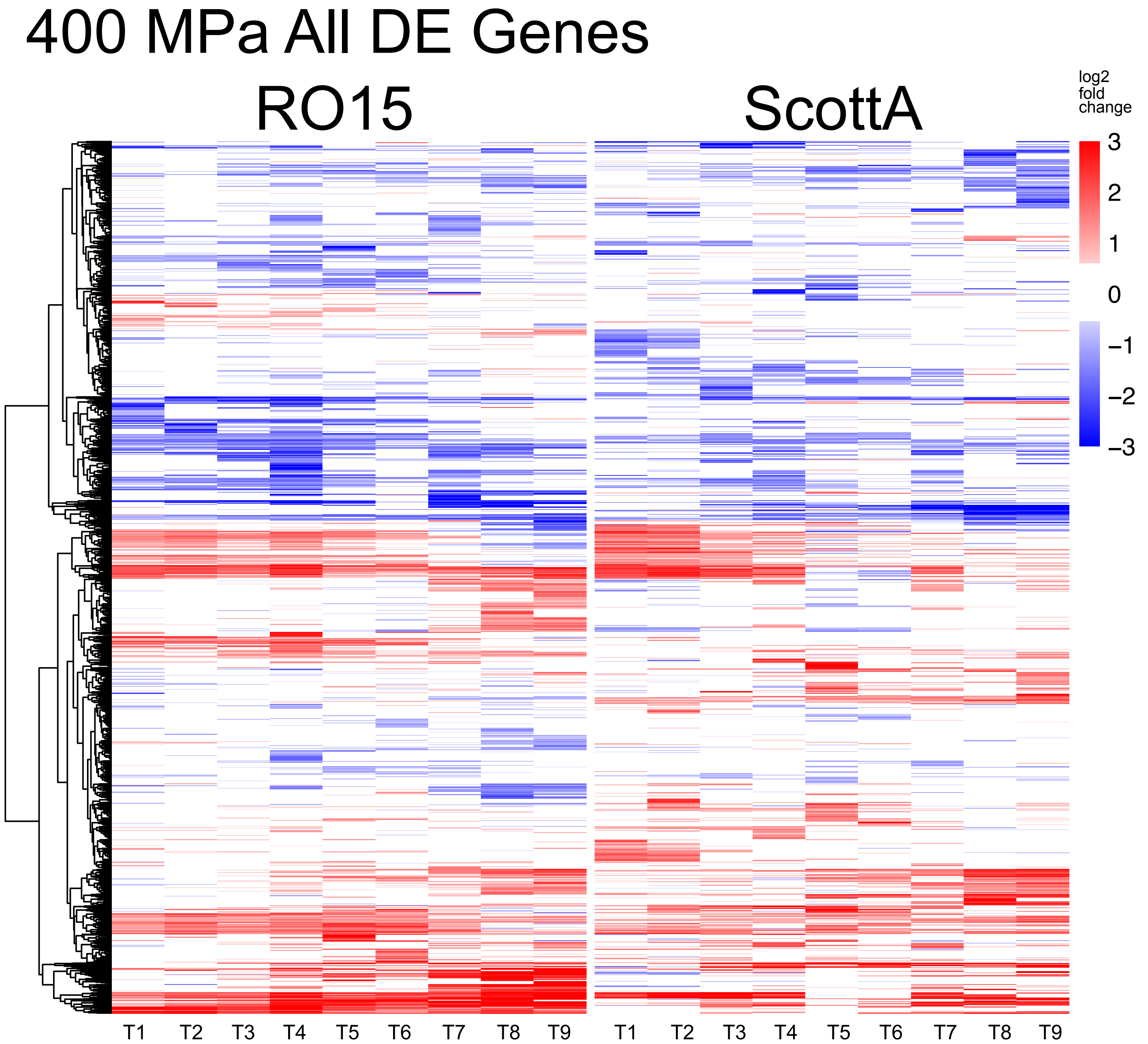
400 MPa All DE Genes
RO15
ScottA
log2 fold change
3
2
1
0
−1
−2
−3
T1
T2
T3
T4
T5
T6
T7
T8
T9
T1
T2
T3
T4
T5
T6
T7
T8
T9

400 MPa Cluster of interest 1

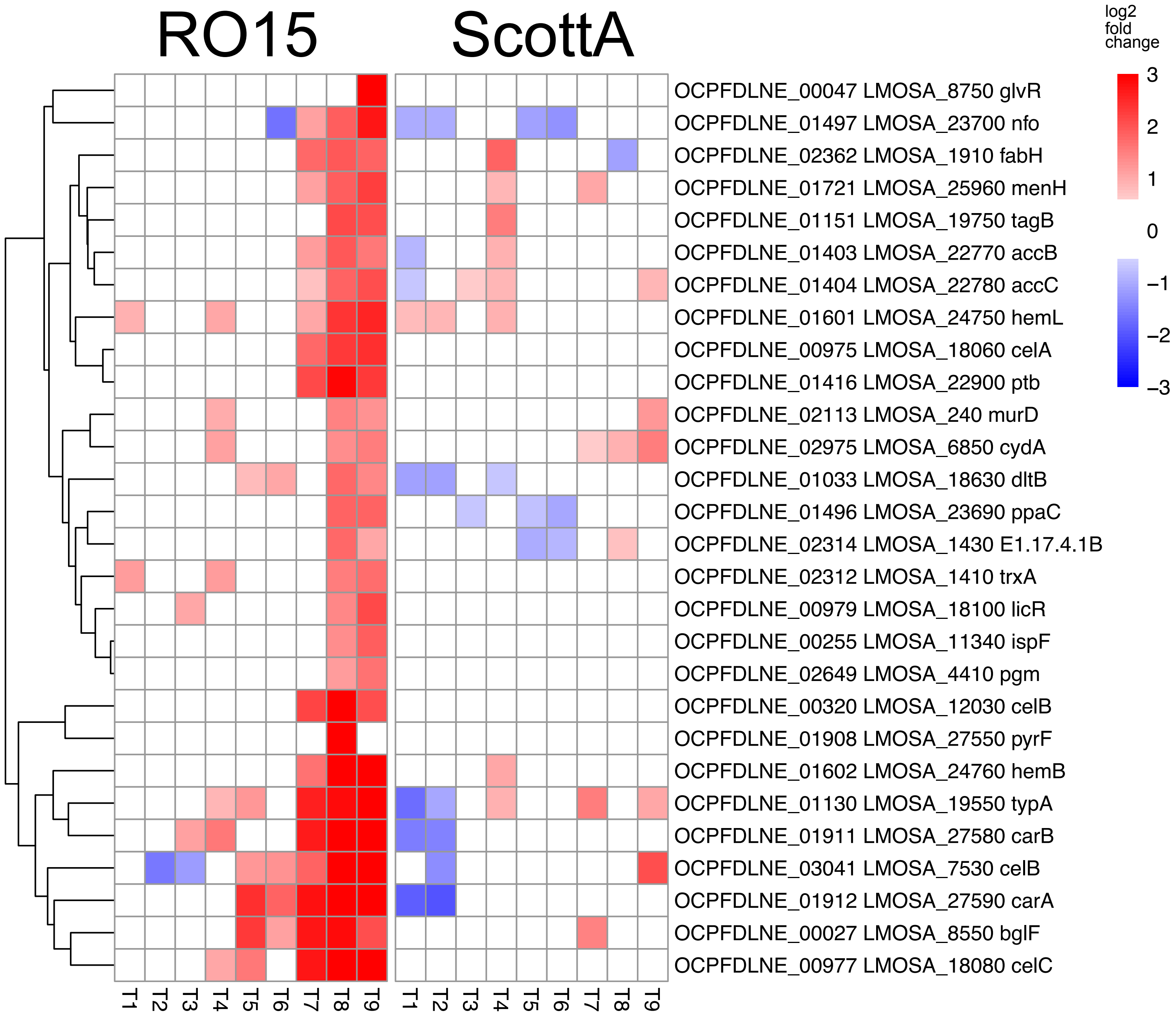

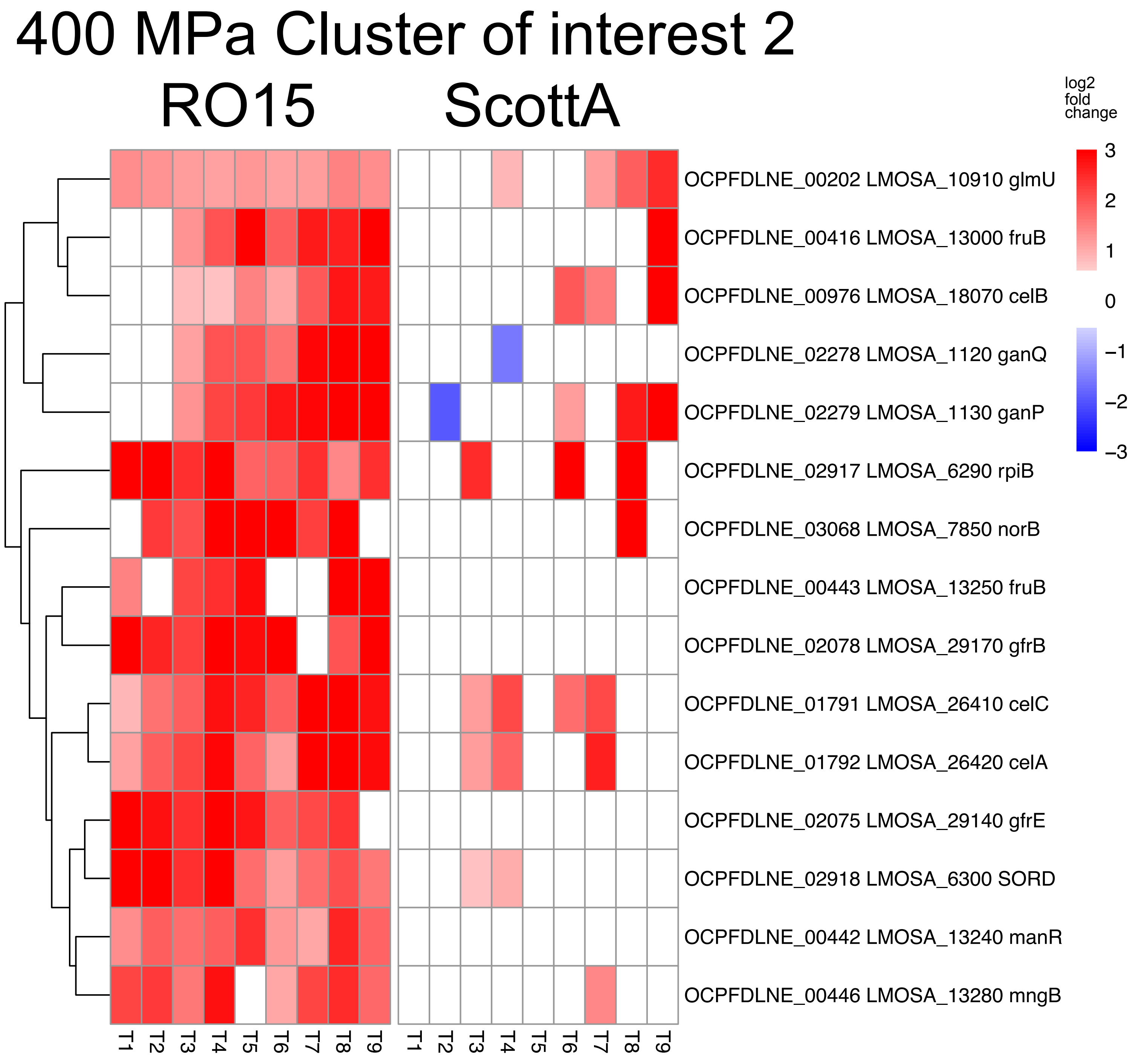
400 MPa Cluster of interest 2
RO15
ScottA
log2 fold change
3
2
1
0
−1
−2
−3
OCPFDLNE_00202 LMOSA_10910 glmU
OCPFDLNE_00416 LMOSA_13000 fruB
OCPFDLNE_00976 LMOSA_18070 celB
OCPFDLNE_02278 LMOSA_1120 ganQ
OCPFDLNE_02279 LMOSA_1130 ganP
OCPFDLNE_02917 LMOSA_6290 rpiB
OCPFDLNE_03068 LMOSA_7850 norB
OCPFDLNE_00443 LMOSA_13250 fruB
OCPFDLNE_02078 LMOSA_29170 gfrB
OCPFDLNE_01791 LMOSA_26410 celC
OCPFDLNE_01792 LMOSA_26420 celA
OCPFDLNE_02075 LMOSA_29140 gfrE
OCPFDLNE_02918 LMOSA_6300 SORD
OCPFDLNE_00442 LMOSA_13240 manR
OCPFDLNE_00446 LMOSA_13280 mngB
T1 T2 T3 T4 T5 T6 T7 T8 T9
T1 T2 T3 T4 T5 T6 T7 T8 T9

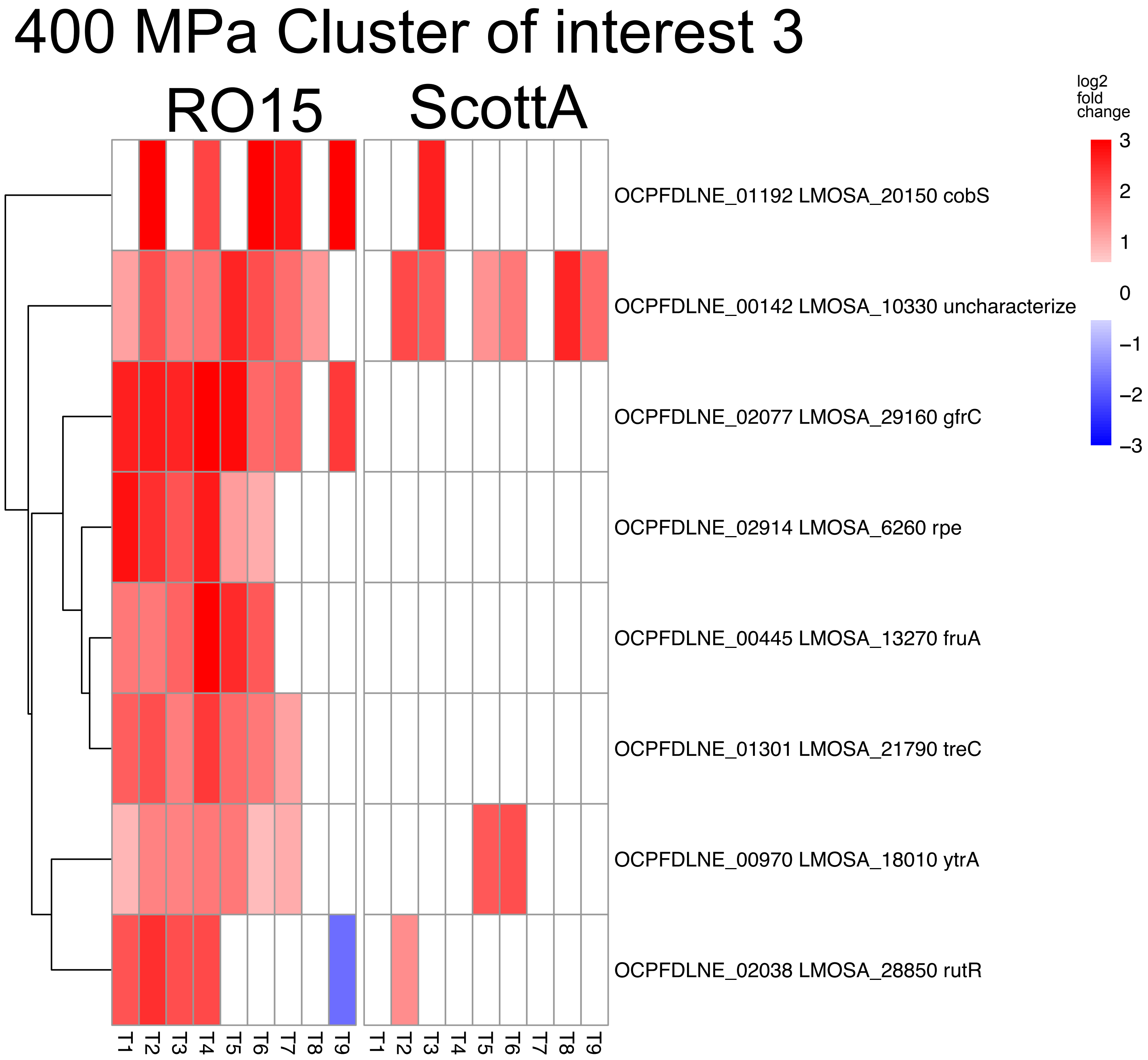
400 MPa Cluster of interest 3
RO15
ScottA
log2 fold change
3
2
1
0
−1
−2
−3
OCPFDLNE_01192 LMOSA_20150 cobS
OCPFDLNE_00142 LMOSA_10330 uncharacterize
OCPFDLNE_02077 LMOSA_29160 gfrC
OCPFDLNE_02914 LMOSA_6260 rpe
OCPFDLNE_00445 LMOSA_13270 fruA
OCPFDLNE_01301 LMOSA_21790 treC
OCPFDLNE_00970 LMOSA_18010 ytrA
OCPFDLNE_02038 LMOSA_28850 rutR
T1
T2
T3
T4
T5
T6
T7
T8
T9
T1
T2
T3
T4
T5
T6
T7
T8
T9

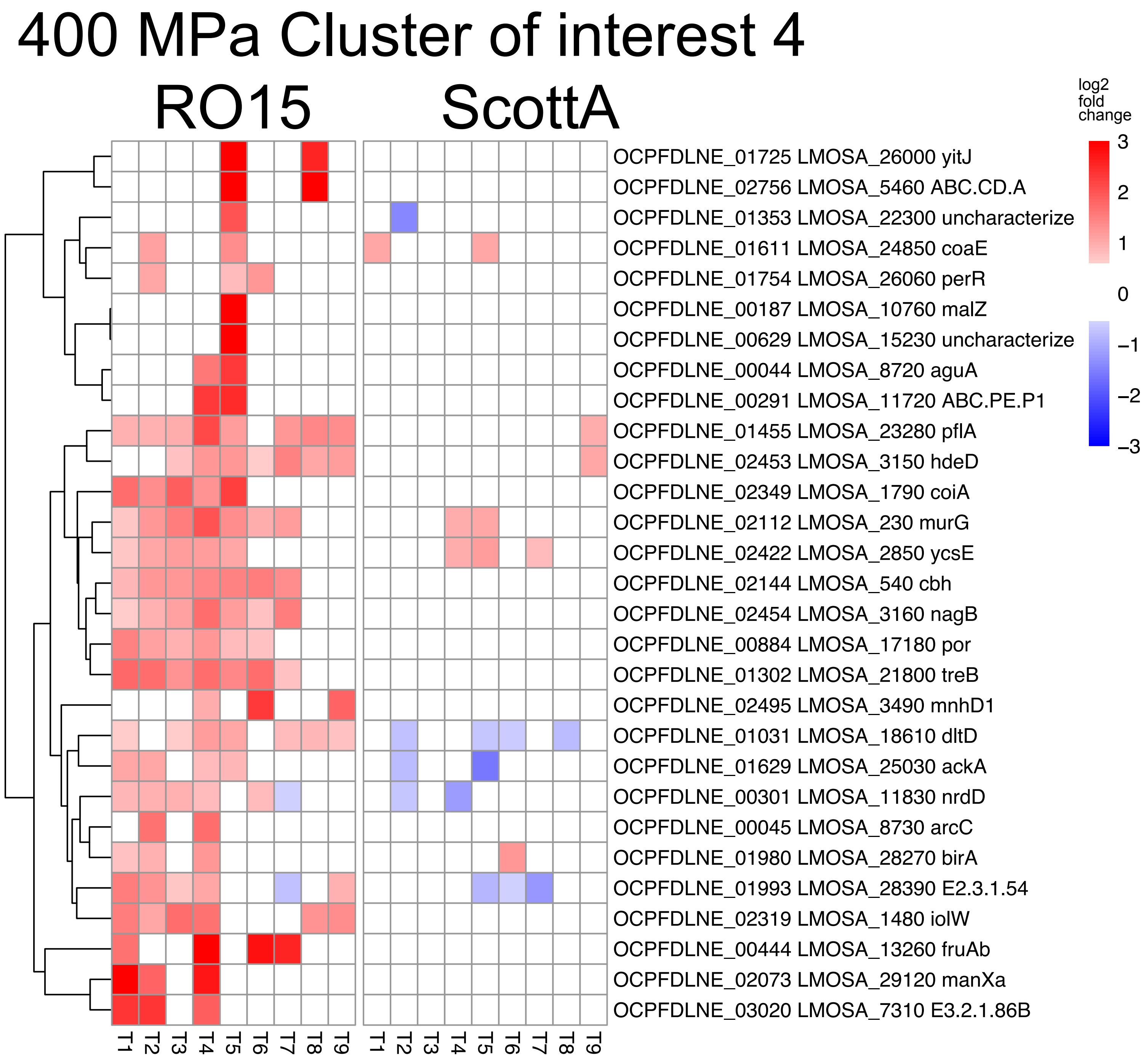
400 MPa Cluster of interest 4
RO15
ScottA
log2 fold change
3
2
1
0
−1
−2
−3
OCPFDLNE_01725 LMOSA_26000 yitJ
OCPFDLNE_02756 LMOSA_5460 ABC.CD.A
OCPFDLNE_01353 LMOSA_22300 uncharacterize
OCPFDLNE_01611 LMOSA_24850 coaE
OCPFDLNE_01754 LMOSA_26060 perR
OCPFDLNE_00187 LMOSA_10760 malZ
OCPFDLNE_00629 LMOSA_15230 uncharacterize
OCPFDLNE_00044 LMOSA_8720 aguA
OCPFDLNE_00291 LMOSA_11720 ABC.PE.P1
OCPFDLNE_01455 LMOSA_23280 pflA
OCPFDLNE_02453 LMOSA_3150 hdeD
OCPFDLNE_02349 LMOSA_1790 coiA
OCPFDLNE_02112 LMOSA_230 murG
OCPFDLNE_02422 LMOSA_2850 ycsE
OCPFDLNE_02144 LMOSA_540 cbh
OCPFDLNE_02454 LMOSA_3160 nagB
OCPFDLNE_00884 LMOSA_17180 por
OCPFDLNE_01302 LMOSA_21800 treB
OCPFDLNE_02495 LMOSA_3490 mnhD1
OCPFDLNE_01031 LMOSA_18610 dltD
OCPFDLNE_01629 LMOSA_25030 ackA
OCPFDLNE_00301 LMOSA_11830 nrdD
OCPFDLNE_00045 LMOSA_8730 arcC
OCPFDLNE_01980 LMOSA_28270 birA
OCPFDLNE_01993 LMOSA_28390 E2.3.1.54
OCPFDLNE_02319 LMOSA_1480 iolW
OCPFDLNE_00444 LMOSA_13260 fruAb
OCPFDLNE_02073 LMOSA_29120 manXa
OCPFDLNE_03020 LMOSA_7310 E3.2.1.86B
T1 T2 T3 T4 T5 T6 T7 T8 T9
T1 T2 T3 T4 T5 T6 T7 T8 T9
