# Supplementary material for: High-pressure processing-induced transcriptome response during recovery of Listeria monocytogenes
Source: BMC Genomics. 2021 Feb 12;22:117. doi: 10.1186/s12864-021-07407-6 (PMC7881616; doi:10.1186/s12864-021-07407-6)
Supplement: Supplementary file 32 — Additional file 32: Table S15. Primers used in ddPCR experiments. [file 12864_2021_7407_MOESM32_ESM.docx]

| **Target sequence** |  |  |  |  |  |
| --- | --- | --- | --- | --- | --- |
| **ScottA** |  |  | **Forward primer (5'-3')** | **Reverse primer (5'-3')** | **Amplicon (bp)** |
| LMOSA_23280 | Pyruvate formate-lyase-activating enzyme | *pflA* | gttttgggatgcatcaggtg | cggatcacgtgtgaaacaac | 151 |
| LMOSA_18880 | ATP-dependent Clp protease subunit | *clpE* | accagaagcactaacagcag | cgttcgctcaccgtattttc | 150 |
| LMOSA_10950 | Listeriolysin O | *hly* | cgtccatctatttgccaggt | aaagtgtagtgccccagatg | 153 |
| LMOSA_8820 | AgrB-like protein | *agrB* | gtttgtgcttgcgccatttg | attagcgtccctatcatcgc | 183 |
| LMOSA_4730 | Cell division ATP-binding protein | *ftsE* | atgaactttccggtggtgag | ctttgttgtgggttgccatc | 186 |
| LMOSA_5170 | UDP-N-acetylglucosamine 1-carboxyvinyltransferase | *murA* | gttgctgcgacagatttacg | tctacacgttccacatcagc | 158 |
|  | Chaperone protein dnaK | *dnaK* | ggatattccaccagctccac | gcgttagcttcagcatcttg | 189 |
| LMOSA_510 | Large-conductance mechanosensitive channel | *mscL* | gctgtagcggtagttatcgg | atggactggatgaaagcacc | 167 |
| LMOSA_6200 | Elongation factor G | *fusA* | cacccaatccaactcccaat | gcggtattcgtctgctaagt | 156 |
| LMOSA_27340 | ATP-dependent DNA helicase | *recG* | atgaggattatcggttgcgg | tgacttgcccttcagttgag | 150 |

**Supplementary Table 15. Primers used in ddPCR experiments.**
